# Measuring Mindfulness: A Psychophysiological Approach

**DOI:** 10.3389/fnhum.2018.00249

**Published:** 2018-06-28

**Authors:** Vladimir Bostanov, Lilian Ohlrogge, Rita Britz, Martin Hautzinger, Boris Kotchoubey

**Affiliations:** ^1^Institute of Medical Psychology and Behavioral Neurobiology, University of Tübingen, Tübingen, Germany; ^2^Department of Psychology, University of Tübingen, Tübingen, Germany

**Keywords:** mindfulness, mindfulness-based cognitive therapy, MBCT, concentration, event-related potentials, ERP, depression, t-CWT

## Abstract

Mindfulness-based interventions have proved effective in reducing various clinical symptoms and in improving general mental health and well-being. The investigation of the mechanisms of therapeutic change needs methods for assessment of mindfulness. Existing self-report measures have, however, been strongly criticized on various grounds, including distortion of the original concept, response bias, and other. We propose a psychophysiological method for the assessment of the mindfulness learned through time-limited mindfulness-based therapy by people who undergo meditation training for the first time. We use the individual pre-post-therapy changes (dERPi) in the event-related brain potentials (ERPs) recorded in a passive meditation task as a measure of increased mindfulness. dERPi is computed through multivariate assessment of individual participant's ERPs. We tested the proposed method in a group of about 70 recurrently depressed participants, randomly assigned in 1.7:1 ratio to mindfulness-based cognitive therapy (MBCT) or cognitive therapy (CT). The therapy outcome was measured by the long-term change (dDS) relative to baseline in the depression symptoms (DS) assessed weekly, for 60 weeks, by an online self-report questionnaire. We found a strong, highly significant, negative correlation (*r* = −0.55) between dERPi (mean = 0.4) and dDS (mean = −0.7) in the MBCT group. Compared to this result, the relationship between dDS and the other (self-report) measures of mindfulness we used was substantially weaker and not significant. So was also the relationship between dERPi and dDS in the CT group. The interpretation of dERPi as a measure of increased mindfulness was further supported by positive correlations between dERPi and the other measures of mindfulness. In this study, we also replicated a previous result, namely, the increase (dLCNV) of the late contingent negative variation (LCNV) of the ERP in the MBCT group, but not in the control group (in this case, CT). We interpreted dLCNV as a measure of increased meditative concentration. The relationship between dLCNV and dDS was, however, very week, which suggests that concentration might be relatively unimportant for the therapeutic effect of mindfulness. The proposed psychophysiological method could become an important component of a “mindfulness test battery” together with self-report questionnaires and other newly developed instruments.

## 1. Introduction

Mindfulness as a general concept and mindfulness meditation as a therapeutic method have become increasingly popular in the last decades and the number of research papers on the subject has grown exponentially (Williams and Kabat-Zinn, 2011). Mindfulness-based stress reduction (MBSR, Kabat-Zinn, 1990), mindfulness-based cognitive therapy (MBCT, Segal et al., 2002), and other related interventions have proved effective in reducing stress, anxiety, depression, and other clinical symptoms and in improving general mental health and well-being (Hofmann et al., 2010; Fjorback et al., 2011; Kuyken et al., 2015). The investigation of the mechanisms by which these therapeutic changes occur requires assessment methods, and, indeed, several self-report questionnaires have been developed for the purpose of measuring mindfulness (Baer, 2011). This approach has, however, been strongly criticized on various grounds, and it has been pointed out that existing questionnaires might not provide valid measures of mindfulness as defined by Buddhist sources and adopted by MBSR/MBCT (Grossman and Van Dam, 2011).

Mindfulness is a notoriously elusive concept and is hard to define. We will come back to this problem in the discussion. But for the time being, we will use a simple and fairly inclusive definition: *mindfulness is what is practiced in mindfulness meditation*. The circularity is not a joke—this is how mindfulness was defined in some of the most ancient Buddhist sources (Bodhi, 2011), and also, implicitly, by MBSR founder (Kabat-Zinn, 1990). In his original description of the program Kabat-Zinn (1990) did not provide any definition; recently (Kabat-Zinn, 2011), he stated that mindfulness had thus been defined by the whole book. Our definition emphasizes the experiential nature of mindfulness and the inherent difficulty of putting into words something that, ultimately, must be practiced in order to be understood. It also allows for an arbitrary length of the verbal description that would attempt to convey the meaning of the concept—originally, the Buddha's discourse on “The four establishments of mindfulness” (Bodhi, 2005; Ñanamoli and Bodhi, 2009); more recently, the MBSR and MBCT treatment protocols (Kabat-Zinn, 1990; Segal et al., 2002). It is also important to emphasize that our definition does *not* exclude mindfulness as practiced and developed in everyday life, without meditation. It just utilizes the fact that formal meditation provides an excellent opportunity to measure mindfulness in a controlled laboratory setting.

We also use another, clinically relevant, theoretical description by MBCT cofounder Teasdale (1999a), who defined mindfulness as the only “mode of mind” (Kabat-Zinn, 1990) that facilitates emotional processing and therapeutic change. The mindful mode is marked by “metacognitive awareness” (Teasdale, 1999b), the deep, intuitive, experiential understanding (or insight) that thoughts and emotions are passing mental events, and not the reality about the self, the world and the future. Teasdale (1999a) contrasted the mindful “being mode” to the habitual “doing mode” marked by problem-solving and achievement-oriented *thinking* characteristic of usual everyday activity. He also pointed up “rumination,” a cognitive style marked by circular thinking about one's physical and emotional state (Nolen-Hoeksema, 1987, 1991), as a particularly important example of the doing mode, because it is a well-known, central risk factor for depressive relapse/recurrence (Nolen-Hoeksema and Morrow, 1991; Segal et al., 2002; Donaldson and Lam, 2004, pp. 35–36). The modes of mind description is consistent with another influential model by Bishop et al. (2004), who also defined mindfulness as a mode (rather than a trait, as assumed by most self-report instruments), and additionally emphasized acceptance as an important therapeutic component of mindfulness (Hayes et al., 1999).

The Toronto Mindfulness Scale (TMS, Lau et al., 2006, see section 2.4.3 below), a questionnaire developed within the framework of the model of Bishop et al. (2004), is (presently, to the best of our knowledge) the only self-report instrument that measures mindfulness as a state (mode), rather than a trait (Baer, 2011, 2016). For this purpose it is administered immediately after meditation. It suffers, however, from the same limitations as all other questionnaires, like misunderstanding of items' meaning, response bias, and other impairments of objectivity inherent in self-report measures (Grossman and Van Dam, 2011).

With the present study, we propose an alternative to the self-report assessment of mindfulness. We define mindfulness as the mode of mind established during mindfulness meditation that has been learned in a standard MBSR/MBCT training. We assume that the difference between the mindful mode and the ordinary doing mode is represented by a difference in the event-related brain potentials (ERPs) recorded during meditation before and after the training (in participants with no previous experience with meditation). Further, we assume that, since mindfulness is a very complex and multifaceted concept, it is represented by different change patterns in different participants' ERPs, but the total amount of changes in each participant's ERP reflects how well he/she has learned to be mindful during meditation. Hence, a mathematical-statistical measure of the individual ERP change should predict therapy outcome. Obviously, such a psychophysiological measure of mindfulness is guaranteed to be free of any response bias and also does not suffer from the limitations of verbal expressions that may distort and misrepresent the subtle nature of mindfulness and, even when formulated accurately, may be misunderstood by participants.

In our previous studies (Bostanov et al., 2012), we used event-related brain potentials (ERPs) in an attempt to find a psychophysiological measure of meditative concentration. (For the difference between mindfulness and concentration, see the Discussion). ERPs are extracted directly from the electroencephalogram (EEG) and can reflect allocation of attentional resources in real time (Tecce, 1972; Pribram and McGuinness, 1992; Tecce and Cattanach, 1993). They can therefore be applied as direct psychophysiological measures of attention *during meditation* (Cahn and Polich, 2006; Ivanovski and Malhi, 2007). Moreover, ERPs can be elicited under passive conditions, i.e., *without an active task* (Polich, 1987; Baranov-Krylov et al., 2003), which makes them particularly valuable for measurements *in the being mode of mind* that can be easily disturbed by any active task. We designed a special “mindfulness ERP paradigm,” in which ERPs to neutral stimuli were recorded during meditation, after mood & rumination induction (Bostanov et al., 2012, see also sections 2.4.4, 2.4.6 below). We found that, after eight weeks of MBCT, the late contingent negative variation (LCNV) component of the ERP to an auditory test stimulus was significantly increased relative to both the pre-therapy baseline and a wait list control group. The LCNV amplitude is a direct, real-time measure of the excitation of neural pathways involved in conscious attentional processing and reflects the mobilization and allocation of attentional resources of limited capacity (Tecce, 1972; Tecce and Cattanach, 1993; Brunia and van Boxtel, 2001). Active CNV paradigms (see section 2.4.7 below) have been used to assess the concentration abilities of experienced meditators (Travis et al., 2000, 2002; Cahn and Polich, 2006). Therefore, we interpreted the pre-post-therapy change in LCNV (dLCNV) in our mindfulness paradigm as a measure of the increased concentration abilities of our participants after the training.

Originally, the goal of the present study was to replicate the dLCNV effect (Bostanov et al., 2012) in an improved mindfulness ERP paradigm (sections 2.4.4, 2.4.6) and in comparison to an active control group (section 2.5), and to test whether dLCNV predicts therapy outcome. Later, however, we recognized the possibility of achieving another, potentially more important goal, namely, to construct a *measure of mindfulness* based on *individual, multivariate ERP changes* (dERPi). For this purpose, we adopted a multivariate approach proposed by Bostanov (2015a), which allowed us to quantify the difference between the pre-therapy ERP and the post-therapy ERP by representing the single-trial ERPs as points in a vector space and computing the geometric distance between the two sample means for each participant (section 2.8.3). Note the strong emphasis on specific paradigm design in this approach: since *any* ERP change is interpreted as increase in mindfulness, such interpretation cannot rely on established results from the ERP literature (like in the case of LCNV), but is based (according to our definition of mindfulness) mostly on the fact that dERPi is measured under very specific, carefully designed conditions, namely, during mindfulness meditation (section 2.4.6) after mood & rumination induction (section 2.4.4), in a group of participants trained in mindfulness meditation (section 2.5). Further support for the interpretation of dERPi as increase in mindfulness can be provided by relationships (in the expected directions) between dERPi and other measures of mindfulness (section 2.4.3), and between dERPi and therapy outcome (section 2.6).

To summarize, the present study was *not* aimed at measuring the effectiveness of MBCT; its central goal was rather to develop a psychophysiological (ERP-based) measure of the mindfulness learned during the eight weeks of an MBCT course by recurrently depressed participants with no previous meditation experience. A secondary goal was to investigate further a previously found ERP correlate of meditative concentration.

## 2. Materials and methods

### 2.1. General design

**Figure 1:**
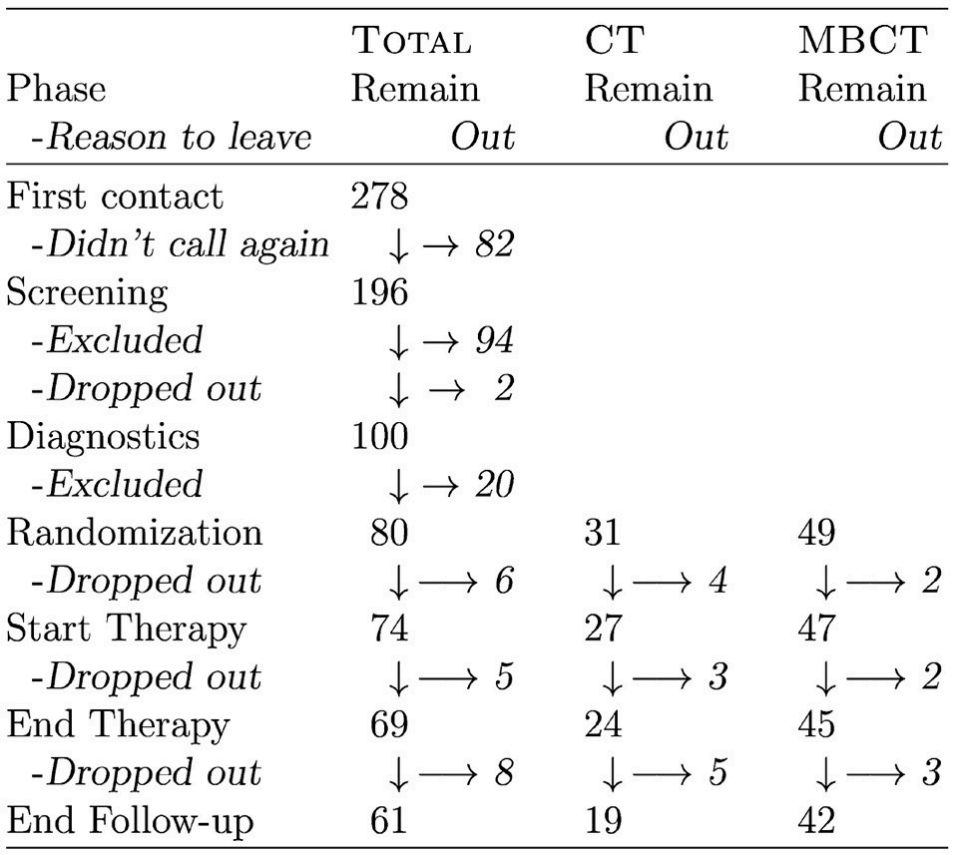
A flow chart showing the number of (prospective) participants in the different phases of the study. Candidates contacted us per phone or email; “Didn't call again” actually means that they either did not call again, or did not answer our email messages. Two prospective candidates refused further participation after screening, because they did not agree to randomization. Stated causes for drop-out before therapy were most often related to a new job offer or other reason for relocation. In two cases, however, the announced motivation for drop-out was dissatisfaction with study conditions and procedures. Drop-outs during therapy were mostly caused by illness (own or significant other's).

Prospective participants were subjected to a rigorous diagnostic procedure (section 2.3) and those who fulfilled the participation criteria (section 2.2) were randomly assigned to MBCT or group cognitive therapy (CT, section 2.5). Before and after the eight-week therapy course, they participated in an ERP experiment (section 2.4). Starting with the first week of therapy, they regularly gave self-report on the presence and severity of depression symptoms (DS) by filling a weekly online questionnaire (section 2.6) through a period of 60 weeks = eight weeks of therapy + 1 year follow-up. At the end of the follow-up period, they filled some final diagnostic questionnaires (section 2.3). These phases are also outlined in Figure 1. Preparations for the study started as early as September 2013. Patient screening began in March 2014. The first eight-week therapy group started in October 2014; the last one ended in April 2016. The last patient contact was in May 2017.

The general study design as well as the particular diagnostic, therapeutic, experimental and other procedures were approved by the Ethics Committee of the Medical School of the University of Tübingen. The study was also registered as DRKS00006014 at the German Clinical Trials Register (DRKS).

Initially, we intended to recruit at least 100 eligible recurrently depressed participants and used a simple computer generated random 1:1 sequence to assign participants to MBCT or CT. After having recruited about 40 participants, it became clear that we could not meet our goal and we changed the ratio to 2:1, because it was crucial to have enough participants in the MBCT condition in order to investigate the most important relationship between learned mindfulness and therapy outcome.

### 2.2. Participants

Recurrently depressed participants in stable remission were recruited through announcements in local newspapers and psychiatrists in private practice according to the same eligibility criteria as in our previous studies (Bostanov et al., 2012).

The inclusion criteria were: (a) age between 18 and 65 years, right-handedness, normal hearing ability; (b) history of recurrent major depression with three or more previous episodes (c) commitment to homework compliance and to not initiating any changes in psychiatric medication (starting, suspending, changing dosage) unless a relapse/recurrence made it necessary; (d) regular access to the internet and to an email account, signing informed consent after carefully reading a detailed description of their participation in the study.

The exclusion criteria were: (a) current major depressive episode, or presence of dysthymic disorder; (b) presence of substance abuse, eating disorder, or obsessive-compulsive disorder; (c) presence or history of one or more of the following: bipolar disorder, borderline personality disorder, schizophrenia or schizoaffective disorder, epilepsy or other neurological disorder, organic mental disorder, pervasive developmental delay; (d) significant experience with any kind of practice including mindfulness and/or concentration as important element (e.g., meditation, meditative prayer, autogenic training, meditative yoga, etc.).

Included participants payed us 65 euro as a token of their motivation. Those who completed all phases of the study (section 2.1) without dropping out, received 70 euro from us as a token of our gratitude.

Figure 1 is a flow chart showing the number of (prospective) participants in each phase of the study with the corresponding exclusion and/or drop-out rates.

**Figure 2:**
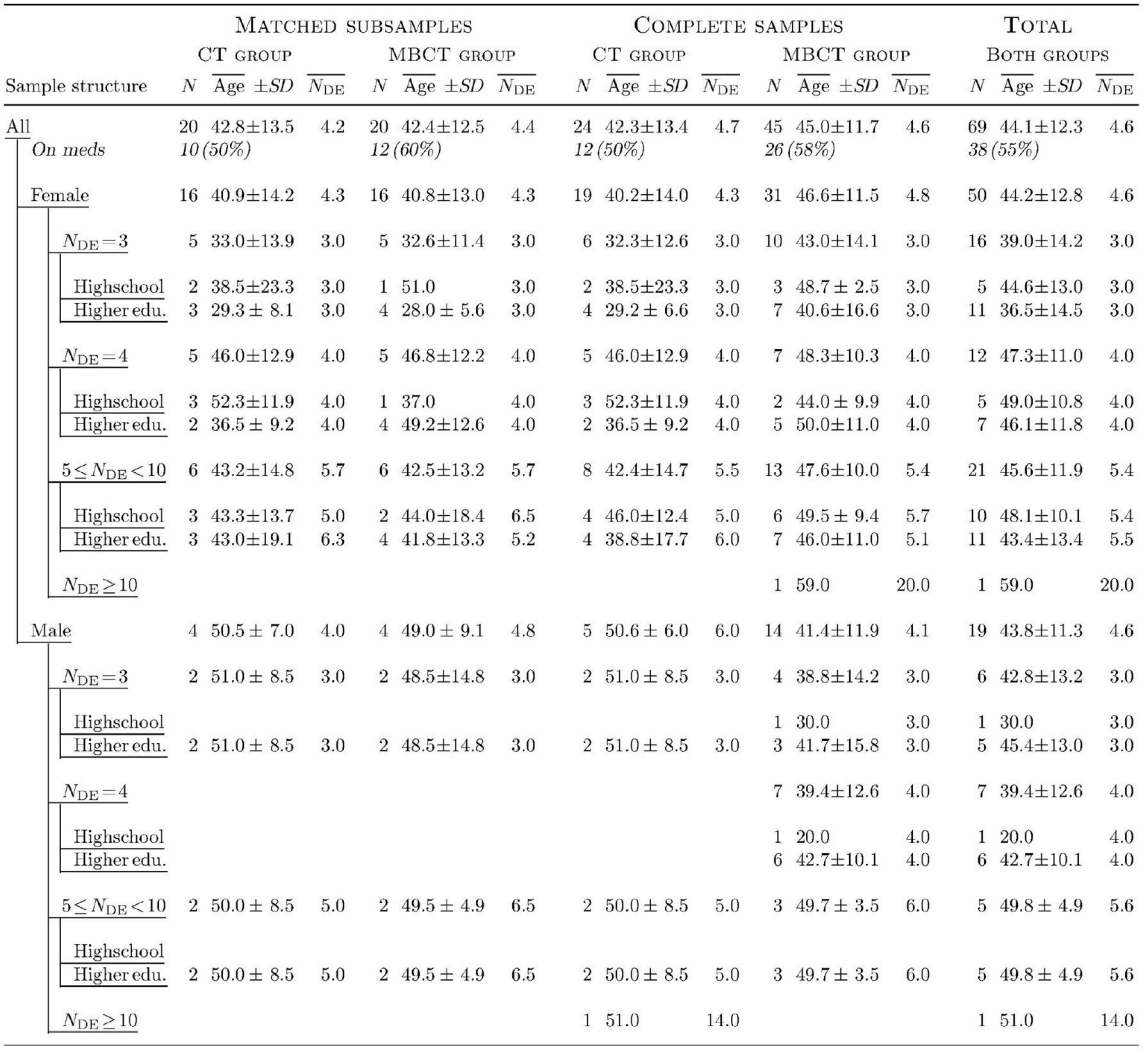
Detailed demographics of participant (sub)samples. *N* denotes the number of participants in the respective (sub)sample or layer, $\bar{\text{Age}}$ is the mean age, *SD* is the standard deviation of the age, and $\bar{N_{\text{DE}}}$ is the mean number of episodes of major depression. “Highschool” means completed German Realschule or Gymnasium and “Higher edu.” denotes higher education, but not necessarily completed (university students were included in this category). “On meds” refers to the number (and percentage) of participants taking psychopharmaceuticals during diagnostics and therapy. Medication included different kinds of antidepressants and, in two cases, anxiolytics and other psychoactive drugs.

In the statistical assessment of the results (section 2.8), we used two kinds of samples: the full CT group and the full MBCT group comprising the participants who completed therapy, and matched subsamples. The latter were constructed by looking for the best match from the MBCT group for each participant of the CT group. Participants were matched for sex, number of depression episodes (*N*_DE_), completed years of education, and age, in this order of priority. “Good” matching was possible in 20 cases. Participants were *not* matched for psychiatric medication taken during diagnostics and therapy, because of the variety of different drugs and dosages, and because of the many reported changes in medication (section 2.6) during the follow-up phase. The detailed demographics of the complete and the matched samples is presented in Figure 2.

In order to test the lab equipment and the whole experiment, and to train the lab assistants, we performed all diagnostic procedures (section 2.3) and the whole experimental session (section 2.4) with 21 students (major: mostly psychology; sex: 17 f, 4 m; age: mean = 23.3, std. dev. = 6.3) from our university, who received required credit points for their participation. They were included according to the same criteria as our recurrently depressed participants except for history of major depression which, in this case, served as an *exclusion* criterion. Thus, before we started working with recurrently depressed participants, we were able to test our equipment and our newly developed procedures, and to train our lab assistants (LO & RB) who conducted all further diagnostic and experimental sessions.

### 2.3. Diagnostics

All inclusion and exclusion criteria were first checked with prospective participants in a telephone screening interview. Those who passed the screening were sent an email containing a link to an admission web form, that they filled and submitted online. Then they received an email containing a link to a further personalized web form comprising the following four diagnostic questionnaires.

An online, German version of the Edinburgh Handedness Inventory (EHI, Oldfield, 1971) was used to include only right-handed participants (EHI score >70%). The EHI uses a five-point Likert scale to quantify the frequency of using the right or the left hand while performing 12 simple activities (e.g., writing, throwing things, brushing one's teeth, etc.).

An online, short (23 items), (German version Kühner et al., 2007) of the Response Styles Questionnaire (RSQ-D, Nolen-Hoeksema, 1991; Nolen-Hoeksema and Morrow, 1991), was used to assess the patients' *trait* tendency to react to negative mood by rumination. The RSQ has three sub-scales: Symptom-focused Rumination (8 items), Self-focused Rumination (7 items), and Distraction (8 items). Questions are answered on a four-point Likert scale ranging from “almost never” to “almost always.” The RSQ was administered after inclusion in the study and at the end of the 1-year follow-up period.

An online, German version (Michalak et al., 2008) of the Mindful Attention and Awareness Scale (MAAS, Brown and Ryan, 2003) was used to assess participants' *trait* mindfulness. The MAAS comprises 15 questions answered on a six-point Likert scale ranging from “almost never” to “almost always.” The reported internal consistency (Cronbach's alpha) of the German version is α = 0.83. The MAAS was also administered in the beginning of the study and at the end of the follow-up.

An online, German version (Wittchen et al., 1997) of the axis-II supplementary questionnaire to the Structured Clinical Interview (SCID, First et al., 1996), for the Diagnostic and Statistical Manual, fourth edition (DSM-IV, APA, 2000) was used for screening of personality disorders (PD). The questions are simply answered by “Yes,” “No,” or “Can't tell.” Only the parts of the questionnaire concerning PDs relevant for participation in the study were administered.

After filling and submitting the online forms, participants were invited to a personal diagnostic interview, in which current and previous major depression episodes and other mental disorders relevant for participation in the study were checked by a trained clinical psychologist (LO or RB) using the German version (Wittchen et al., 1997) of the DSM-IV SCID (First et al., 1996). While presence and history of major depression was questioned in detail, relevant PDs were addressed only if there were sufficient “Yes” answers in the corresponding section of the axis-II questionnaire. Current depression was additionally assessed with the German version (Hautzinger, 2013, chapter “Diagnostik”) of the Quick Inventory of Depressive Symptomatology, Clinician Rating (QIDS-C, Rush et al., 2003) which is quick and easy to use, and has, nevertheless, excellent psychometric properties (Trivedi et al., 2004).

Other diagnostic instruments used in the study which are of less relevance for the results presented here will be reported elsewhere (e.g., in the doctoral theses of LO & RB).

### 2.4. Experimental session

#### 2.4.1. General instruction

Participants were given a one-page written general instruction on a sheet of paper that they read during the lab assistant (LO or RB) prepared their head for the EEG recording (fixing EEG cap, attaching electrodes, etc.). The general instruction described shortly the duration and the structure of the whole experimental session, as well as the duration and content of the single procedures, tasks and questionnaires.

#### 2.4.2. Presentation of questionnaires, instructions and stimuli

Questionnaires were presented in a Mozilla Firefox web browser running in full-screen off-line mode. Each questionnaire was prepared as a HTML form using also CSS and JavaScript. The standard browser profile was modified to disable the invocation of the context menu by the right mouse button (in order to prevent participants from inadvertently doing something different from filling the form). The left mouse button was used by the participants to select their answers by clicking the corresponding radio buttons, and to finally submit them by clicking the form submission button. Upon submission, the answers, coded as an URL query string, were processed by JavaScript, which included validation and conversion into a HTML file that was saved on the hard disc by the lab assistant. The URL query string was also saved as a bookmark for backup. Participants had no access to the keyboard.

All instructions and statements in the mood & rumination induction (section 2.4.4) and in the meditation ERP tasks (sections 2.4.6, 2.4.7) were presented simultaneously in spoken and written form by playing MP4 video files with the VLC media player. These video files contained the spoken form as a sound track played through loudspeakers and the written form as synchronized subtitles presented on a pale gray-green background on a computer screen. VLC was configured to run full-screen with all user controls disabled (except for the ESC keyboard button that was only accessible by the lab assistant).

The auditory stimuli in the meditation ERP tasks (sections 2.4.6, 2.4.7) were presented by PsychoPy (version 1.77.01) scripts. These scripts also sent trigger signals to the EEG amplifier to record the presentation time by writing marks into the EEG event files.

The whole experimental session was controlled by a shell script running on the stimulation computer under Linux (Ubuntu 13.10). This script started the execution of the individual applications (Firefox, VLC, PsychoPy) in the specified order (Table 1) and with the corresponding data or media files as input, adjusted the sound volume as needed, and also started and stopped impedance check and EEG recording and storage on the EEG acquisition computer by sending signals over TCP/IP to the PyCorder application running there (section 2.4.5).

**Table 1:** An outline of the experimental session.

**Task name**	**Description**	**Duration**
Preparation and general instruction (section 2.4.1)	Attaching EEG electrodes, starting and testing equipment, and other preparation.Meanwhile, participants read the general instructions to the experimental session.	40 min
BDI (section 2.4.3)	Assessment of depression symptoms during the last 2 weeks.	4 min
PANAS (section 2.4.3)	Assessment of state affect and transient mood shifts.	3 min
Mood and rumination induction (section 2.4.4)	Evocation of sad mood and rumination by playing sad music and presenting depressing and ruminative statements.	14 min
PANAS	As above.	3 min
Passive meditation ERP task (section 2.4.6)	Participants practice mindfulness meditation with focus on their breath while their EEG is recorded. An auditory test stimulus is presented repeatedly, but participants are told that they do not need to attend to it.	25 min
TMS (section 2.4.3)	Self-report on the quality of mindfulness during the preceding meditation.	3 min
PANAS	As above.	3 min
Active meditation ERP task (section 2.4.7)	Patients practice mindfulness meditation with focus on their breath while their EEG is recorded. A block of two auditory test stimuli is presented repeatedly. Participants are instructed to always press a button after the first stimulus and then, depending on the quality of their meditation, also after the second stimulus.	32 min
TMS	As above.	3 min
	**Average total duration:**	**2 h 15 min**

#### 2.4.3. BDI-II, PANAS and TMS

The German version (Hautzinger et al., 2007) of the Beck Depression Inventory (BDI-II, Beck et al., 1996), a standard 21-item self-report questionnaire was used for the assessment of residual symptoms of depression at the time of the experimental sessions in order to make sure that participants were still not acutely depressed after the diagnostics, and also to control for the influence of depression symptoms on participants' ERPs. For the latter purpose, we used the pre-post-therapy change (dBDI) in each participant's mean BDI score.

The German version (Krohne et al., 1996) of the Positive and Negative Affect Schedule (PANAS, Watson et al., 1988), a standard 20-item self-report questionnaire used for assessment of transient mood shifts on a five-point Likert scale was administered immediately before and after the mood induction, and a then after the passive meditation ERP task.

The TMS (Lau et al., 2006) is a 13-item self-report questionnaire designed for assessment of the quality of *state* mindfulness during meditation on a five-point Likert scale. We administered it immediately after the end of each meditation ERP task (sections 2.4.6, 2.4.7). It comprises two sub-scales: Decentering (TMSd, 7 items) and Curiosity (TMSc, 6 items). Lau et al. (2006) found that, while pre-post-therapy changes (dTMSd) in TMSd significantly predicted therapy outcome after MBSR, this was *not* true for changes (dTMSc) in TMSc. We computed both dTMSd and dTMSc for each participant in each ERP task (sections 2.4.6, 2.4.7) in order to assess learning effects and relationships with the therapy outcome measure (dDS, section 2.6). To the best of our knowledge, at the time of this writing, there is still no published validated German translation of the TMS. We used our own translation of the instructions and the questions.

#### 2.4.4. Mood & rumination induction

In our *previous* study (Bostanov et al., 2012), we used separate procedures for mood induction and rumination challenge; the mood statements were presented *before* the meditation task while the rumination instructions were presented *during* the meditation and the ERP recording. In the *present* study we combined the mood induction and the rumination challenge in a single procedure performed *before* the passive mindfulness meditation ERP task (see Table 1). We used again our German translation of the mood induction instructions used by Broderick (2005), but we extended 17 of the 20 mood statements by rumination statements invented by us, partly inspired by those of Lyubomirsky and Nolen-Hoeksema (1993). For instance, statements 2, 6, 9, and 12 read, respectively (the rumination extensions are emphasized): “I feel rather sluggish now *and I don't know why*.”; “I just don't seem to be able to get going as fast as I used to. *I wonder what's wrong with me*.”; “My life is so tiresome—the same old thing day after day depresses me. *I wonder how much longer I can stand it*.”; “It often seems that no matter how hard I try, things still go wrong. *There must be some reason for this, but which is it? Am I doing something fundamentally wrong? Am I being punished? But why? For what?*” As in Bostanov et al. (2012), all instructions and statement were presented in both written and spoken form while depressing music was played in the background, and the procedure ended with a mood incubation phase (Zoellner et al., 2003). There were some improvements though: the spoken statements were recorded by a professional actress, the written instructions and statements were presented on a computer screen (see section 2.4.2), and we used different background music. For the mood-rumination statements and the incubation phase we used the first 15 s of “Laura Palmer's Theme” from Angelo Badalamenti's sound track of the TV Series “Twin Peaks,” repeated in a loop, as background music. A 40-s loop taken from the “Prelude” of Alan Parson's Project “Tales of Mystery and Imagination” was played in the background during the presentation of the instructions. The spoken instructions were recorded by one of us (LO). The procedure started with about 2-min long initial instructions followed by a 9-min presentation of the 20 statements, and ended with the 3-min incubation phase (1 min instruction, 2 min music). The total duration was 14 min.

#### 2.4.5. EEG recording

EEG digitized at 500 Hz was recorded continuously during both meditation tasks using Ag/AgCl electrodes attached to 31 scalp sites: Fp1, Fz, F3, F7, FT9, FC5, FC1, C3, T7, TP9, CP5, CP1, Pz, P3, P7, O1, O2, P4, P8, TP10, CP6, CP2, Cz, C4, T8, FT10, FC6, FC2, F4, F8, and Fp2 (according to the 10-20 system), referenced to the nose. Electrooculographic (EOG) signals were recorded using bipolar channels from the following four sites: lateral orbital rims (for horizontal eye movements) and left supra- and infraorbital sites (for vertical eye movements and eye blinks). The recordings were performed with Brain Vision hardware (actiCHamp) and software (PyCorder) run under Microsoft Windows 7.

#### 2.4.6. Passive mindfulness meditation task

The passive meditation ERP task comprised a 3-min instruction in the beginning followed by a 22-min stimulus presentation phase. The instruction was effectively a compressed version of the standard mindfulness-of-the-breath meditation instruction used in the MBCT course. Participants were advised to find a body part or region where they could feel the in- and out-breath and concentrate on these sensations (e.g., at the abdominal wall, or in the nostrils, or at the upper lip, etc.) without trying to modify or control the breath. Distracting thoughts, feelings and other sensations were declared as completely normal and some guidance was given on how to respond to them mindfully. The instruction ended with a short notice telling participants that they were going to hear the following noise (the stimulus was presented once at this point) repeatedly during their meditation, and that they did not need to pay extra attention to it, but should just stay focused on their breath as good as they can. The instruction was delivered in both written and spoken form (see section 2.4.2). The stimulus was very similar to the one we used in our previous study (Bostanov et al., 2012): a 1.5-s long white noise sample with a 50-ms linear fade-in and a 10-ms linear fade-out followed by 0.1 s of silence. It was presented 90 times via loudspeakers at 45 dB SPL (measured at the position of the participant's head) in a fixed sequence that was the same for all participants and in both experimental sessions (pre- and post-therapy). The length of the inter-stimulus interval was drawn from a uniform random distribution within a range that increased linearly with the number in the sequence—the range was 4–8 s at the beginning, 5.2–10.5 s after the tenth stimulus, and 14–28 s at the end of the sequence (the lower bound was always half the upper bound). A trigger signal was sent to the EEG amplifier at each stimulus onset and was recorded into the EEG event file.

#### 2.4.7. Active mindfulness meditation task

In the active ERP task, the participants were told to continue their mindfulness meditation on the breath but they were also instructed how to react to the presented stimuli by pressing a mouse button. The stimulus sequence was fixed and comprised 50 presentations of the same two-stimulus block with an inter-block interval drawn from a uniform random distribution within the fixed range 20–40 s. The block consisted of a long tone, followed by a 2-s silent response window, followed by a short tone, followed by a second 2-s silent response window. The long tone was an authentic 1.5-s long organ chord (Cm) sample with a 50-ms linear fade-in and a 10-ms linear fade-out. The short tone was a 0.1-s long organ chord (Db) sample with a 10-ms linear fade-in and a 10-ms linear fade-out. Both tones were presented via loudspeakers at 40 dB SPL (measured at the position of the participant's head). A trigger signal was sent to the EEG amplifier at the beginning of the stimulus block (i.e., at the onset of the long tone). Participants were instructed to hold the mouse in their right hand and to press the right button with their thumb after every presentation of the long tone and then to press it again after the short tone, but only if their attention was focused on the breath as they heard the long tone; they were told not to react a second time if they had been lost in thought when the tone had come. Both variants were demonstrated in the instruction by presenting the stimulus block with one or two mouse-click sounds added. The auditory presentation was accompanied by a visual presentation, e.g.,: “[long tone] [click] [short tone].” Then, participants were invited to practice both kind of responses under the supervision of the lab assistant. The stimulus block was presented 11 times preceded by an announcement specifying whether participants should assume they were focused on the breath or distracted by thoughts and react accordingly. The lab assistant gave feedback on wrong responses and in some cases (if no sufficient learning was apparent) the practice phase was repeated. The duration of the instruction (including practice) was 4 min and that of the stimulus-block sequence was 28 min (total duration: 32 min).

The active mindfulness meditation task was essentially a combination of a classical CNV paradigm and the mindfulness assessment paradigm developed by Burg and Michalak (2011). The motor response to the end of the first stimulus was used both to elicit a classical CNV and to control whether participants were awake, alert and competent and, hence, reacting adequately. We assumed that those who responded less than 40 times to the first stimulus had either fallen asleep or had not understood the instruction after all, and their data were excluded from further processing.

### 2.5. Therapy

MBCT was delivered by one of us (VB), a trained and certified MBCT therapist, with more than 12 years of own mindfulness meditation practice, following the standard protocol (Segal et al., 2002) using the standard German translation of the handouts (Segal et al., 2008). MBCT comprises eight weekly 2-h group sessions (eight to twelve participants) and at least 45 min of daily homework (6 days a week) over the eight weeks. Participants are first trained in sustained focused attention to the breath and to other bodily sensations. Later, still using the breath as an anchor for concentration, they are taught to include thoughts and emotions as objects of mindful attention and learn to perceive them as mental events and not as absolute truth, self or reality (metacognitive awareness).

CT was delivered by a trained and certified CT therapist with no experience with any kind of mindfulness, following a German adaptation (Hautzinger, 2010, 2013; Risch et al., 2012) of the the protocol by Bockting et al. (2005) based on Jarrett et al. (2001). This protocol is particularly suitable for comparison with MBCT, because of the almost identical format (group size, number, frequency and length of sessions, homework load, etc.). It contains all CT elements included in MBCT, but no mindfulness components. The treatment is manualized and has been shown to be effective in comparison to TAU (Jarrett et al., 2001; Bockting et al., 2005).

### 2.6. Weekly assessment of depression symptoms (DS)

Participants' depressive symptoms (DS) were assessed weekly during the course of therapy and during the 1-year follow-up period. We used the German version (Hautzinger et al., 2012) of the 15-item Center for Epidemiologic Studies Depression Scale (CES-D, Radloff, 1977), which measures on a four-point Likert scale the presence and the severity of DS (including somatic symptoms and interpersonal problems) during the last week. It has excellent psychometric properties, is more suitable than the BDI (section 2.4.3) for the assessment of sub-clinical depression, and is also very user-friendly, compact, and quick and easy to fill, which makes it perfectly suitable for a long-period continual assessment (Ensel, 1986; Radloff and Locke, 2000). We used the long-term change (dDS) in mean DS score as a central outcome measure. dDS was computed as the difference between the meen DS score over the 52-week follow-up period (DSf) and the mean DS during the 8 weeks of training (DSt). The DS questionnaire contained also an additional question regarding changes in psychiatric medication (section 2.2).

Participants filled the DS questionnaire weekly, online. Every Monday morning about 2:30 a.m. they were sent an automatically generated email message including a link to a personalized web form including the participant's ID (hidden) and the week number (counted from the beginning of therapy). If they did not fill and submit the questionnaire til Thursday 2:30 a.m. they were sent a reminder email; if they still did not fill it til Saturday 2:30 a.m., they got a second reminder. The whole procedure was controlled by a cron job and a PHP script running on a remote server. Valid data were stored on the server, invalid submissions were immediately sent back to the participant with the unanswered questions marked.

### 2.7. Weekly report on the amount of mindfulness practice (AMP)

The DS questionnaire for the MBCT group contained an additional question regarding the days per week and average minutes per day that participants spent practicing mindfulness. We introduced some non-linearity into the scoring of the amount of mindfulness practice (AMP, Table 2) reducing the weight of average durations of less than 15 min/day, and increasing the importance of regular shorter exercises relative to infrequent longer meditations. Such scoring reflects the predominant opinion on the significance of frequency and average duration of exercise in the mindfulness meditation community.

**Table 2:** The scoring scheme for the assessment of the amount of mindfulness practice (AMP) in the MBCT group; mpd denotes minutes per day, dpw means days per week.

**Minutes per day**	**AMP scores**		
	**1 or 2 dpw**	**3 or 4 dpw**	**5 to 7 dpw**
Up to 10 mpd	1	5	10
15 to 30 mpd	4	20	40
More than 35 mpd	6	30	60

### 2.8. Statistical assessment

#### 2.8.1. ERP preprocessing

The EEG datasets were preprocessed with EEGLAB 13.4.4b for MATLAB. First, 3.1-s-long epochs starting 100 ms before stimulus onset were extracted from the contious datasets (in the active task, the onset of the long tone was used for reference). Then, EOG artifacts were removed from these ERP segments by REGICA 1.00, an EEGLAB implementation of the hybrid REG-ICA method (Klados et al., 2011) combining regression (REG) and blind source separation based on independent component analysis (ICA). Finally, the ERPs were referenced to the 100-ms pre-stimulus baseline. In both ERP tasks, the first 10 stimuli were ignored; in the passive task (section 2.4.6), the last epoch could not be created because the task ended too soon after the last stimulus (due to a programming error). Thus, 79 ERP trials from the passive task and 40 trials from the active task were available for further assessment.

#### 2.8.2. Univariate assessment

In order to replicate our previous finding (Bostanov et al., 2012), we computed the pre-post-therapy change (dLCNV) in the late contingent negative variation ERP component (LCNV) for each participant as the difference between the mean voltage (area) in the time interval 1.3–1.5 s over all channels and all trials in the passive ERP task after the therapy and its corresponding value before therapy. These dLCNV values were subjected to Student's *one-sided t*-tests, because we formulated a directed hypothesis based on our previous results (Bostanov et al., 2012), namely that dLCNV should be larger in the MBCT group than in the CT group. Note, however, that this kind of ERP assessment was *not* purely univariate, because we used the PCA-based multivariate procedure outlined below (section 2.8.3) for artifact rejection.

We used *two-sided t*-tests to compare to zero and with each other the dERPi values (see section 2.8.3, below) as well as other pre-post-therapy differences and long-term changes in questionnaire scores obtained from the two therapy groups. Analyses of covariance (ANCOVAs) were also used (where applicable) to compare group means taking into account the baseline levels of the compared measures in each therapy group. All these comparisons were performed with both the full samples and the matched subsamples (Figure 2). In the latter case, when data were missing or were excluded as outliers in one subsample, the corresponding matched participants from the other subsample were also excluded in order to preserve the matching. Bonferroni corrections of the resulting *p*-values were used to control for accumulation of chance by multiple comparisons.

The relationships between different measures were assessed by Pearson correlations, *r* and, where needed, also by partial correlations, *r*_p_ with the effects of dBDI, dPA, and dNA removed, where dBDI was the pre-post-therapy change in BDI-score, and dPA and dNA were the corresponding changes in baseline positive and negative affect scores (section 2.4.3). Student's *t*-tests were used to check whether *r* and *r*_p_ were respectively different from zero. Fisher's *z*-tests were used to compare *r* and *r*_p_ in the MBCT group to their corresponding values in the CT group. Bonferroni corrections of the resulting *p*-values were used to control for accumulation of chance by multiple comparisons.

The central therapy outcome measure dDS was defined as: dDS = DSf − DSt (section 2.6). The latter, DSt, is, however, not a very good baseline measure, because it might reflect participants' reactions to the therapy. In order to monitor the possibility of such effects, we additionally performed the most important assessments including dDS with DSf instead of dDS and then also with DSt.

Recurrence/relapse rates were computed from the weekly DS scores during the 1-year follow-up period. Recurrence/relapse was defined by the DS score being larger than 17 for longer than two weeks in a row. This DS cutoff score was obtained from German validation studies of the CES-D scale as the value providing optimal specificity and sensitivity (Hautzinger et al., 2012). The two-week period is defined by the DSM-IV criteria for a Major Depression Episode (APA, 2000).

#### 2.8.3. Multivariate assessment

The datasets obtained from each ERP task were subjected to individual multivariate assessment as outlined by Bostanov (2015a) using the t-CWT toolbox for MATLAB (Bostanov, 2015b). For each participant, the pre-therapy and the post-therapy data were merged into one dataset comprising both samples of trials. These ERP trials were treated as points in a multidimensional vector space (Rencher, 1998). The logarithm of *D*^2^, the (squared) Mahalanobis distance (Rencher, 1998, p. 22) between the pre-therapy sample mean and the post-therapy sample mean was taken as an *individual* psychophysiological measure of change (dERPi) in the participant's brain response to the test stimuli during the meditation tasks. *D*^2^ is similar to the usual distance between two points in physical space, but with a different scale applied in each dimension, because the natural unit of measurement is the standard deviation of the principal component corresponding to that dimension (Bostanov, 2015a). Hotelling's well-known two-sample *T*^2^-statistic is just *D*^2^ multiplied by *N*_1_*N*_2_/(*N*_1_+*N*_2_), where *N*_1_ and *N*_2_ are the respective sample sizes (Rencher, 1998, p. 87). Because distance is always positive, we took its logarithm, dERPI = log(*D*^2^), which has a more symmetric distribution.

The Mahalanobis distance *D*^2^ can be represented in the form of a scalar product (Bostanov, 2015a, Equations 12 and 57):

$$D^{2}=\overset{K}{\underset{k=1}{\sum }}\overset{T}{\underset{0}{\int }}{\text{dERP}}_{k}\left(t\right){\text{LDF}}_{k}\left(t\right)dt,$$

where *k* is the channel index, *K* is the number of EEG channels, *t* is the time, *T* is the length of the time interval, dERP_*k*_(*t*) is the mean pre-post-therapy ERP difference, and LDF_*k*_(*t*) is the linear discriminant function (Rencher, 1998, p. 74). This representation is useful, because both dERP and LDF can be visualized in order to inspect which ERP features or components contribute to *D*^2^.

In the case of ERP data, the dimensionality of the vector space must be reduced immensely before *D*^2^ can be computed. In t-CWT, this is achieved by a frequency domain representation and filtering of the data, principal component analysis (PCA), t-CWT feature extraction, a second PCA, and step-down selection of principal components. In the present study, we did each assessment twice, with and without t-CWT feature extraction. Furthermore, in order to take into account also non-phase-locked event-related activity (Pfurtscheller and Da Silva, 1999), we computed the logarithm of the EEG power spectrum (in the chosen time window, see below) and performed an additional assessment (without the t-CWT step) in the extended vector space that included these logarithmic amplitudes for each frequency and channel. It is important to emphasize, that both the step-down selection and the optional t-CWT feature extraction do much more than just general dimensionality reduction for computational purposes—they also specifically reject variables that do not contribute to the difference (i.e., do not significantly increase *D*^2^) between the two samples.

Multivariate assessment was also used to check the significance of the changes in PANAS scores (section 2.4.3) after the mood & rumination induction (section 2.4.4) and after the passive meditation ERP task (section 2.4.6) by Hotelling's *T*^2^-tests on the pairs of positive and negative affect scores.

#### 2.8.4. Computational details

For the frequency domain representation of the ERPs (Bostanov, 2015a), we used a 2-s modified Tukey window starting at stimulus onset with 20-ms fade-in time and 200-ms fade-out time, and cutoff frequencies *f*_*c*_ = 10, 15, and 20 Hz. Fourier components corresponding to frequencies *f*>2*f*_*c*_ were deleted, those with frequencies *f*_*c*_<*f* < 2*f*_*c*_ were attenuated linearly. In the subsequent PCA-based multivariate outlier rejection procedure (Bostanov, 2015a), principal components explaining 99% of the total variance and ERP vectors lying not further than σ__*D*__*C*_σ_ from the mean were retained, where σ__*D*__ was the standard deviation of *D*, the Mahalanobis distance to the mean, and *C*_σ_ took the values *C*_σ_ = 4, 4.5, 5, and 9. The second PCA, after t-CWT feature extraction or directly after outlier rejection used the same 99% cutoff criterion. The overall α-level in the subsequent step-down test (Rencher, 1998, pp. 111, 177, 217) for the selection of the final principal components was set to α = 0.3. The Mahalanobis distance *D*^2^ between the pre-therapy ERP and the post-therapy ERP was computed from these selected components.

The code of the *published* version t-CWT 2.01 (Bostanov, 2015b,c) was almost fully vectorized with one notable excellent: the procedure that finds the local extrema of the *t*-value scalogram was vectorized in the time dimension but not in the scale dimension where it used for-loops. The reason for this was that the scalogram was sampled on a log-grid (Bostanov, 2015a). Historically, this solution was chosen for saving RAM in a time when memory resources were scarce. In the current, *previously unpublished*, version of t-CWT 3.00 (Bostanov, 2017a,b), we replaced the log-grid by a semi-logarithmic grid which allowed for complete vectorization of the code and tremendous speed up of the computations. Nevertheless, we used the high-performance computing (HPC) resources of the bwUniCluster at the Karlsruhe Institute of Technology provided by the bwHPC project for all t-CWT assessments. The t-CWT scripts were run with MATLAB 8.4.0.150421, (R2014b) using Statistics Toolbox 9.1 under Linux (RHEL 7.3).

## 3. Results

We do not report the ERP data obtained from our never-depressed participants, because they are not important for the purpose of the study. We do report, however, their PANAS data because they demonstrate the effectiveness of our mood & rumination procedure even with less motivated never-depressed participants.

### 3.1. Relapse/recurrence rates

The obtained DS-based relapse/recurrence rates were: 18 (42.8%) of 42 in the MBCT group, and 7 (36.8%) of 19 participants in the CT group.

### 3.2. PANAS

**Figure 3:**
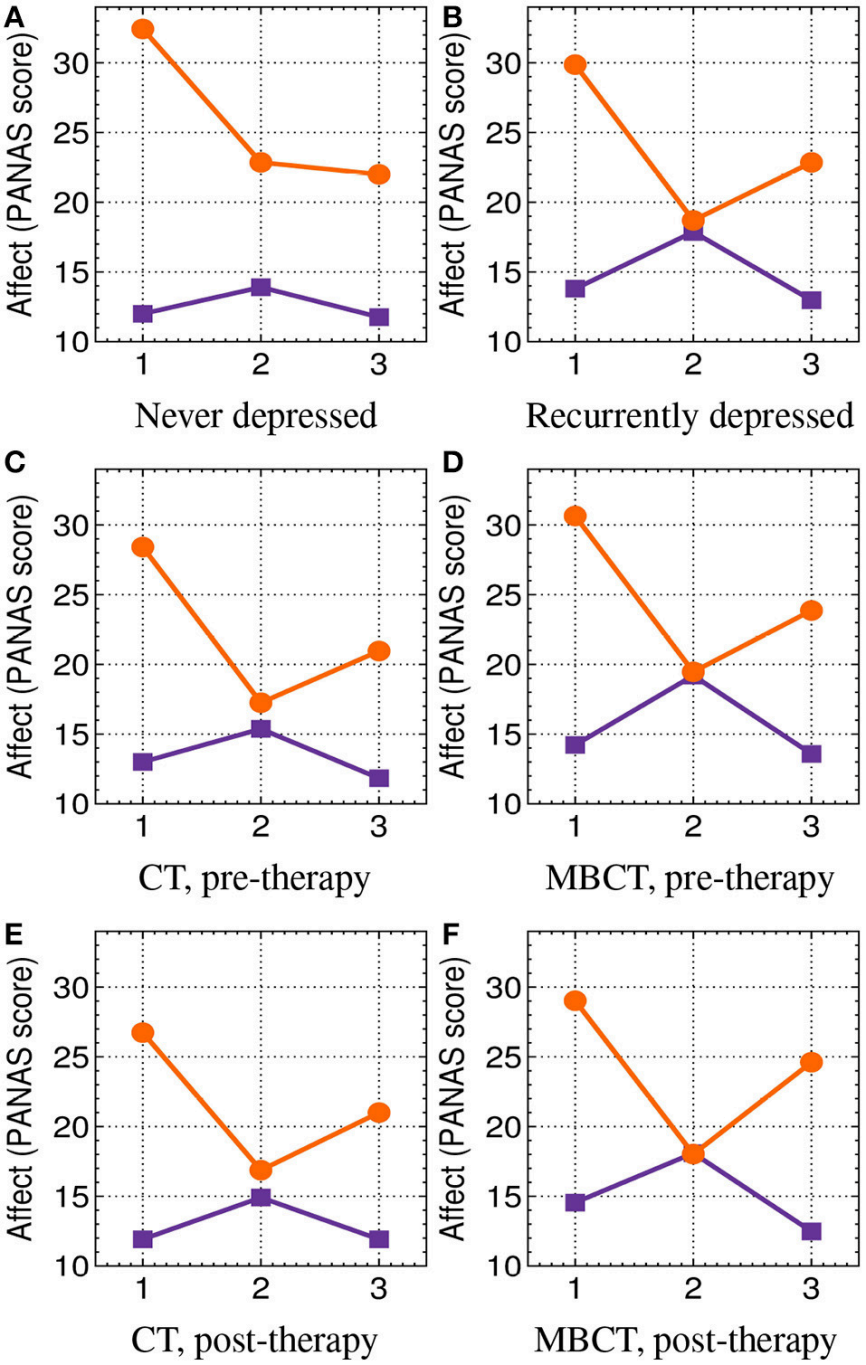
PANAS (section 2.4.3) scores: positive affect (PA, orange circles) and negative affect (NA, purple squares) at three time points: 1, at the beginning of the experimental session; 2, after the mood and rumination induction (section 2.4.4); and 3, after the passive mindfulness meditation task (section 2.4.6). See also Table 3. **(A)** Never depressed. **(B)** Recurrently depressed. **(C)** CT, pre-therapy. **(D)** MBCT, pre-therapy. **(E)** CT, post-therapy. **(F)** MBCT, post-therapy.

The mood & rumination induction (section 2.4.4) was very effective in changing participants' mood. The obtained PANAS scores are presented in Figure 3 and in Table 3.

**Table 3:** PANAS (section 2.4.3) scores: positive affect (PA) and negative affect (NA) at the beginning of the experimental session (baseline), and the corresponding shifts (dPA and dNA) after the mood & rumination (M&R) induction (section 2.4.4), and after the passive mindfulness meditation task (section 2.4.6).

**(Ther- apy) group**	**Exp.ses-sion**	***N***	**Before M&R ind**.		**After M&R induction**				**After mindfulness meditation**			
			**PA**	**NA**	**dPA**	**dNA**			**dPA**	**dNA**
			***M* ± *SD***	***M* ± *SD***	***M* ± *SD***	***M* ± *SD***	***T*^2^**	***p***	***M* ± *SD***	***M* ± *SD***	***T*^2^**	***p***
Non-depr.		21	32.4 ± 6.7	12.0 ± 3.2	−9.6 ± 5.8	1.9 ± 4.0	56.4	0	−0.9 ± 5.2	−2.1 ± 2.9	14.9	0.04^*^
Rec. depr.	Pre	69	29.9 ± 6.0	13.8 ± 4.2	−11.2 ± 6.2	4.1 ± 6.5	222.3	0	4.2 ± 6.9	−4.9 ± 7.5	40.3	0^*^
Rec. depr.	Post	67	28.2 ± 8.0	13.6 ± 4.0	−10.6 ± 7.0	3.4 ± 6.2	163.9	0	5.7 ± 6.9	−4.7 ± 6.2	53.6	0^*^
CT	Pre	24	28.4 ± 4.7	13.0 ± 2.8	−11.2 ± 5.6	2.4 ± 3.6	97.3	0	3.7 ± 6.9	−3.5 ± 3.6	24.6	0.00^*^
CT	Post	23	26.7 ± 7.1	11.9 ± 2.2	-9.9 ± 6.5	3.0 ± 5.2	53.2	0	4.1 ± 6.1	−3.0 ± 5.0	10.8	0.12
MBCT	Pre	45	30.6 ± 6.6	14.2 ± 4.8	−11.2 ± 6.6	5.0 ± 7.4	128.9	0	4.4 ± 6.9	−5.6 ± 8.9	26.1	0^*^
MBCT	Post	44	29.0 ± 8.5	14.5 ± 4.5	−11.0 ± 7.3	3.5 ± 6.7	110.6	0	6.6 ± 7.2	−5.6 ± 6.6	45.2	0^*^

*“Non-depr.” denotes the group of never-depressed students, and “Rec. depr.” is the pooled sample of both therapy groups; “Pre” and “Post” denote the corresponding experimental sessions (pre- and post-therapy); N is the number of participants in the respective therapy group, M is the mean value of the respective affect score or score difference, SD is the standard deviation, and T^2^ and p are the results of Hotelling's T^2^-test checking whether [dPA, dNA] ≠ [0, 0]. All p-values have been corrected (Bonferroni) for multiple (eight) comparisons*;

0.00* and

0.04* denote significant p-values (p < 0.05), and

0* *denotes highly significant p-values (p < 0.0005). A graphical representation of these results is displayed in Figure 3*.

### 3.3. Grand average ERPs

**Figure 4:**
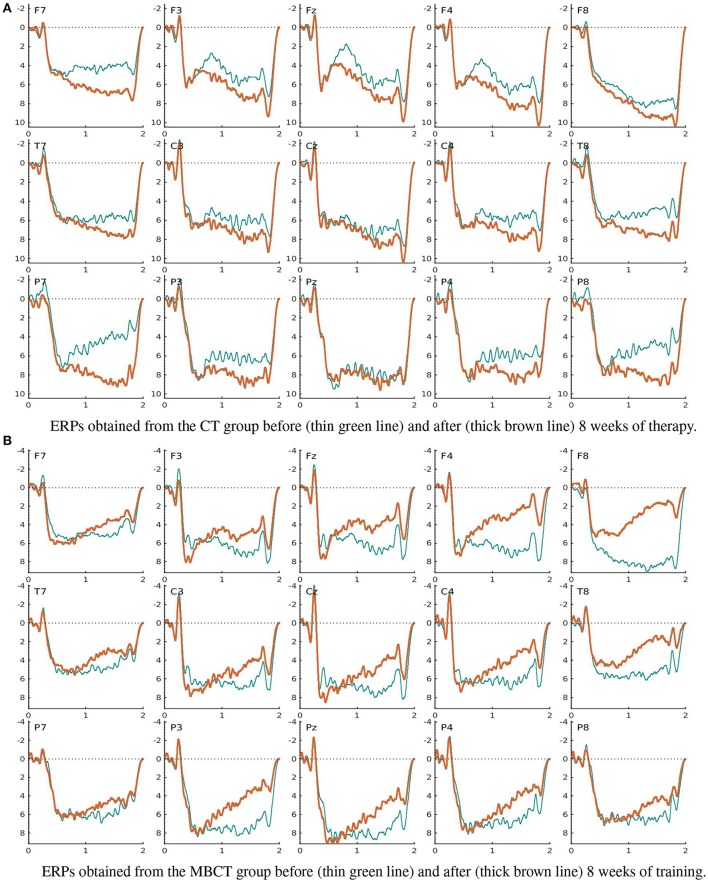
Grand average ERPs obtained in the passive mindfulness meditation task (section 2.4.6). **(A)** ERPs obtained from the CT group before (thin green line) and after (thick brown line) 8 weeks of therapy. **(B)** ERPs obtained from the MBCT group before (thin green line) and after (thick brown line) 8 weeks of training.

**Figure 5:**
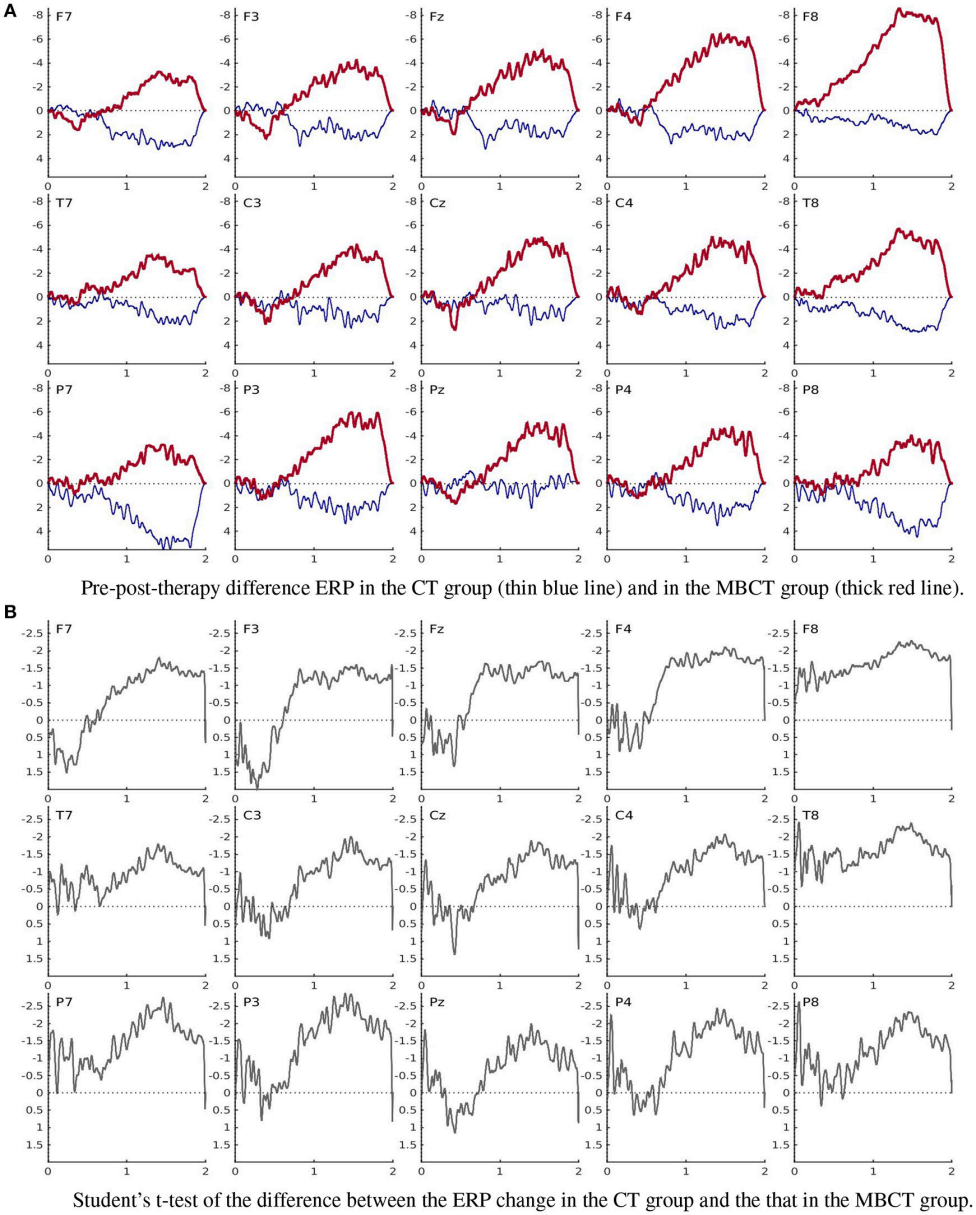
Pre-post-therapy change in the ERPs obtained in the passive mindfulness meditation task (section 2.4.6). **(A)** Pre-post-therapy difference ERP in the CT group (thin blue line) and in the MBCT group (thick red line). **(B)** Student's *t*-test of the difference between the ERP change in the CT group and the that in the MBCT group.

The grand average ERPs obtained from the matched subsamples (Figure 2) with a cutoff frequency *f*_*c*_ = 15 Hz and artifact rejection parameter *C*_σ_ = 4 (section 2.8.3) in the passive mindfulness meditation task (section 2.4.6) are displayed in Figures 4, 5; those obtained in the active task (section 2.4.7) are presented in Figures provided as Supplemental Data. The significantly different patterns of pre-post-therapy change in the two therapy groups obtained in the passive task were consistent with our previous results (Bostanov et al., 2012)—the LCNV amplitude increased after MBCT but decreased after CT (see also Table 4). The opposite (but not significant) patterns were observed in the active task.

**Table 4:** Pre-post-therapy differences and long-term changes in the full samples (section 2.2).

**Measure**	**ERP task**	**CT Group**				**MBCT Group**				**Both (CT** + **MBCT)**				**Difference**		
		***N***	***M* ± *SD***	**|*t*|**	***p***	***N***	***M* ± *SD***	**|*t*|**	***p***	***N***	***M* ± *SD***	**|*t*|**	***p***	***M***	**|*t*_*d*_|**	***p*_*d*_**
DSt		19	12.89 ± 5.8			41	11.27 ± 5.1			60	11.78 ± 5.4			−1.62	1.1	0.28
DSf		19	10.96 ± 6.4			41	10.58 ± 5.3			60	10.70 ± 5.6			−0.39	0.2	0.81
dDS		19	−1.93 ± 3.7	2.3	0.04	41	−0.69 ± 3.4	1.3	0.20	60	−1.08 ± 3.5	2.4	0.02	1.24	1.3	0.21
dERPi	P	24	0.67 ± 0.5			44	0.40 ± 0.6			68	0.50 ± 0.6			−0.27	1.9	0.07
dERPi	A	22	1.02 ± 0.6			44	0.75 ± 0.8			66	0.84 ± 0.7			−0.27	1.4	0.16
dLCNV	P	24	−1.90 ± 8.4	1.1	0.28	44	2.64 ± 11.4	1.5	0.06					4.55	1.7	0.04^*^
dTMSd	P	23	−0.96 ± 5.5	0.8	0.41	44	2.68 ± 5.8	3.1	0.00	67	1.43 ± 5.9	2.0	0.05	3.64	2.5	0.02
dTMSc	P	23	0.09 ± 4.8	0.1	0.93	44	−0.14 ± 5.6	0.2	0.87	67	−0.06 ± 5.3	0.1	0.93	−0.22	0.2	0.87
dTMSd	A	24	−0.04 ± 7.1	0.0	0.98	44	1.98 ± 5.8	2.3	0.03	68	1.26 ± 6.3	1.7	0.10	2.02	1.3	0.21
dTMSc	A	24	−0.29 ± 5.6	0.3	0.80	44	1.00 ± 5.6	1.2	0.24	68	0.54 ± 5.6	0.8	0.42	1.29	0.9	0.37
dMR	A	21	2.52 ± 9.6	1.2	0.24	38	3.63 ± 8.3	2.7	0.01	59	3.24 ± 8.7	2.8	0.01	1.11	0.5	0.65
dPANAS		24	0.46 ± 10.3	0.2	0.83	44	1.86 ± 12.4	1.0	0.33	68	1.37 ± 11.7	1.0	0.34	1.41	0.5	0.64
dRSQsy		15	−5.53 ± 4.4	4.8	0*	43	−2.93 ± 4.5	4.3	0*	58	−3.60 ± 4.6	6.0	0*	2.60	1.9	0.06
dRSQse		15	−4.80 ± 3.2	5.9	0*	43	−2.21 ± 3.8	3.8	0*	58	−2.88 ± 3.8	5.8	0*	2.59	2.4	0.02
dRSQdi		15	2.93 ± 4.7	2.4	0.03	43	2.14 ± 4.2	3.3	0.00	58	2.34 ± 4.3	4.1	0*	−0.79	0.6	0.54
dMAAS		15	4.40 ± 10.9	1.6	0.14	43	3.65 ± 11.3	2.1	0.04	58	3.84 ± 11.1	2.6	0.01	−0.75	0.2	0.82

Pre-post-therapy differences: dERPi, multivariate difference in individual ERPs (section 2.8.3); dLCNV, univariate amplitude differences in late CNV (section 2.8.2); dTMSd and dTMSc, changes in the state mindfulness sub-scores, TMS-decentering and TMS-curiosity, respectively (section 2.4.3); dMR, changes in the motor response in the active meditation task (section 2.4.7). dPANAS, changes in the sum of the decrease in positive affect and the increase in negative affect (dPA − dNA, section 2.4.3) after the mood & rumination induction (section 2.4.4) reflecting the pre-post-therapy change in participants' reaction to the procedure (section 3.2). Long-term changes: dDS = DSf − DSt, where DSf is the mean DS score during follow-up and DSt is the mean DS score during therapy (section 2.6); dRSQsy, dRSQse, dRSQdi, and dMAAS, changes in symptom-focused rumination, self-focused rumination, distraction, and trait mindfulness, respectively (section 2.3), over a period of more than 60 weeks, from the beginning of therapy till the end of the 1-year follow-up. The passive meditation ERP task (section 2.4.6) is denoted by “P,” the active one (section 2.4.6) by “A.” N is the number of participants in the corresponding sample; M is the mean value of the corresponding difference measure; SD is the standard deviation; and t and p are the results of Student's t-tests checking whether the mean is different from zero; |t_d_| and p_d_ are the results from Student's t-tests checking whether the mean in the CT group is different from the mean in the MBCT group. The p-values are not corrected for multiple comparisons; 0^*^ denotes p < 0.0005, i.e., p-values that remain significant after Bonferroni correction for 2 × 14 = 28 comparisons;

0.04* *is the significant p-value resulting from the planned one-sided t-test of the difference between dLCNV in the CT group and dLCNV in the MBCT group*.

### 3.4. Individual ERP changes

**Figure 6:**
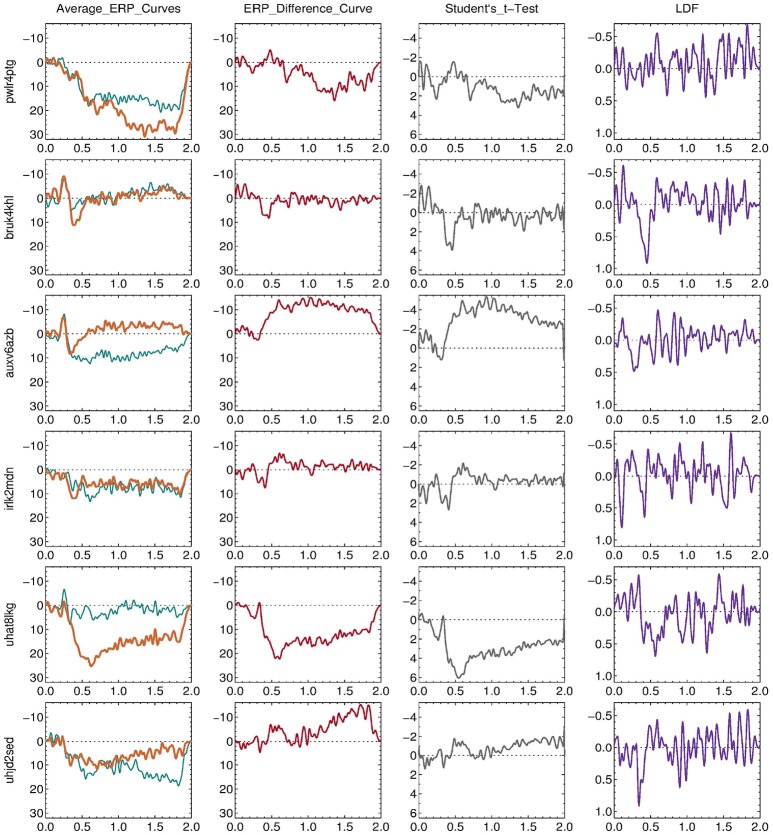
Individual ERP changes of the six MBCT participants (IDs: pwlr4ptg, bruk4khl, auxv6azb, irlk2mdn, uhat8lkg, and uhjd2sed,) whose dERPi and dDS are marked by larger circles in Figure 7. The plots in the first column show the individual average ERPs at Cz obtained in the passive mindfulness meditation task (section 2.4.6) before (thin green line) and after (thick brown line) 8 weeks of training. The plots in the next columns show the corresponding mean difference ERP (second column), Student's *t*-test of the ERP difference (third column), and the linear discriminant function (LDF, section 2.8.3, fourth column).

**Figure 7:**
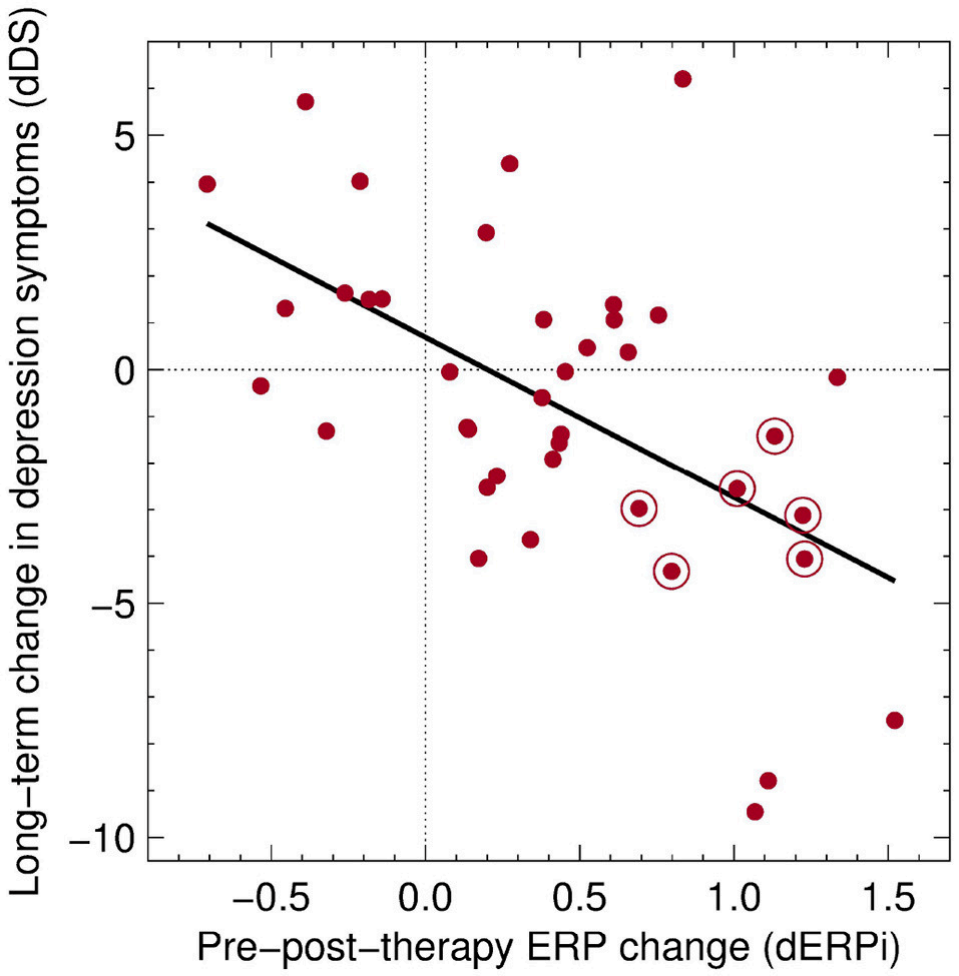
Linear regression model (black line) of the relationship between the passive task dERPi and dDS in the MBCT group (red dots). dERPi was computed for each participant as the logarithm of the t-CWT Mahalanobis distance between the ERP before therapy and the ERP after therapy (section 2.8.3). dDS = DSf − DSt, where DSf is the mean DS score during follow-up and DSt is the mean DS score during therapy (section 2.6). The points marked by larger circles drawn around them belong to the six MBCT participants whose individual ERPs are presented in Figure 6.

Figure 6 shows the individual ERP changes of the six MBCT participants whose dERPi and dDS are marked by larger circles in Figure 7. Their LDFs displayed in the last column of Figure 6 show clearly that the individual ERP change was different in the different participants and did not follow any known (e.g., P300 or CNV) or apparent ERP pattern. The LDF plots also suggest that phase-locked event-related oscillations contributed substantially to the individual ERP changes as reflected by dERPi, since the latter can be represented (section 2.8.3) as the logarithm of the scalar product of the LDF and the individual pre-post-therapy difference (displayed in the second column of Figure 6).

### 3.5. Measures of change

Pre-post therapy differences in ERP measures and various questionnaire scores as well as long-term changes in trait measures of rumination and mindfulness are presented in Table 4. The significant difference in dLCNV in the MBCT group as compared to that in the CT group is consistent with the one we found before (Bostanov et al., 2012). All these tests were performed with both the full and the matched samples (section 2.2). Table 4 shows the results obtained with the full samples. The results obtained with the matched samples were very similar; they are included as Supplemental Data. We also performed (where applicable) ANCOVA tests of between-group differences taking into account baseline levels as covariates. The results from these tests fully confirmed those presented in the last column of Table 4. In particular, the reduction of depressive symptoms (-dDS) in the CT group remained *not* significantly different from that in the MBCT group when the baseline level of depressive symptoms DSt was taken into account as a covariate (*F* = 1.03, *p* = 0.31).

### 3.6. dERPi and dDS in relationship to each other and to other measures

The relationships between some of the obtained measures are presented in Table 5. There was a strong and highly significant relationship between the therapy outcome measure dDS (section 2.6) and the individual multivariate measure dERPi (section 2.8.3) obtained from the MBCT group in the passive task (section 2.4.6). A somewhat weaker and not significant relationship was found also in the CT group. An inspection of the correlations with the baseline DSt and the raw follow-up score DSf showed a more complex picture. In the CT group, DSf was practically independent from dERPi, but dERPi showed some dependence on DSt. In the MBCT group the opposite was true—the relationship of dERPi to DSf was still moderate, while the relationship to DSt was very weak. All these correlations were, however, not significant. Also, none of the (partial) correlations in the MBCT group was significantly different from the corresponding (partial) correlation in the CT group.

**Table 5:** dERPi and dDS in relationship to other measures.

**Measure**	**CT Group**					**MBCT Group**					**Both (CT** + **MBCT)**					**Difference**	
	***N***	***r* **	***p***	***r*_*p*_ **	***p*_*p*_**	***N***	***r* **	***p***	***r*_*p*_ **	***p*_*p*_**	***N***	***r* **	***p***	***r*_*p*_ **	***p*_*p*_**	***p*_*d*_**	***p*_*dp*_**
**dERPi IN RELATIONSHIP TO OTHER MEASURES**																	
dDS	19	−0.40	0.09	−0.52	0.04	40	−0.55	0^*^	−0.65	0^*^	59	−0.52	0^*^	−0.55	0^*^	0.51	0.53
DSt	19	0.20	0.42	0.20	0.46	40	0.13	0.43	0.11	0.53	59	0.18	0.18	0.17	0.21	0.81	0.75
DSf	19	−0.05	0.83	−0.09	0.73	40	−0.23	0.15	−0.31	0.06	59	−0.15	0.24	−0.18	0.19	0.54	0.44
dLCNV	24	−0.05	0.82	−0.00	0.98	44	−0.02	0.87	−0.01	0.94	68	−0.08	0.54	−0.06	0.61	0.93	0.98
dTMSd	23	−0.07	0.76	−0.05	0.85	44	0.23	0.13	0.25	0.12	67	0.07	0.59	0.08	0.51	0.26	0.27
dTMSc	23	0.06	0.77	0.05	0.83	44	−0.01	0.94	0.04	0.80	67	0.01	0.92	0.03	0.80	0.78	0.97
dMR	21	0.25	0.27	0.30	0.22	38	0.26	0.11	0.26	0.13	59	0.22	0.10	0.21	0.12	0.97	0.88
dRSQsy	18	−0.15	0.56	−0.16	0.56	43	−0.02	0.92	0.05	0.78	61	−0.12	0.37	−0.09	0.52	0.67	0.49
dRSQse	18	−0.17	0.51	−0.19	0.50	43	0.16	0.31	0.23	0.16	61	−0.00	0.99	0.00	0.99	0.28	0.16
dRSQdi	18	0.39	0.11	0.43	0.11	43	0.34	0.02	0.38	0.02	61	0.36	0.00	0.37	0.00	0.87	0.85
dMAAS	18	0.33	0.19	0.39	0.15	43	0.23	0.14	0.14	0.39	61	0.25	0.05	0.20	0.13	0.73	0.37
AMPt						40	0.09	0.56	0.03	0.87							
**dDS IN RELATIONSHIP TO OTHER MEASURES**																	
dERPiA	17	−0.15	0.56	−0.00	0.99	40	−0.23	0.14	−0.35	0.04	57	−0.23	0.08	−0.27	0.05	0.79	0.25
dLCNV	19	−0.10	0.69	0.06	0.82	40	−0.16	0.32	−0.11	0.53	59	−0.09	0.51	−0.06	0.65	0.83	0.57
dTMSd	19	−0.27	0.26	−0.39	0.13	40	−0.10	0.53	−0.15	0.38	59	−0.07	0.58	−0.10	0.47	0.56	0.38
dTMSc	19	−0.16	0.51	−0.35	0.19	40	−0.03	0.84	−0.00	0.99	59	−0.08	0.57	−0.04	0.76	0.66	0.23
dMR	17	0.02	0.93	−0.09	0.76	34	−0.16	0.38	−0.15	0.44	51	−0.06	0.67	−0.07	0.61	0.58	0.86
dRSQsy	15	0.10	0.72			40	−0.21	0.20			55	−0.06	0.64			0.35	
dRSQse	15	0.47	0.08			40	−0.32	0.04			55	−0.04	0.79			0.01	
dRSQdi	15	−0.50	0.06			40	−0.17	0.30			55	−0.28	0.04			0.26	
dMAAS	15	−0.36	0.18			40	−0.01	0.94			55	−0.11	0.40			0.27	
AMPt						39	−0.27	0.10									
AMPf						40	−0.17	0.28									

*Pre-post-therapy difference measures: dERPi, individual multivariate ERP difference obtained in the passive meditation task (section 2.4.6), by the t-CWT method, with a cutoff frequency f_c_ = 15 Hz, and artifact rejection parameter C_σ_ = 4 (section 2.8.3); dLCNV, univariate LCNV amplitude difference (section 2.8.2) in the passive task (section 2.4.6); dTMSd and dTMSc, changes in the state mindfulness sub-scores, TMS-decentering and TMS-curiosity, respectively (section 2.4.3) in the passive task (section 2.4.6); dMR, changes in the motor response in the active meditation task (section 2.4.7). Long-term trait changes: dDS = DSf − DSt, where DSf is the mean DS score during follow-up and DSt is the mean DS score during therapy (section 2.6); dRSQsy, dRSQse, dRSQdi, and dMAAS, changes in symptom-focused rumination, self-focused rumination, distraction, and trait mindfulness, respectively (section 2.3), over a period of more than 60 weeks, from the beginning of therapy till the end of the 1-year follow-up. Other measures: AMPt and AMPf, amount of mindfulness practice during the 8 weeks of MBCT and during the 52-week follow-up period, respectively (section 2.7). N is the number of participants in the corresponding sample; r is Pearson's correlation between the corresponding measure and dDS; r_p_ is the corresponding partial correlation with the effects of dBDI, dPAb, and dNAb removed, where dBDI is the pre-post-therapy change residual depressive symptoms as measured by the BDI score, and dPAb and dNAb are the corresponding changes in positive and negative affect in the beginning of the experimental session as measured by the corresponding PANAS subscores (section 2.4.3); p and p_p_ are p-values resulting from Student's t-tests checking whether the r and r_p_ are respectively different from zero; p_d_ and p_dp_ are the p-values resulting from Fisher's z-tests checking whether r or r_p_ in the CT group are different from the corresponding r or r_p_ in the MBCT group. The p-values are not corrected for multiple comparisons*;

0* *denotes p < 0.0005, i.e., p-values that remain significant after Bonferroni correction for 2 × 21 = 42 comparisons (see Table 6)*.

Furthermore, none of the other measures presented in Table 5 showed a significant relationship with dERPi or dDS (after taking into account the Bonferroni correction for multiple tests). Most of the obtained correlations were, however, in the expected direction. Notable exceptions were the following. First, in the MBCT group, dERPi was practically independent from dRSQsy and even positively correlated with dRSQse; this “anomaly” was, however consistent with the negative correlations of dDS with dRSQsy and dRSQse in the same group. Second, while dDS was at least weakly correlated with dLCNV and AMPt, dERPi was virtually independent from both. Third, while dERPi was positively correlated with dMAAS in both groups, dDS showed a moderate negative correlation with it only in the CT group, but not in the MBCT group.

Correlations of AMPt with other difference measures provided further unexpected results. While there was a small positive correlation with dTMSd (*r* = 0.19, *p* = 0.25), the correlation with dTMSc was negative (*r* = −0.15, *p* = 0.36). The correlations with dRSQsy (*r* = 0.27) and dRSQdi (*r* = −0.27) were also in the “wrong direction” (*p* = 0.09). All these correlations were, however, not significant (and the given *p*-values have not been corrected for multiple tests). And, finally, dMAAS, dRSQse, dMR, and dLCNV showed practically no dependence on AMPt (*r* = −0.01, *r* = −0.06, *r* = −0.01, and *r* = 0.03, respectively, *p* > 0.7).

### 3.7. The relationship between dERPi and dDS in the MBCT group

A simple linear regression model for the relationship between dERPi and dDS in the MBCT group is presented in Figure 7. These results as well as the correlations presented in Tables 5, 6 were obtained after excluding the largest negative dDS score, an outlier, more than four standard deviations away from the mean (however, the correlations between dERPi and dDS computed without excluding this outlier were even larger, because this participant had also the smallest dERPi).

**Table 6:** Correlations, *r*, and partial correlations, *r*_p_, between dERPi and dDS (see Table 5) computed in the MBCT group with different cutoff frequencies, *f*_*c*_ = 10, 15, and 20 Hz, different values of the artifact rejection parameter *C*_σ_ = 4, 5, and 9, and using different methods for evaluation of dERPi: with and without t-CWT, and with and without inclusion of the power spectrum (PS, section 2.8.3).

***f*_*c*_**	***C*_σ_**	**PCA**	**t-CWT**	**PS**	***r***	***p***	***r*_p_**	***p*_p_**
10	4	Yes	Yes	No	−0.52	0.0005	−0.59	0.0001
10	4	Yes	No	No	−0.37	0.0174	−0.41	0.0112
10	4	Yes	No	Yes	−0.38	0.0159	−0.42	0.0104
15	4	Yes	Yes	No	−0.55	0.0002	−0.65	0.0000
15	4	Yes	No	No	−0.45	0.0036	−0.50	0.0016
15	4	Yes	No	Yes	−0.45	0.0036	−0.50	0.0016
15	5	Yes	Yes	No	−0.54	0.0003	−0.65	0.0000
15	5	Yes	No	No	−0.44	0.0043	−0.51	0.0013
15	5	Yes	No	Yes	−0.44	0.0043	−0.51	0.0013
15	9	Yes	Yes	No	−0.44	0.0048	−0.49	0.0022
15	9	Yes	No	No	−0.38	0.0146	−0.41	0.0121
15	9	Yes	No	Yes	−0.38	0.0146	−0.41	0.0121
20	4	Yes	Yes	No	−0.55	0.0002	−0.62	0.0000
20	4	Yes	No	No	−0.49	0.0013	−0.53	0.0008
20	4	Yes	No	Yes	−0.48	0.0016	−0.52	0.0010

In order to demonstrate the methodological reliability of the relationship between dERPi and dDS, we present the correlations and partial correlations computed with different sets of dERPi assessment parameters and with and without the t-CWT assessment step (section 2.8.3) in Table 6. The results were very stable across the different sets of parameters except for the setting with artifact rejection turned off (*C*_σ_ = 9). The method including t-CWT provided, generally, somewhat better results (higher correlations) than the method without t-CWT.

The results of the dERPi assessment including the power spectrum (PS, section 2.8.3) are also presented in Table 6. They show virtually *no* contribution of non-phase-locked event-related activity to the relationship between dERPi and dDS.

## 4. Discussion

### 4.1. Measuring mindfulness

In this study, we proposed a psychological measure of mindfulness, dERPi, as an alternative to the established self-report measures (Baer, 2011; Grossman and Van Dam, 2011). dERPi is a multivariate measure of the individual pre-post-therapy change in the ERP obtained in a passive mindfulness meditation task. We found a highly significant relationship between dERPi and dDS, the long-term, persistent change in depression symptoms (assessed weekly by a self-report questionnaire) after therapy. This relationship was expressed as a strong negative correlation (*r* = −0.55) between dERPi and dDS in the MBCT group and an even stronger corresponding partial correlation (*r*_p_ = −0.65) when the effect of pre-post-therapy changes in residual depressive symptoms and mood at the time of measurement was removed. To our knowledge, correlations of this magnitude between predictor and outcome variables are relatively unusual for clinical psychology. The dependence of dDS on the other measures of mindfulness we used in the present study, dMAAS, dTMSd, and dMR, was also substantially weaker than its dependence on dERPi. We provide the following interpretation of this result.

dERPi is a measure of the pre-post-therapy change in the ERP to a test stimulus presented during mindfulness meditation. Although we did not have a no-therapy control group, we can confidently reject the hypothesis that this change is due to a non-specific learning effect caused by the mere repetition of the experimental session, because such non-specific learning cannot explain the strong relationship between dERPi and dDS. By computing partial correlations, we eliminated the possibility that dERPi is (partly) explained by reduction in residual depressive symptoms or general improvement of mood. We also performed the computations with and without the t-CWT step and with different values of the filtering frequency and the parameter controlling the PCA-based preprocessing and obtained similar results, thus, making sure that the obtained effect was not some kind of artifact of the wavelet-based feature extraction or of the specific combination of input parameters of the assessment procedure. Hence, we must assume that dERPi reflects the acquisition of some important knowledge, skills, attitudes or values learned through mindfulness training that are instrumental in the reduction of depressive symptoms. On the other hand, dERPi is a direct measure of an altered brain response to a neutral auditory stimulus during a mindfulness meditation on the breath, that was preceded by a significant mood shift caused by a mood & rumination induction. We see this as converging evidence that dERPi is indeed an accurate measure of the individual neurophysiological correlates of improved mindfulness developed by meditation in the face of a potentially dangerous, depressogenic combination of negative mood and dysphoric rumination.

Further support for the interpretation of dERPi as a measure of mindfulness comes from the correlations between dERPi and the other measures of mindfulness, dMAAS, dTMSd, and dMR, which were all in the expected direction and of the same magnitude as the correlations between these self-report measures and the therapy outcome measure, dDS. These relations were found in the MBCT group. In the CT group, the relationship between dERPi and dDS was smaller and not significant. Furthermore, in the CT group, the raw follow-up score DSf was practically independent from dERPi, but dERPi showed some dependence on the baseline score DSt. In the MBCT group, the opposite was true—the relationship of dERPi to DSf was still moderate, while the relationship to DSt was very weak. Although none of these differences between the groups was significant (possibly, because of the small size of the CT group), the results, nevertheless, provide some tentative evidence suggesting that, in the CT group, dERPi was less related to the decrease in DS. In particular, dERPi does *not* seem to reflect participants' increased ability to apply elaborate cognitive restructuring strategies (learned in the CT group) when confronted with negative thoughts and emotions (like, e.g., refuting the thought “I am a failure” by recollecting past events that stand in contradiction to it). It rather seems that dERPi reflects mindfulness learned largely through *meditation* training (in the MBCT group). It should be noted, however, that Teasdale et al. (2002) demonstrated empirically that *both* MBCT and CT prevent depressive relapse/recurrence by increasing metacognitive awareness as predicted by Teasdale et al. (1995). Hence, even thought dERPi is, by construction, a measure of *meditative* mindfulness, and meditation is *not* learned in CT, it could, nevertheless, be expected to find some smaller but significant relationship between dERPi and dDS in a larger CT sample in a future replication of our experiment.

The validity of the results is also supported by the fact that both interventions were delivered according to standard protocols by experienced therapists, the CT therapists had *no* experience with mindfulness or meditation, and the obtained 1-year relapse/recurrence rates (CT: 36.8%, MBCT: 42.8%) stand in very good agreement with those reported in randomized clinical trials (RCTs) evaluating the efficacy of CT (Bockting et al., 2005) and MBCT (Fjorback et al., 2011; Kuyken et al., 2015).

In the construction of our measure dERPi, we used ERPs in an unusual way. Instead of focusing on group changes in particular, well-known ERP components, we assessed complex individual changes in the whole ERPs. This approach is standard in brain-computer interface (BCI) applications (Blankertz et al., 2004; Bostanov, 2004) and has also been used in neurological diagnostics (Bostanov and Kotchoubey, 2006; Daltrozzo et al., 2009; Steppacher et al., 2013; Kotchoubey, 2015). But is it justified in the measurement of mindfulness? Using this approach makes it impossible to relate our findings to any of the well-established results known from the ERP literature. Nevertheless, we believe that multivariate assessment is not only justified, but also necessary for the ERP measurement of such a multifaceted construct as mindfulness. To explain why we think so, we come back to the fundamental question: What is mindfulness?

Even a very brief review of the Buddhist roots provides a very complex and fairly confusing picture. Starting with the most ancient texts of *Theravada*, the *Pali Suttas*, we find several, partly ambiguous, definitions (Bodhi, 2005, 2011; Ñanamoli and Bodhi, 2009). The later definitions of the *Pali Abhidhamma* are more consistent but somewhat different from those in the *Suttas* (Bodhi, 2000; Dreyfus, 2011; Olendzki, 2011). Both sources, however, define mindfulness twofold—as a basic constituent mental factor and as the seventh factor, “right mindfulness,” of the Noble Eightfold Path, the Buddhist program for spiritual development leading to liberation from suffering. Even as an irreducible mental factor, mindfulness is very subtle and hard to grasp. For instance, it is difficult to understand how it is different from both ordinary sustained conscious awareness and from meditative concentration (Dreyfus, 2011). Moreover, the *Abhidhamma* postulates that mindfulness *always* arises together with 18 other “wholesome” factors (like, e.g., non-craving, non-aversion, equanimity, etc.), i.e., it is objectively inseparable from them and the distinction is possible only through introspection (Bodhi, 2000; Olendzki, 2011). This supports the notion of a whole mode of mind as defined by Teasdale (1999a) and Bishop et al. (2004). As a path factor, mindfulness is defined by the practice described by the Buddha in his discourse on “The four establishments of mindfulness” (Bodhi, 2005; Ñanamoli and Bodhi, 2009).

The twofold concept is further complicated by later developments by *Mahayana* schools that redefine mindfulness as an *innate, universally present* mental factor, whose presence is, however, obscured by other “unwholesome” factors (Olendzki, 2011). Mindfulness as a practice is then redefined as the cultivation of *non-conceptual* awareness that eliminates the unwholesome factors and allows the innate mindfulness and wisdom to grasp the *non-dual* nature of reality (Dunne, 2011).

The contemporary mindfulness-based clinical interventions, MBSR/MBCT were heavily influenced by both *Theravada* and *Mahayana* theory and practice (Kabat-Zinn, 2011). For instance, in MBCT session four, we teach our clients to be mindfully aware of the pleasant, painful, or neutral quality of experience and to mindfully notice and abandon the “unwholesome” tendencies of craving for/clinging to pleasant experience and aversion to/avoidance of unpleasant experience, thus cultivating radical acceptance (Segal et al., 2002). This practice is not very different from the original “second establishment of mindfulness” (Bodhi, 2005; Ñanamoli and Bodhi, 2009), and it is only one of several techniques MBCT participants are taught to apply when challenged by negative emotions and thoughts. Alternatives include: bringing awareness to the physiological manifestation of emotions as bodily sensations (first establishment of mindfulness); focusing on the impermanence of experience; noticing unwholesome, self-deprecating, ruminative thoughts and clearly comprehending their true nature as mere thoughts, not facts, or explicitly seeing the inherent “delusion” of overgeneralizations, black-and-white judgments, catastrophic thinking, etc.; or simply abiding in non-conceptual awareness, trusting that the innate wisdom manifests itself spontaneously and naturally transforms suffering (*Mahayana* practice). In the later sessions of a MBCT course, participants are invited to chose from the variety of techniques and practices they have learned and to see which fit best their personal needs and abilities (Segal et al., 2002).

All said above should have demonstrated that, in both traditional, spiritual, and contemporary, clinical, contexts, mindfulness is defined by the intricate interplay of many factors including general values, explicit intellectual knowledge, implicit attentional and other cognitive skills, a specific “spirit” or attitude toward experience, and a variety of training practices by which all of the above are developed. Furthermore, different persons may focus predominantly on certain aspect(s) of mindfulness and prefer the corresponding training practice(s). Hence, it should be expected that it would manifest itself as different neurophysiological processes, reflected by different multivariate ERP patterns rather than by single ERP components. For instance, mindfulness of bodily sensations should be represented by different ERP patterns than mindfulness of mental processes. Thus, multivariate ERP assessment seems mandatory in the proposed psychophysiological measurement of mindfulness.

### 4.2. Measuring concentration

The initial goals of the present study were to improve the mood & rumination part of our mindfulness paradigm, to replicate the dLCNV effect (Bostanov et al., 2012), and to test whether dLCNV predicts therapy outcome. The obtained PANAS scores showed that our new mood & rumination induction procedure was indeed more effective than the previous one in reducing positive affect and increasing negative affect. We could also replicate for a second time the dLCNV effect we found in our previous studies, this time with an active control group, CT. This suggests that this is a stable pattern that distinguishes meditation from other kinds of cognitive and behavioral training. The relationship between dLCNV as a measure of meditative attention and dDS as (an inverse) measure of long-term improvement was, however, very weak (*r*_p_ = −0.1). Moreover, dLCNV was virtually independent from our new measure of mindfulness, dERPi. Why was this so? We suggest the following explanation.

In the practice of meditation, there is a subtle, but important distinction between sustained mindful attention vs. meditative concentration (*sati* vs. *samadhi*). Concentration, characterized by one-pointedness of attention, can focus most (or even all) attentional resources on the perception of a single object (Bodhi, 2000; Dreyfus, 2011). According to modern *Theravada* tradition (Tejaniya, 2016) and MBSR/MBCT (Kabat-Zinn, 1990; Segal et al., 2002), mindfulness can be cultivated and practiced in everyday life *without* meditative concentration. Meditation (of any kind), however, *cannot* be practiced without at least some concentration. When meditation is practiced with strong emphasis on concentration, this has the effect that all kinds of thought processes, including dysphoric rumination and worry, are temporarily suppressed, which leads to the experience of calm, serenity, body relaxation, joy, and rapture (Bodhi, 2000, 2005; Ñanamoli and Bodhi, 2009). Although meditation beginners cannot attain the states of mind described in the *Suttas*, they still experience some of the pleasant effects of concentration. MBCT participants are explicitly warned against *clinging* to such experiences (Segal et al., 2002) for two reasons. First, clinging to the pleasant effects of concentration inevitably leads to frustration, because it is not possible to remain in such states for a long time. Frustration, when not processed with mindfulness, may in turn facilitate depressive relapse. Second, while there is evidence that metacognitive awareness supported by mindful attention facilitates emotional processing and predicts positive therapy outcome (Teasdale et al., 2002), there is no evidence that concentration *per se* is of any long-term therapeutic value. This is consistent with a central axiom of Buddhism stating that liberation from suffering is caused by insight facilitated by mindfulness, and not by concentration *alone* (Teasdale and Chaskalson, 2011a,b). Meditative concentration is, however, required for mindfulness meditation and is actively cultivated in MBCT (Segal et al., 2002, p. 93). Hence, it could be expected that it should correlate with therapy outcome like mindfulness does. This hypothesis had, however, never been tested empirically, and this test was, one of the goals of the present study.

As already mentioned in the Introduction, according to both well-established results from the CNV literature (Tecce, 1972; Tecce and Cattanach, 1993; Travis et al., 2000, 2002; Brunia and van Boxtel, 2001; Cahn and Polich, 2006) and our own previous (Bostanov et al., 2012) and present findings, we see dLCNV as a measure of increased meditative concentration that makes attentional resources available for a more focused and intense perception of any object, including the probe stimulus in our passive mindfulness paradigm. Our present results suggest, however, that concentration ability has little influence on the therapeutic effect of mindfulness. In other words, it may be relatively unimportant how well MBCT participants actually manage to focus their attention on the breath. It rather seems that the therapeutically critical learning occurs while they (mindfully) try to do so, again and again.

### 4.3. Amount of mindfulness practice

One unexpected result of our study was the very weak or zero dependence of the therapy outcome and the various measures of learned mindfulness on the amount of mindfulness practice during the MBCT course, AMPt. While dDS still showed some weak (but not significant) dependence on AMPt (*r* = −0.27), both dERPi and dLCNV were virtually independent from it. The same was true for the self-report measures dMAAS, and dMR; only dTMSd showed some weak (not significant) relationship to AMPt (*r* = 0.19). We suspect that some kind of ceiling effect might have been responsible for these results. All but one MBCT participant (excluded as outlier) reported at least 15 to 30 min of practice at least three or four days a week (in addition to the weekly meditation exercises during the group sessions). This is a lot of practice and all we can say is that, probably, those who tried even harder did not necessarily achieve more in terms of increased mindfulness or reduction of depressive symptoms. Or, even if they actually did benefit more in terms of general mental health and well-being, this could not be measured well by dDS, because of the natural floor effect caused by the constraint DSf ≥ 0 (i.e., DS measures only depression, and not happiness in the absence of depressive symptoms).

### 4.4. Self-reported rumination

The rumination change scores, dRSQsy, dRSQse, and dRSQdi were the only measures that remained significant after the correction for multiple comparisons. As expected, there was a long-term decrease in symptom-focused and self-focused rumination (dRSQsy < 0, dRSQse < 0), and an increase in distraction (dRSQdi > 0) after therapy in both groups, MBCT and CT. The positive dRSQdi can be easily explained by the fact that all RSQ distraction items actually describe cognitive or behavioral strategies for coping with depressed mood, all of which are learned in both MBCT and CT (Nolen-Hoeksema, 1991; Nolen-Hoeksema and Morrow, 1991; Segal et al., 2002). In the MBCT group, however, dRSQsy and dRSQse showed an unexpected, negative relationship with dDS, consistant with an, also anomalous, positive relationship with dERPi. This means that more rumination after therapy was associated with a better outcome in the MBCT group. In contrast, the corresponding correlations in the CT group had the expected sign (positive for dDS an negative for dERPi). This strange effect might be explained by the fact that the RSQ, like any self-report measure, can only assess the *subjectively perceived* frequency of ruminative responses. MBCT participants whose mindfulness was better developed might have noticed more ruminative patterns in their cognitive style compared to less mindful participants who, objectively, ruminated equally or more frequently than the latter, but were, subjectively, less aware of that.

### 4.5. The active ERP task

There are two possible reasons why neither the dLCNV effect nor the relationship between dDS and dERPi in the active ERP task was significant. First, the effect of the mood & rumination induction may be crucial for the mindfulness meditation paradigm. The obtained PANAS scores showed, however, that this effect was largely neutralized after the passive task (i.e., practically, not present during the following active task). Second, the active task is by far not as close to real mindfulness meditation as the passive one, and the required motor responses might have prevented the participants from attaining a mindful mode of mind.

### 4.6. Limitations

The proposed measure dERPi was designed by construction to quantify only *changes* in mindfulness, e.g., after mindfulness training, and, obviously, cannot be used for the assessment of baseline or trait mindfulness. Furthermore, our design suggests that this measure might only be valid in the population of recurrently depressed people with no previous experience with any kind of meditaion. On the other hand, our mood & rumination procedure was as effective with young, never-depressed students as it was with recurrently depressed participants, which suggests that dERPi might be applicable with other populations as well (possibly, after proper adjustment of the mood & rumination procedure to the target group, e.g., worry induction for people suffering from anxiety, or craving induction by cue exposure for recovered addicts, etc.).

Another limitation of our study was posed by sample size: the CT sample was too small to lend statistical significance to the relationship between dERPi and dDS in that group, and both groups were too small to tell whether this relationship was really stronger in the MBCT group than it was in the CT group. It should be expected that a replication with a larger CT sample should yield some smaller but significant relationship between dERPi and dDS in agreement with the result found by Teasdale et al. (2002) that both mindfulness learned predominantly through mediation (in MBCT) and metacognitive awareness learned through cognitive, behavioral and educational techniques (in CT) predict therapy outcome. And, needless to say, several other correlations that were not significant in our samples may turn significant after replication with larger samples.

The recruitment problems resulting in insufficient number of participant also caused another methodological weakness, namely, the absence of a clean baseline for dDS, the long-term reduction of depression symptoms. Some participants were included in the last week before the start of their therapy, and, for this reason, we had to use DSt, the mean depression score *during* the therapy course as a baseline for dDS. DSt might reflect not only the baseline residual depressiveness of the participants, but also their reaction to the therapy. We attempted a partial remedy of the situation by additionally computing and analyzing the correlations with DSt and DSf, but a future replication should address this issue properly by performing a correct baseline assessment *before* therapy.

Yet another limitation of our study (pointed out by an anonymous reviewer) is posed by the threefold role of one of us (VB) as a MBCT therapist, principal investigator, and first author of the current article, which might have biased the choice of assessment methods in a way that makes it more probable or easier to find stronger effects (e.g., a stronger dDS-dERPi relationship) in the MBCT group.

Without a clear *rejection* of the hypothesis that the correlations between dERPi and dDS, computed with a proper DS baseline, are equal in the two therapy groups, the possibility remains that dERPi is affected by placebo effects or by some newly learned skills, attitudes, values, etc. that are different from mindfulness and are acquired in both MBCT and CT. Thus, a replication with a sufficient sample size and a correct DS baseline would substantially corroborate the validity of dERPi as a measure of mindfulness.

## 5. Conclusion

In the present study, we found an unusually strong relationship between the individual pre-post-therapy change in the electrophysiological brain response during mindfulness meditation and the long-term therapy outcome. While it might be tempting to claim that we have found a way to measure mindfulness directly from people's heads, we would rather prefer to see our results as very promising, but yet not conclusive. Apart from methodological issues that can be easily addressed in future studies, a replication by a *different* research team would provide crucial confirmation of the validity and the reliability of the proposed psychophysiological method. Thus, the dERPi measure could become an important component of a “mindfulness test battery” together with self-report questionnaires and other newly developed instruments (Baer, 2016).

## Author contributions

VB designed the study, managed the whole project, designed and implemented all experimental procedures, delivered MBCT, analyzed the data, and wrote this manuscript. LO was the recruitment manager and together with RB conducted all personal diagnostics and all experimental sessions. MH supervised the whole study as an expert in clinical psychology, CT, and depression. BK supervised the whole study as an expert in neuroscience, EEG, and ERP.

### Conflict of interest statement

The authors declare that the research was conducted in the absence of any commercial or financial relationships that could be construed as a potential conflict of interest.

## Supplementary material

Supplementary data is included in the ZIP archive. Two supplementary figures are included in Supplementary Presentation 1. The Supplementary Material for this article can be found online at: https://www.frontiersin.org/articles/10.3389/fnhum.2018.00249/full#supplementary-material

## References

[B1] APA (2000). Diagnostic and Statistical Manual of Mental Disorders – DSM-IV-TR, 4th Edn., Text Revision). Washington, DC: American Psychiatric Association.

[B2] Baer R. (2016). Assessment of mindfulness and closely related constructs: introduction to the special issue. Psychol. Assess. 28, 787–790. 10.1037/pas0000309 27078184

[B3] Baer R. A. (2011). Measuring mindfulness. Contemp Buddhism 12, 241–261. 10.1080/14639947.2011.564842

[B4] Baranov-Krylov I. N. Kanunikov I. E. Shuvaev V. T. Berlov D. N. Kavshbaya N. A. (2003). Assessment of the state of activation of the cortical zones in humans during visual attention and selection. Neurosci. Behav. Physiol. 33, 439–445. 10.1023/A:1023455032072 12921174

[B5] Beck A. T. Steer R. A. Ball R. Ranieri W. (1996). Comparison of Beck Depression Inventories -IA and -II in psychiatric outpatients. J. Pers. Assess. 67, 588–597. 10.1207/s15327752jpa6703_13 8991972

[B6] Bishop S. R. Lau M. Shapiro S. Carlson L. Anderson N. D. Carmody J. (2004). Mindfulness: a proposed operational definition. Clin. Psychol. Sci. Pract. 11, 230–241. 10.1093/clipsy.bph077

[B7] Blankertz B. Müller K. R. Curio G. Vaughan T. M. Schalk G. Wolpaw J. R.. (2004). The BCI competition 2003: progress and perspectives in detection and discrimination of EEG single trials. IEEE Trans. Biomed. Eng. 51, 1044–1051. 10.1109/TBME.2004.826692 15188876

[B8] Bockting C. L. Schene A. H. Spinhoven P. Koeter M. W. Wouters L. F. Huyser J.. (2005). Preventing relapse/recurrence in recurrent depression with cognitive therapy: A randomized controlled trial. J. Consult. Clin. Psychol. 73, 647–657. 10.1037/0022-006X.73.4.647 16173852

[B9] Bodhi B. (ed.). (2000). A Comprehensive Manual of Abhidhamma. Onalaska, WI: Pariyatti Publishing.

[B10] Bodhi B. (2005). In the Buddha's Words – An Anthology of Discourses From the Pali Canon. Boston, MA: Wisdom Publications.

[B11] Bodhi B. (2011). What does mindfulness really mean? A canonical perspective. Contemp Buddhism 12, 19–39. 10.1080/14639947.2011.564813

[B12] Bostanov V. (2004). BCI Competition 2003 - data sets Ib and IIb: feature extraction from event-related brain potentials with the continuous wavelet transform and the t-value scalogram. IEEE Trans. Biomed. Eng. 51, 1057–1061. 10.1109/TBME.2004.826702 15188878

[B13] Bostanov V. (2015a). Multivariate assessment of event-related potentials with the t-CWT method. BMC Neurosci. 16:73 10.1186/s12868-015-0185-z 26541673 PMC4635610

[B14] Bostanov V. (2015b). t-CWT 2.01: A Software Implementation of the t-CWT Method for Multivariate Assessment of Event-Related Potentials. Free and open source code for MATLAB and GNU Octave. Available online at: http://bioinformatics.org/tcwt/ or http://tcwt.de/

[B15] Bostanov V. (2015c). t-CWT 2.01 Software Documentation. Available online at: http://bioinformatics.org/tcwt/ or http://tcwt.de/

[B16] Bostanov V. (2017a). t-CWT 3.00 (Mindfulness): A Software Implementation of the t-CWT Method for Multivariate Assessment of Event-Related Potentials. Free and open source code for MATLAB and GNU Octave. Available online at: http://bioinformatics.org/tcwt/ or http://tcwt.de/

[B17] Bostanov V. (2017b). t-CWT 3.00 (Mindfulness) Software Documentation. Available online at: http://bioinformatics.org/tcwt/ or http://tcwt.de/

[B18] Bostanov V. Keune P. M. Kotchoubey B. Hautzinger M. (2012). Event-related brain potentials reflect increased concentration ability after mindfulness-based cognitive therapy for depression: a randomized clinical trial. Psychiatry Res. 199, 174–180. 10.1016/j.psychres.2012.05.031 22771173

[B19] Bostanov V. Kotchoubey B. (2006). The t-CWT: a new ERP detection and quantification method based on the continuous wavelet transform and Student's t-statistics. Clin. Neurophysiol. 117, 2627–2644. 10.1016/j.clinph.2006.08.012 17030012

[B20] Broderick P. C. (2005). Mindfulness and coping with dysphoric mood: contrasts with rumination and distraction. Cogn. Ther. Res. 29, 501–510. 10.1007/s10608-005-3888-0

[B21] Brown K. W. Ryan R. M. (2003). The benefits of being present: mindfulness and its role in psychological wellbeing. J. Pers. Soc. Psychol. 84, 822–848. 10.1037/0022-3514.84.4.822 12703651

[B22] Brunia C. H. M. van Boxtel G. J. M. (2001). Wait and see. Int. J. Psychophysiol. 43, 59–75. 10.1016/S0167-8760(01)00179-9 11742685

[B23] Burg J. M. Michalak J. (2011). The healthy quality of mindful breathing: associations with rumination and depression. Cogn. Ther. Res. 35, 179–185. 10.1007/s10608-010-9343-x

[B24] Cahn B. R. Polich J. (2006). Meditation states and traits: EEG, ERP, and neuroimaging studies. Psychol. Bull. 132, 180–211. 10.1037/0033-2909.132.2.180 16536641

[B25] Daltrozzo J. Wioland N. Mutschler V. Lutun P. Calon B. Meyer A.. (2009). Cortical information processing in coma. Cogn. Behav. Neurol. 22, 53–62. 10.1097/WNN.0b013e318192ccc8 19372771

[B26] Donaldson C. Lam D. (2004). Rumination, mood and social problem-solving in major depression. Psychol. Med. 34, 1309–1318. 10.1017/S0033291704001904 15697057

[B27] Dreyfus G. (2011). Is mindfulness present-centred and non-judgmental? A discussion of the cognitive dimensions of mindfulness. Contemp Buddhism 12, 41–54. 10.1080/14639947.2011.564815

[B28] Dunne J. (2011). Toward an understanding of non-dual mindfulness. Contemp Buddhism 12, 71–88. 10.1080/14639947.2011.564820

[B29] Ensel W. (1986). Measuring depression. The CES-D scale, in Social Support, Life Events, and Depression, eds Lin N. Dean A. Ensel W. (Orlando FL: Academic Press), 51–70.

[B30] First M. B. Spitzer R. L. Gibbon M. Williams J. (1996). Structured Clinical Interview for DSM-IV Axis I Disorders. New York, NY: Biometrics Research Department, New York State Psychiatric Institute.

[B31] Fjorback L. O. Arendt M. Ornbøl E. Fink P. Walach H. (2011). Mindfulness-based stress reduction and mindfulness-based cognitive therapy – a systematic review of randomized controlled trials. Acta Psychiat. Scand. 124, 102–119. 10.1111/j.1600-0447.2011.01704.x 21534932

[B32] Grossman P. Van Dam N. T. (2011). Mindfulness, by any other name: trials and tribulations of sati in western psychology and science. Contemp Buddhism 12, 219–239. 10.1080/14639947.2011.564841

[B33] Hautzinger M. (2010). Akute Depression. Göttingen: BeltzPVU.

[B34] Hautzinger M. (2013). Kognitive Verhaltenstherapie bei Depressionen, 7th Edn Weinheim: Beltz.

[B35] Hautzinger M. Bailer M. Hofmeister D. Keller F. (2012). ADS. Allgemeine Depressionsskala, 2nd Edn Göttingen: Hogrefe.

[B36] Hautzinger M. Keller F. Kühner C. (2007). Deutsche Adaptation des Beck Depressions Inventar BDI-II. Frankfurt: Harcourt Test Services.

[B37] Hayes S. C. Strosahl K. D. Wilson K. G. (1999). Acceptance and Commitment Therapy: An Experiential Approach to Behavior Change. New York, NY: Guilford.

[B38] Hofmann S. G. Sawyer A. T. Witt A. A. Oh D. (2010). The effect of mindfulness-based therapy on anxiety and depression: a meta-analytic review. J. Consult. Clin. Psychol. 78:169 10.1037/a0018555 20350028 PMC2848393

[B39] Ivanovski B. Malhi G. S. (2007). The psychological and neurophysiological concomitants of mindfulness forms of meditation. Acta Neuropsychiatr. 19, 76–91. 10.1111/j.1601-5215.2007.00175.x 26952819

[B40] Jarrett R. B. Kraft D. Doyle J. Foster B. M. Eaves G. G. Silver P. C. (2001). Preventing recurrent depression using cognitive therapy with and without a continuation phase. Arch. Gen. Psychiatry 58, 381–388. 10.1001/archpsyc.58.4.381 11296099 PMC1307495

[B41] Kabat-Zinn J. (1990). Full Catastrophe Living: The Program of the Stress Reduction Clinic at the University of Massachusetts Medical Center. New York, NY: Hyperion.

[B42] Kabat-Zinn J. (2011). Some reflections on the origins of mbsr, skillful means, and the trouble with maps. Contemp Buddhism 12, 281–306. 10.1080/14639947.2011.564844

[B43] Klados M. A. Papadelis C. Braun C. Bamidis P. D. (2011). REG-ICA: a hybrid methodology combining blind source separation and regression techniques for the rejection of ocular artifacts. Biomed. Signal Proces. 6, 291–300. 10.1016/j.bspc.2011.02.001

[B44] Kotchoubey B. (2015). Event-related potentials in disorders of consciousness, in Clinical Neurophysiology in Disorders of Consciousness, eds Rossetti A. O. Laureys S. (Vienna: Springer), 107–123.

[B45] Krohne H. W. Egloff B. Kohlmann C. W. Tausch A. (1996). Untersuchungen mit einer deutschen Version der ”Positive and Negative Affect Schedule” (PANAS). Diagnostica 42, 139–156.

[B46] Kühner C. Huffziger S. Nolen-Hoeksema S. (2007). Der Response Styles Questionnaire – Deutsche Version (RSQ-D). Göttingen: Hogrefe.

[B47] Kuyken W. Hayes R. Barrett B. Byng R. Dalgleish T. Kessler D. (2015). Effectiveness and cost-effectiveness of mindfulness-based cognitive therapy compared with maintenance antidepressant treatment in the prevention of depressive relapse or recurrence (PREVENT): a randomised controlled trial. Lancet 386, 63–73. 10.1016/S0140-6736(14)62222-4 25907157

[B48] Lau M. A. Bishop S. R. Segal Z. V. Buis T. Anderson N. D. Carlson L.. (2006). The Toronto mindfulness scale: development and validation. J. Clin. Psychol. 62, 1445–1467. 10.1002/jclp.20326 17019673

[B49] Lyubomirsky S. Nolen-Hoeksema S. (1993). Self-perpetuating properties of dysphoric rumination. J. Pers. Soc. Psychol. 65, 339–349. 10.1037/0022-3514.65.2.339 8366423

[B50] Michalak J. Heidenreich T. Ströhle G. Nachtigall C. (2008). Die deutsche version der mindful attention and awareness scale (MAAS): psychometrische Befunde zu einem Achtsamkeitsfragebogen. Z. Klin. Psychol. Psychother. 37, 200–208. 10.1026/1616-3443.37.3.200

[B51] Ñanamoli B. Bodhi B. (2009). The Middle Length Discourses of the Buddha. A Translation of the Majjhima Nikaya, 4th Edn Boston, MA: Wisdom Publications.

[B52] Nolen-Hoeksema S. (1987). Sex differences in unipolar depression: evidence and theory. Psychol. Bull. 101, 259–282. 10.1037/0033-2909.101.2.259 3562707

[B53] Nolen-Hoeksema S. (1991). Responses to depression and their effects on the duration of depressive episodes. J. Abnorm. Psychol. 100, 569–582. 10.1037/0021-843X.100.4.569 1757671

[B54] Nolen-Hoeksema S. Morrow J. (1991). A prospective study of depression and posttraumatic stress symptoms after a natural disaster: the 1989 loma prieta earthquake. J. Pers. Soc. Psychol. 61, 115–121. 10.1037/0022-3514.61.1.115 1890582

[B55] Oldfield R. C. (1971). The assessment and analysis of handedness: the edinburgh inventory. Neuropsychologia 9, 97–113. 10.1016/0028-3932(71)90067-4 5146491

[B56] Olendzki A. (2011). The construction of mindfulness. Contemp Buddhism 12, 55–70. 10.1080/14639947.2011.564817

[B57] Pfurtscheller G. Da Silva F. L. (1999). Event-related EEG/MEG synchronization and desynchronization: basic principles. Clin. Neurophysiol. 110, 1842–1857. 10.1016/S1388-2457(99)00141-8 10576479

[B58] Polich J. (1987). Comparison of P300 from a passive tone sequence paradigm and an active discrimination task. Psychophysiology 24, 312–320. 10.1111/j.1469-8986.1987.tb01859.x 3575593

[B59] Pribram K. H. McGuinness D. (1992). Attention and para-attentional processing. Event-related brain potentials as tests of a model. Ann. N.Y. Acad. Sci. 658, 65–92. 10.1111/j.1749-6632.1992.tb22839.x 1497264

[B60] Radloff L. (1977). The CES-D scale. A self-report depression scale for research in the general population. Appl. Psych. Meas. 1, 385–401. 10.1177/014662167700100306

[B61] Radloff L. Locke B. (2000). Center for epidemiologic studies depression scale, in Handbook of Psychiatric Measures, eds Rush A. J. First M. B. Blacker D. (Washington, DC: American Psychiatric Publication), 506–508.

[B62] Rencher A. C. (1998). Multivariate Statistical Inference and Applications. New York, NY: John Wiley & Sons.

[B63] Risch A. K. Stangier U. Heidenreich T. Hautzinger M. (2012). Kognitive Erhaltungstherapie bei Rezidivierender Depression. Rückfälle Verhindern, Psychische Gesundheit Erhalten. Berlin: Springer.

[B64] Rush A. J. Trivedi M. H. Ibrahim H. M. Carmody T. J. Arnow B. Klein D. N.. (2003). The 16-item quick inventory of depressive symptomatology (QIDS), clinician rating (QIDS-C), and self-report (QIDS-SR): a psychometric evaluation in patients with chronic major depression. Biol. Psychiat. 54, 573–583. 10.1016/S0006-3223(02)01866-8 12946886

[B65] Segal Z. V. Williams J. M. Teasdale J. D. (2002). Mindfulness-based Cognitive Therapy for Depression: A New Approach to Preventing Relapse. New York, NY: Guilford Press.

[B66] Segal Z. V. Williams J. M. G. Teasdale J. D. (2008). Die Achtsamkeitsbasierte Kognitive Therapie der Depression: Ein Neuer Ansatz zur Rückfallprävention. Tübingen: DGVT-Verlag.

[B67] Steppacher I. Eickhoff S. Jordanov T. Kaps M. Witzke W. Kissler J. (2013). N400 predicts recovery from disorders of consciousness. Ann. Neurol. 73, 594–602. 10.1002/ana.23835 23443907

[B68] Teasdale J. D. (1999a). Emotional processing, three modes of mind and the prevention of relapse in depression. Behav. Res. Ther. 37(Suppl. 1), S53–S77. 10.1016/S0005-7967(99)00050-9 10402696

[B69] Teasdale J. D. (1999b). Metacognition, mindfulness and the modification of mood disorders. Clin. Psychol. Psychother. 6, 146–155. 10.1002/(SICI)1099-0879(199905)6:2<146::AID-CPP195>3.0.CO;2-E

[B70] Teasdale J. D. Chaskalson M. (2011a). How does mindfulness transform suffering? I: the nature and origins of dukkha. Contemp Buddhism 12, 89–102. 10.1080/14639947.2011.564824

[B71] Teasdale J. D. Chaskalson M. (2011b). How does mindfulness transform suffering? II: the transformation of dukkha. Contemp Buddhism 12, 103–124. 10.1080/14639947.2011.564826

[B72] Teasdale J. D. Moore R. G. Hayhurst H. Pope M. Williams S. Segal Z. V. (2002). Metacognitive awareness and prevention of relapse in depression: empirical evidence. J. Consult. Clin. Psychol. 70, 275–287. 10.1037/0022-006X.70.2.275 11952186

[B73] Teasdale J. D. Segal Z. Williams J. M. (1995). How does cognitive therapy prevent depressive relapse and why should attentional control (mindfulness) training help? Behav. Res. Ther. 33, 25–39. 10.1016/0005-7967(94)E0011-7 7872934

[B74] Tecce J. J. (1972). Contingent negative variation (CNV) and psychological processes in man. Psychol. Bull. 77, 73–108. 10.1037/h0032177 4621420

[B75] Tecce J. J. Cattanach L. (1993). Contingent negative variation (CNV), in Electroencephalography: Basic Principles, Clinical Applications and Related Fields, 3rd Edn., eds Niedermeyer E. Lopes da Silva F. (Baltimore, MD: Williams and Wilkins), 887–910.

[B76] Tejaniya S. U. (2016). When Awareness Becomes Natural: A Guide to Cultivating Mindfulness in Everyday Life. Boulder, CO: Shambhala.

[B77] Travis F. Tecce J. Arenander A. Wallace R. K. (2002). Patterns of EEG coherence, power, and contingent negative variation characterize the integration of transcendental and waking states. Biol. Psychol. 61, 293–319. 10.1016/S0301-0511(02)00048-0 12406612

[B78] Travis F. Tecce J. J. Guttman J. (2000). Cortical plasticity, contingent negative variation, and transcendent experiences during practice of the Transcendental Meditation technique. Biol. Psychol. 55, 41–55. 10.1016/S0301-0511(00)00063-6 11099807

[B79] Trivedi M. H. Rush A. J. Ibrahim H. M. Carmody T. J. Biggs M. M. Suppes T.. (2004). The inventory of depressive symptomatology, clinician rating (IDS-C) and self-report (IDS-SR), and the quick inventory of depressive symptomatology, clinician rating (QIDS-C) and self-report (QIDS-SR) in public sector patients with mood disorders: a psychometric evaluation. Psychol. Med. 34, 73–82. 10.1017/S0033291703001107 14971628

[B80] Watson D. Clark L. A. Tellegen A. (1988). Development and validation of brief measures of positive and negative affect: the PANAS scale. J. Pers. Soc. Psychol. 54, 1063–1070. 10.1037/0022-3514.54.6.1063 3397865

[B81] Williams J. M. G. Kabat-Zinn J. (2011). Mindfulness: diverse perspectives on its meaning, origins, and multiple applications at the intersection of science and Dharma. Contemp Buddhism 12, 1–18. 10.1080/14639947.2011.564811

[B82] Wittchen H.-U. Zaudig M. Fydrich T. (1997). Strukturiertes Klinisches Interview für DSM-IV. Göttingen: Hogrefe.

[B83] Zoellner L. A. Sacks M. B. Foa E. B. (2003). Directed forgetting following mood induction in chronic posttraumatic stress disorder patients. J. Abnorm. Psychol. 112, 508–514. 10.1037/0021-843X.112.3.508 12943029

