# Supplementary material for: Measuring Mindfulness: A Psychophysiological Approach
Source: Front Hum Neurosci. 2018 Jun 28;12:249. doi: 10.3389/fnhum.2018.00249 (PMC6031749; doi:10.3389/fnhum.2018.00249)
Supplement: Supplementary file 2 [file Presentation_1.PDF]

Supplementary Material: Measuring Mindfulness: A Psychophysiological Approach

Vladimir Bostanov [1], Lilian Ohlrogge [2], Rita Britz [2], Martin Hautzinger [2], and Boris Kothoubey [1,*]

*Correspondence:
Boris Kothoubey, Institute of Medical Psychology and Behavioral Neurobiology, University of Tübingen, Silcherstr. 5, D-72076 Tübingen, Germany
boris.kotchoubey@uni-tuebingen.de

1 SUPPLEMENTARY DATA

The MP4 video contains the written and spoken instructions (German) of the mood & rumination induction procedure.

The ZIP archive contains the following data:

- `BostanovEtAlMinfulness_Suppl_ADSdata.csv` – individual participants' weekly average depression symptom (DS) scores as measured by the Allgemeine Depressionsskala (ADS), the German version of the CES-D;
- `BostanovEtAlMinfulness_Suppl_AMPdata.csv` – individual participants' weekly average amount of mindfulness practice (AMP) scores;
- `BostanovEtAlMinfulness_Suppl_ADS_AMP.html` – individual participants' weekly average DS & AMP scores in HTML format for better viewing;
- `BostanovEtAlMinfulness_Suppl_ADSitemsData.csv` – individual participants' weekly scores for each ADS item;
- `BostanovEtAlMinfulness_Suppl_ADSitems.txt` – the German text of the ADS items;
- `BostanovEtAlMinfulness_Suppl_dERPi.dDS.AMP.csv` – individual participants' values of: the pre-post-therapy changes (dERPi) in the event-related brain potentials (ERPs) recorded in the passive mindfulness meditation task; the average depression symptom scores during therapy (DSt), during follow-up (DSf), and the corresponding difference dDS = DSf – DSt; the average amount of mindfulness practice during therapy (AMPt) and during follow-up (AMPf);
- `BostanovEtAlMinfulness_Suppl_qData.csv` – individual participants' scores on various questionnaires etc.;
- `BostanovEtAlMinfulness_Suppl_qData.html` – same as above dataset, but displayed in HTML format for better viewing and preceded by a legend explaining which is what.

2 SUPPLEMENTARY TABLES AND FIGURES

2.1 Tables

Table S1 shows results obtained form the *matched* samples. These are pre-post therapy differences in ERP measures and various questionnaire scores as well as long-term changes in trait measures of rumination and mindfulness.

Mea-sure	ERP task	CT Group N	$M \pm SD$	$\lvert t\rvert$	p	MBCT Group N	$M \pm SD$	$\lvert t\rvert$	p	Both (CT + MBCT) N	$M \pm SD$	$\lvert t\rvert$	p	Difference M	$\lvert t_d\rvert$	p_d
DSt		19	12.89± 5.8			41	11.27± 5.1			60	11.78± 5.4			-1.62	1.1	0.28
DSf		19	10.96± 6.4			41	10.58± 5.3			60	10.70± 5.6			-0.39	0.2	0.81
dDS		14	-2.54± 4.0	2.3	0.04	14	-0.94± 3.5	1.0	0.34	28	-1.74± 3.8	2.4	0.02	1.59	1.1	0.28
dERPi	P	20	0.72± 0.5			20	0.47± 0.6			40	0.60± 0.6			-0.25	1.4	0.17
dERPi	A	18	0.97± 0.7			18	0.91± 0.6			36	0.94± 0.6			-0.06	0.3	0.78
dLCNV	P	20	-2.05± 8.3	1.1	0.28	20	4.40±11.1	1.8	0.04*					6.45	2.1	0.02*
dTMSd	P	19	-0.47± 5.4	0.4	0.70	19	2.58± 5.4	2.1	0.05	38	1.05± 5.5	1.2	0.25	3.05	1.7	0.09
dTMSc	P	19	0.26± 4.1	0.3	0.78	19	0.26± 5.2	0.2	0.83	38	0.26± 4.6	0.4	0.72	0.00	0.0	1.00
dTMSd	A	20	0.25± 7.6	0.1	0.88	20	0.90± 6.6	0.6	0.55	40	0.57± 7.0	0.5	0.61	0.65	0.3	0.77
dTMSc	A	20	-0.15± 5.8	0.1	0.91	20	0.65± 6.2	0.5	0.65	40	0.25± 5.9	0.3	0.79	0.80	0.4	0.67
dMR	A	15	2.00±10.3	0.8	0.46	15	4.53± 9.1	1.9	0.07	30	3.27± 9.6	1.9	0.07	2.53	0.7	0.48
dPANAS		20	1.90± 8.1	1.0	0.31	20	1.20±14.1	0.4	0.71	40	1.55±11.3	0.9	0.39	-0.70	0.2	0.85
dRSQsy		14	-5.21± 4.6	4.2	0*	14	-2.57± 4.5	2.2	0.05	28	-3.89± 4.7	4.4	0*	2.64	1.5	0.14
dRSQse		14	-4.43± 2.7	6.1	0*	14	-1.57± 3.4	1.7	0.11	28	-3.00± 3.4	4.7	0*	2.86	2.4	0.02
dRSQdi		14	3.86± 4.2	3.5	0.00	14	0.29± 3.8	0.3	0.78	28	2.07± 4.3	2.5	0.02	-3.57	2.4	0.03
dMAAS		14	5.36±11.7	1.7	0.11	14	1.71± 8.8	0.7	0.48	28	3.54±10.3	1.8	0.08	-3.64	0.9	0.36

Table S1. Pre-post-therapy differences and long-term changes in the matched samples. Pre-post-therapy differences: dERPi, multivariate difference in individual ERPs; dLCNV, univariate amplitude differences in late CNV; dTMSd and dTMSc, changes in the *state* mindfulness sub-scores, TMS-decentering and TMS-curiosity, respectively; dMR, changes in the motor response in the active meditation task. dPANAS, changes in the sum of the decrease in positive affect and the increase in negative affect (dPA − dNA) after the mood & rumination induction reflecting the pre-post-therapy change in participants' reaction to the procedure. Long-term changes: dDS = DSf − DSt, where DSf is the mean DS score during follow-up and DSt is the mean DS score during therapy); dRSQsy, dRSQse, dRSQdi, and dMAAS, changes in symptom-focused rumination, self-focused rumination, distraction, and *trait* mindfulness, respectively), over a period of more than 60 weeks, from the beginning of therapy till the end of the one-year follow-up. The passive meditation ERP task is denoted by "P", the active one by "A". N is the number of participants in the corresponding sample; M is the mean value of the corresponding difference measure; SD is the standard deviation; and t and p are the results of Students's t-tests checking whether the mean is different from zero; $|t_d|$ and p_d Students's t-tests checking whether the mean in the CT group is different from the mean in the MBCT group. The p-values are *not* corrected for multiple comparisons; 0* denotes $p < 0.0005$, i.e., p-values that remain significant after Bonferroni correction for $2 \times 14 = 28$ comparisons; 0.02* is the significant p-value resulting from the planned *one-sided* t-test of the difference between dLCNV in the CT group and dLCNV in the MBCT group.

2.2 Figures

Grand average ERPs obtained in the *active* mindfulness meditation task are displayed in Figures S1 and S2.

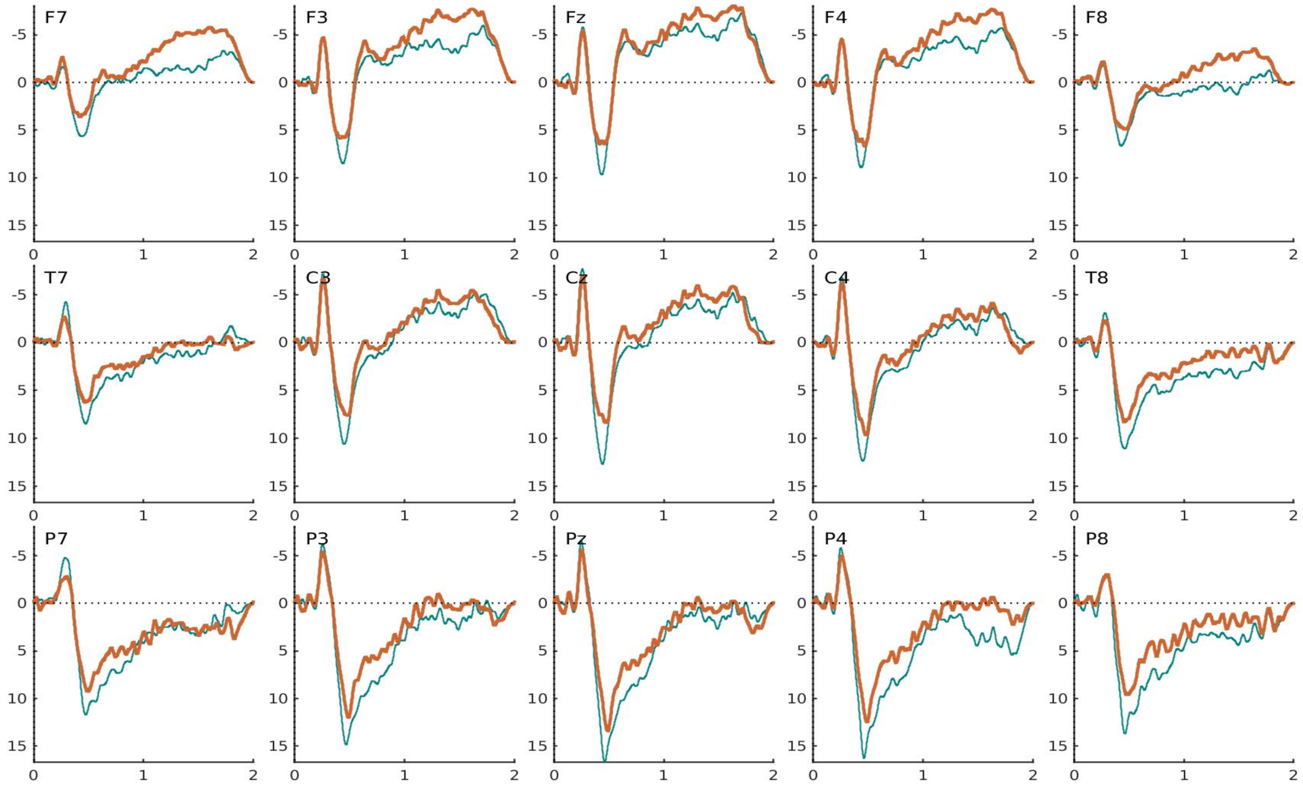

(a) ERPs obtained from the CT group before (thin green line) and after (thick brown line) 8 weeks of therapy.

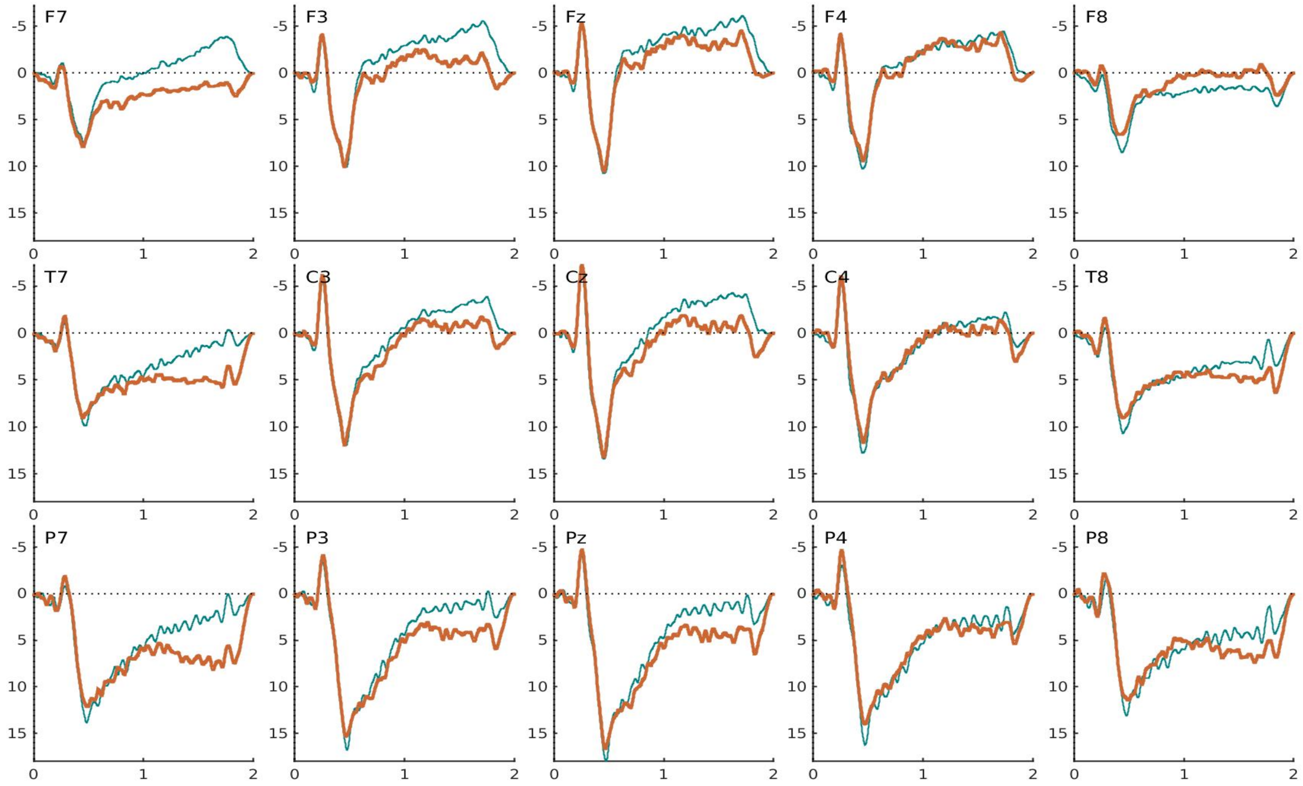

(b) ERPs obtained from the MBCT group before (thin green line) and after (thick brown line) 8 weeks of training.

Figure S1: Grand average ERPs obtained in the active mindfulness meditation task.

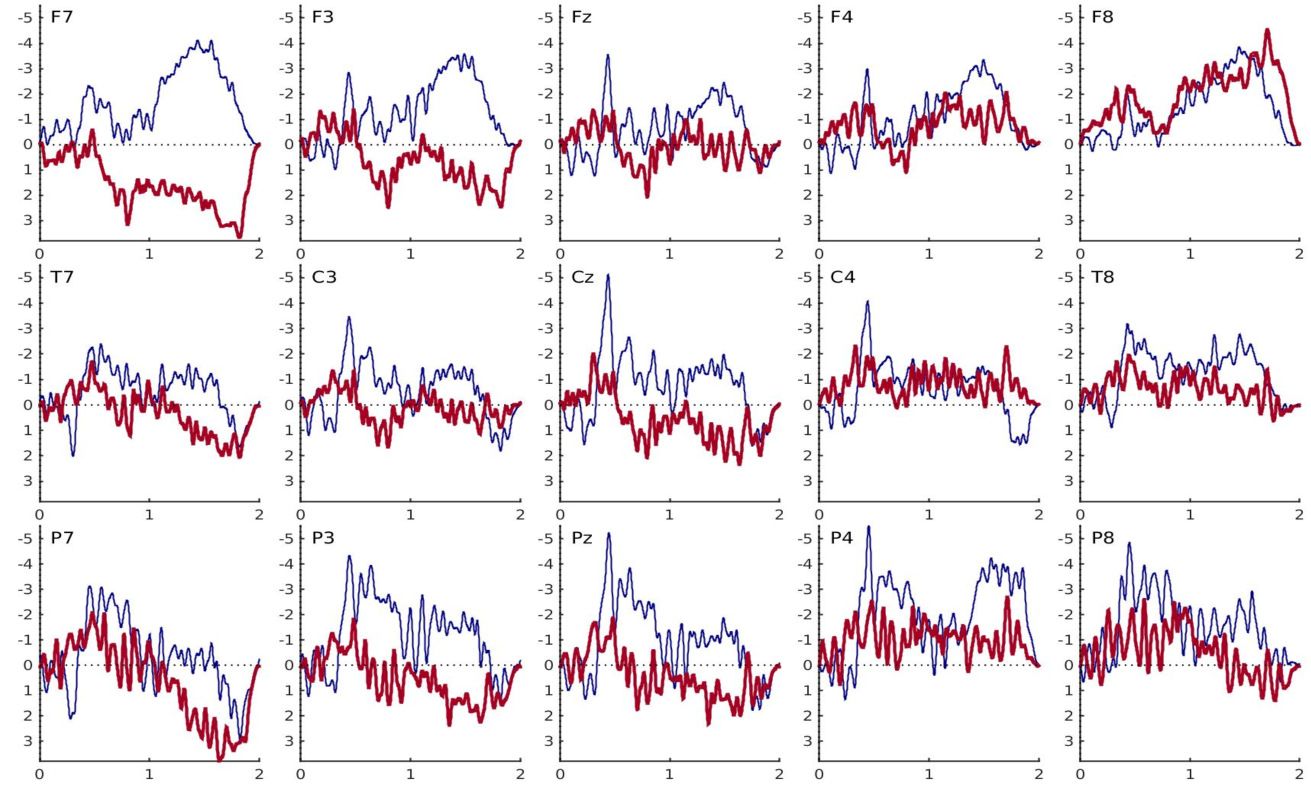

(a) Pre-post-therapy difference ERP in the CT group (thin blue line) and in the MBCT group (thick red line).

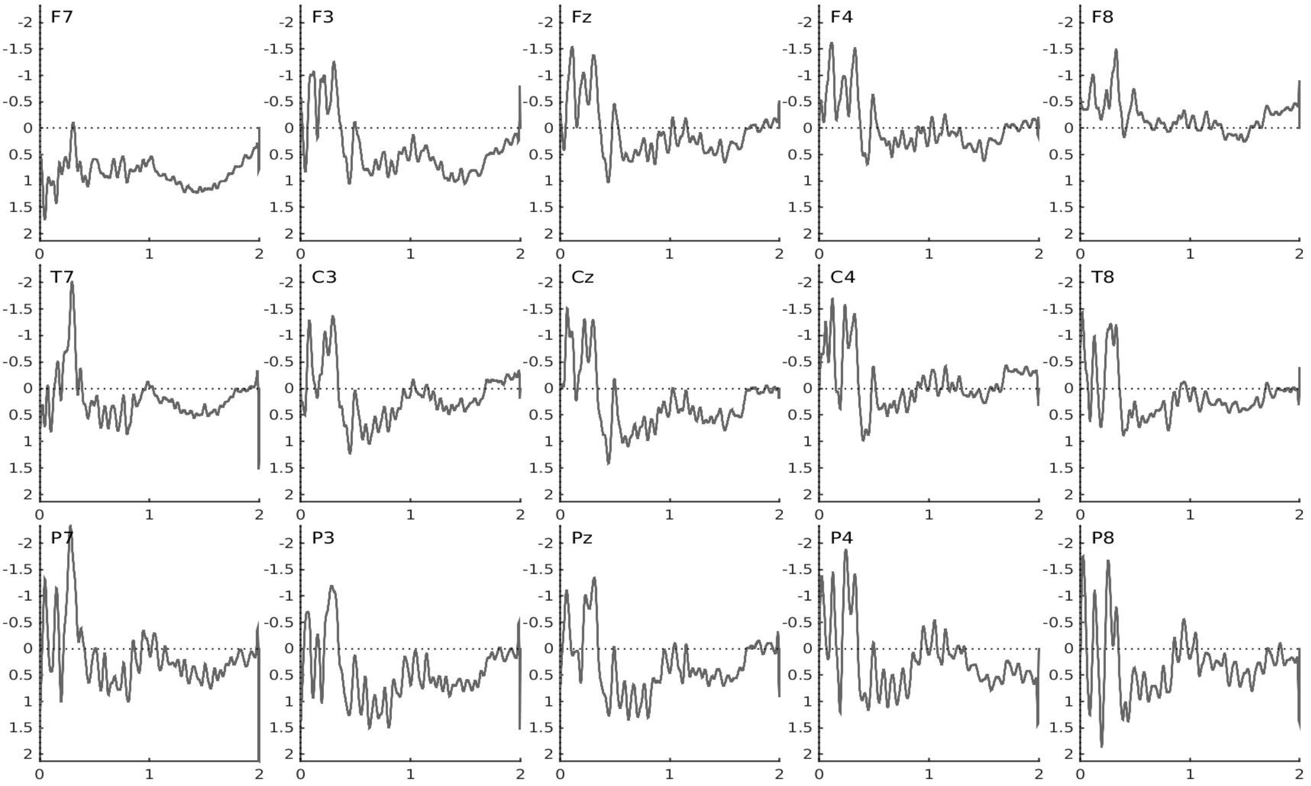

(b) Student's t-test of the difference between the ERP change in the CT group and the that in the MBCT group.

Figure S2: Pre-post-therapy change in the ERPs obtained in the active mindfulness meditation task.
