# Supplementary material for: Measuring Mindfulness: A Psychophysiological Approach
Source: Front Hum Neurosci. 2018 Jun 28;12:249. doi: 10.3389/fnhum.2018.00249 (PMC6031749; doi:10.3389/fnhum.2018.00249)
Supplement: Supplementary file 3 [file Data_Sheet_1.ZIP › BostanovEtAlMinfulness_Suppl_data/BostanovEtAlMinfulness_Suppl_ADS_AMP.html]

ADS etc.


# ADS Total Scores, Changes in Medication & Amounts of Mindfulness Practice

## Legend

|  |  |
| --- | --- |
|  |  |
| t = 1 | Therapy: CT |
| t = 2 | Therapy: MBCT |
| g | Group |
|  |  |
| tN (green) | Nth Therapy Week |
| fN (blue) | Nth Follow-up Week |
| ? (yellow) | Missing Data |
|  |  |
| ADS | ADS Total Score |
| ChM | Change in Medication |
| AMP | Amount of Mindfulness Practice |

## Data

| id | t | g |  | t1 | t2 | t3 | t4 | t5 | t6 | t7 | t8 | f1 | f2 | f3 | f4 | f5 | f6 | f7 | f8 | f9 | f10 | f11 | f12 | f13 | f14 | f15 | f16 | f17 | f18 | f19 | f20 | f21 | f22 | f23 | f24 | f25 | f26 | f27 | f28 | f29 | f30 | f31 | f32 | f33 | f34 | f35 | f36 | f37 | f38 | f39 | f40 | f41 | f42 | f43 | f44 | f45 | f46 | f47 | f48 | f49 | f50 | f51 | f52 |
| --- | --- | --- | --- | --- | --- | --- | --- | --- | --- | --- | --- | --- | --- | --- | --- | --- | --- | --- | --- | --- | --- | --- | --- | --- | --- | --- | --- | --- | --- | --- | --- | --- | --- | --- | --- | --- | --- | --- | --- | --- | --- | --- | --- | --- | --- | --- | --- | --- | --- | --- | --- | --- | --- | --- | --- | --- | --- | --- | --- | --- | --- | --- | --- |
| ukgr6ibi | 1 | A | **ADS ChM AMP** | 23 0 0 | ? | ? | 24 0 0 | 22 0 0 | 16 0 0 | 16 0 0 | ? | 22 0 0 | 20 0 0 | 23 0 0 | 22 0 0 | 18 0 0 | 15 0 0 | 20 0 0 | 13 0 0 | 14 0 0 | 10 0 0 | 16 0 0 | 14 0 0 | 27 0 0 | 22 0 0 | 12 0 0 | 12 0 0 | 8 0 0 | 14 0 0 | 11 0 0 | 32 0 0 | 15 0 0 | 13 0 0 | 12 0 0 | 8 0 0 | 9 0 0 | 15 0 0 | 13 0 0 | 15 0 0 | 15 0 0 | 12 0 0 | 12 0 0 | 16 0 0 | 13 0 0 | 10 0 0 | 12 0 0 | 10 0 0 | 9 0 0 | 10 0 0 | 11 0 0 | 28 0 0 | 14 0 0 | 12 0 0 | 12 0 0 | 13 0 0 | 18 0 0 | 16 0 0 | 17 0 0 | 19 0 0 | 14 0 0 | 17 0 0 | 26 0 0 | 18 0 0 |
| lunu6vmb | 1 | A | **ADS ChM AMP** | 7 0 0 | 5 0 0 | 8 0 0 | 6 0 0 | 13 0 0 | 8 0 0 | 6 0 0 | 7 0 0 | 7 0 0 | 18 0 0 | 15 0 0 | 9 0 0 | 16 0 0 | 12 0 0 | 14 0 0 | 17 0 0 | 14 0 0 | 4 0 0 | 8 0 0 | 10 0 0 | 10 0 0 | 5 1 0 | 4 0 0 | 6 0 0 | 4 0 0 | 5 0 0 | 4 0 0 | 4 0 0 | 4 0 0 | 7 0 0 | 7 0 0 | 6 0 0 | 4 0 0 | 4 0 0 | 3 0 0 | 19 0 0 | 18 0 0 | 10 0 0 | 4 0 0 | 4 0 0 | 5 0 0 | 11 0 0 | 9 0 0 | 11 0 0 | 6 0 0 | 7 0 0 | 5 0 0 | 12 0 0 | 14 0 0 | 16 0 0 | 27 10 0 | 10 0 0 | 23 0 0 | 12 0 0 | 15 0 0 | 14 0 0 | 10 0 0 | 9 -10 0 | 10 0 0 | 9 0 0 |
| lrmj6jew | 1 | A | **ADS ChM AMP** | 22 0 0 | 25 0 0 | 23 0 0 | 29 0 0 | 23 0 0 | 25 0 0 | 30 0 0 | 28 0 0 | ? | ? | 26 0 0 | 27 0 0 | 27 0 0 | 28 0 0 | 24 0 0 | 23 0 0 | 26 0 0 | 26 0 0 | 25 0 0 | 25 0 0 | 28 0 0 | 24 0 0 | 26 0 0 | 27 0 0 | 27 0 0 | 22 0 0 | 28 0 0 | 25 0 0 | 29 0 0 | 28 10 0 | 25 0 0 | 22 0 0 | 23 0 0 | 23 0 0 | 17 0 0 | 22 0 0 | 22 0 0 | 24 0 0 | 22 0 0 | 25 0 0 | 26 0 0 | 18 0 0 | 19 0 0 | 22 0 0 | 23 0 0 | 24 0 0 | 22 0 0 | 25 0 0 | 25 0 0 | 22 0 0 | 24 0 0 | 25 0 0 | 18 0 0 | 24 0 0 | 24 0 0 | 18 0 0 | 19 0 0 | 25 0 0 | 22 0 0 | 24 0 0 |
| gpod8wgm | 1 | A | **ADS ChM AMP** | 21 0 0 | 26 10 0 | 11 0 0 | 8 0 0 | 5 0 0 | 4 0 0 | 22 0 0 | 23 0 0 | 15 0 0 | 34 0 0 | 19 0 0 | 20 0 0 | 14 0 0 | 23 0 0 | 16 0 0 | 8 0 0 | 13 0 0 | 6 0 0 | 14 0 0 | 21 0 0 | 7 0 0 | 29 0 0 | 25 0 0 | 29 0 0 | 25 0 0 | 10 -10 0 | 11 -10 0 | 5 10 0 | 5 0 0 | 8 0 0 | 18 0 0 | 13 0 0 | 4 0 0 | 8 0 0 | 8 0 0 | 6 0 0 | 8 0 0 | 15 0 0 | 7 0 0 | 17 0 0 | 7 0 0 | 12 0 0 | 9 0 0 | 24 0 0 | 11 0 0 | 8 0 0 | 9 0 0 | 9 0 0 | 5 0 0 | 7 0 0 | 9 0 0 | 24 0 0 | 13 10 0 | 28 0 0 | 20 0 0 | 4 0 0 | 4 0 0 | 4 0 0 | 1 0 0 | 6 0 0 |
| ronr6tsn | 1 | A | **ADS ChM AMP** | 10 0 0 | 7 0 0 | 6 0 0 | 7 0 0 | 8 0 0 | 9 0 0 | 18 0 0 | 12 0 0 | 11 0 0 | 10 0 0 | 8 0 0 | 7 0 0 | 19 0 0 | 14 0 0 | 10 0 0 | 10 0 0 | 9 0 0 | 10 0 0 | 12 0 0 | 14 0 0 | 11 0 0 | 10 0 0 | 10 0 0 | 13 0 0 | 13 0 0 | 14 0 0 | 12 0 0 | 10 0 0 | 13 0 0 | 7 0 0 | 7 0 0 | 9 0 0 | 11 0 0 | 9 0 0 | 8 0 0 | 8 0 0 | 6 0 0 | 6 0 0 | 7 0 0 | 9 0 0 | 7 0 0 | 8 0 0 | 10 0 0 | 11 0 0 | 15 0 0 | 14 0 0 | 12 0 0 | 9 0 0 | 11 0 0 | 9 0 0 | 9 0 0 | 8 0 0 | 9 0 0 | 17 0 0 | 9 0 0 | 11 0 0 | 13 0 0 | 10 0 0 | 9 0 0 | 20 0 0 |
| iuhu6gyr | 1 | A | **ADS ChM AMP** | 21 0 0 | 22 0 0 | 12 0 0 | 9 0 0 | 11 0 0 | 15 0 0 | 10 0 0 | 14 0 0 | 18 0 0 | 10 0 0 | 16 0 0 | 10 0 0 | 10 0 0 | 9 0 0 | 8 0 0 | 12 0 0 | 9 0 0 | 4 0 0 | 6 0 0 | 7 0 0 | 13 0 0 | 15 0 0 | 10 0 0 | 2 0 0 | 6 0 0 | 2 0 0 | 3 0 0 | 5 0 0 | 5 0 0 | 6 0 0 | 2 0 0 | 0 0 0 | 2 0 0 | 2 0 0 | 3 0 0 | 0 0 0 | 0 0 0 | 0 0 0 | 0 0 0 | 0 0 0 | 0 0 0 | 0 0 0 | 3 0 0 | 1 0 0 | 0 0 0 | 0 10 0 | 0 0 0 | 3 0 0 | 3 0 0 | 0 0 0 | 2 0 0 | 7 0 0 | 5 0 0 | 1 0 0 | 0 0 0 | 0 0 0 | 0 0 0 | 0 0 0 | 0 0 0 | 0 0 0 |
| irjh8pxw | 1 | A | **ADS ChM AMP** | 2 0 0 | 8 0 0 | 6 0 0 | 2 0 0 | 6 0 0 | 5 0 0 | 4 0 0 | 0 0 0 | 0 0 0 | 0 0 0 | 0 0 0 | 2 0 0 | 4 0 0 | 1 0 0 | 3 0 0 | 2 0 0 | 2 0 0 | 3 0 0 | 8 0 0 | 13 0 0 | 14 0 0 | 7 0 0 | 6 0 0 | 1 0 0 | 0 0 0 | 0 0 0 | 1 0 0 | 0 0 0 | 0 0 0 | 2 0 0 | 0 0 0 | 0 0 0 | 0 0 0 | 7 0 0 | 8 0 0 | 5 0 0 | 1 0 0 | 2 0 0 | 0 0 0 | 0 0 0 | 0 0 0 | 4 0 0 | 2 0 0 | 0 0 0 | 7 0 0 | 0 0 0 | 2 0 0 | 1 0 0 | 2 0 0 | 4 0 0 | 1 0 0 | 0 0 0 | 0 0 0 | 0 0 0 | 2 0 0 | 0 0 0 | 0 0 0 | 8 0 0 | 1 0 0 | 0 0 0 |
| npod6ehm | 1 | A | **ADS ChM AMP** | 18 0 0 | 22 0 0 | 7 0 0 | 10 0 0 | 1 0 0 | 5 0 0 | 6 0 0 | 0 0 0 | 0 0 0 | 3 0 0 | 7 0 0 | 0 0 0 | 4 0 0 | 17 0 0 | 3 0 0 | 0 0 0 | 2 0 0 | 1 0 0 | 2 0 0 | 3 0 0 | 1 0 0 | 7 0 0 | 4 0 0 | 2 0 0 | 0 0 0 | 5 0 0 | 12 0 0 | 6 0 0 | 0 0 0 | 4 0 0 | 1 0 0 | 4 0 0 | 4 0 0 | 5 0 0 | 3 0 0 | 8 0 0 | 9 0 0 | 3 0 0 | 3 0 0 | 1 0 0 | 2 0 0 | 4 0 0 | 1 0 0 | 6 0 0 | 6 0 0 | 2 0 0 | 1 0 0 | 7 0 0 | 3 0 0 | 10 0 0 | 7 0 0 | 3 0 0 | 4 0 0 | 4 0 0 | 1 0 0 | 6 0 0 | 8 0 0 | 5 0 0 | 12 0 0 | 16 0 0 |
| uger6lhp | 1 | C | **ADS ChM AMP** | 12 0 0 | 10 0 0 | 10 0 0 | 11 0 0 | 8 0 0 | 10 0 0 | 14 0 0 | 11 0 0 | 11 0 0 | 13 0 0 | 10 0 0 | 9 0 0 | 3 0 0 | 7 0 0 | 8 0 0 | 8 0 0 | 8 0 0 | 14 0 0 | 8 0 0 | 7 0 0 | 6 0 0 | 5 0 0 | 5 0 0 | 8 0 0 | 11 0 0 | 10 0 0 | 5 0 0 | 5 0 0 | 10 0 0 | 8 0 0 | 10 0 0 | 12 0 0 | 12 0 0 | 7 0 0 | 8 -10 0 | 6 -10 0 | 9 0 0 | 8 0 0 | 7 0 0 | 9 0 0 | 8 0 0 | 8 0 0 | 9 0 0 | 4 0 0 | 6 0 0 | 11 0 0 | 6 0 0 | 8 0 0 | 12 0 0 | 12 0 0 | 8 0 0 | 7 0 0 | 6 0 0 | 8 0 0 | 8 0 0 | 8 0 0 | 17 0 0 | 15 0 0 | 10 0 0 | 10 0 0 |
| lkat5awd | 1 | C | **ADS ChM AMP** | 14 0 0 | 2 0 0 | 2 0 0 | 12 0 0 | 2 0 0 | 4 0 0 | 8 0 0 | 2 0 0 | 10 0 0 | 4 0 0 | 13 0 0 | 9 0 0 | 5 0 0 | 8 0 0 | 4 0 0 | 4 0 0 | 2 0 0 | 2 0 0 | 2 0 0 | 2 0 0 | 6 0 0 | 4 0 0 | 4 0 0 | 4 0 0 | 4 0 0 | 7 0 0 | 5 0 0 | 4 0 0 | 10 0 0 | 9 0 0 | 12 0 0 | 3 0 0 | 14 0 0 | 17 0 0 | 4 0 0 | 6 0 0 | 9 0 0 | 5 0 0 | 18 0 0 | 13 0 0 | 4 0 0 | 4 0 0 | 6 0 0 | 14 0 0 | 18 0 0 | 6 0 0 | 10 0 0 | 3 0 0 | 13 0 0 | 6 0 0 | 24 0 0 | 10 0 0 | 4 0 0 | 12 0 0 | 5 0 0 | 5 0 0 | 4 0 0 | 3 0 0 | 3 0 0 | 4 0 0 |
| fdvd4bzu | 1 | C | **ADS ChM AMP** | 16 0 0 | 20 0 0 | 15 0 0 | 9 0 0 | 5 0 0 | 10 0 0 | 7 0 0 | 6 0 0 | 10 0 0 | 2 0 0 | 1 0 0 | 3 0 0 | 1 0 0 | 0 0 0 | 0 0 0 | 1 0 0 | 0 0 0 | 1 0 0 | 1 0 0 | 1 0 0 | 3 0 0 | 12 0 0 | 11 0 0 | 22 0 0 | 12 0 0 | 19 0 0 | 9 0 0 | 16 0 0 | 6 0 0 | 3 0 0 | 9 0 0 | 4 0 0 | 4 0 0 | 4 0 0 | 4 0 0 | 2 0 0 | 1 0 0 | 1 0 0 | 0 0 0 | 0 0 0 | 0 0 0 | 0 0 0 | 0 0 0 | 1 0 0 | 0 0 0 | 0 0 0 | 0 0 0 | 0 0 0 | 0 0 0 | 3 0 0 | 10 0 0 | 6 0 0 | 5 0 0 | 13 0 0 | 12 0 0 | 6 0 0 | 5 0 0 | 5 0 0 | 7 0 0 | 1 0 0 |
| rhua5phi | 1 | C | **ADS ChM AMP** | 24 0 0 | 18 0 0 | 21 0 0 | 21 0 0 | 16 0 0 | 15 0 0 | 12 0 0 | 13 0 0 | 10 0 0 | 16 0 0 | 27 0 0 | 23 0 0 | 18 0 0 | 18 0 0 | 21 0 0 | 15 0 0 | 14 0 0 | 14 0 0 | 20 0 0 | 14 0 0 | 18 0 0 | 13 0 0 | 14 0 0 | 15 0 0 | 15 0 0 | 12 0 0 | 12 0 0 | 13 0 0 | 14 0 0 | 12 0 0 | 18 0 0 | 14 0 0 | 10 0 0 | 11 0 0 | 12 0 0 | 16 0 0 | 19 0 0 | 11 0 0 | 10 0 0 | 12 0 0 | 10 0 0 | 11 0 0 | 5 0 0 | 7 0 0 | 15 0 0 | 12 0 0 | 12 0 0 | 23 0 0 | 17 0 0 | 13 0 0 | 14 0 0 | 16 0 0 | 11 0 0 | 11 0 0 | 16 0 0 | 11 0 0 | 10 0 0 | 7 0 0 | 14 0 0 | 11 0 0 |
| kuyk4bcl | 1 | C | **ADS ChM AMP** | 21 0 0 | 22 0 0 | 13 0 0 | 19 0 0 | 17 0 0 | 19 0 0 | 22 0 0 | 19 0 0 | 10 0 0 | 14 0 0 | 17 0 0 | 16 0 0 | 17 0 0 | 18 0 0 | 17 0 0 | 28 0 0 | 27 0 0 | 24 0 0 | 23 0 0 | 18 0 0 | 18 0 0 | 9 0 0 | 13 0 0 | 24 0 0 | 21 0 0 | 25 0 0 | 16 0 0 | 9 0 0 | 21 0 0 | 21 0 0 | 18 0 0 | 17 0 0 | 21 10 0 | 16 0 0 | 24 0 0 | 21 0 0 | 14 0 0 | 21 0 0 | 16 0 0 | 27 0 0 | 24 0 0 | 24 0 0 | 25 10 0 | 25 10 0 | 29 10 0 | 23 0 0 | 26 0 0 | 22 0 0 | 22 0 0 | 21 0 0 | 23 0 0 | 21 0 0 | 18 0 0 | 19 0 0 | 19 0 0 | 25 0 0 | 21 0 0 | 18 0 0 | 18 0 0 | 22 0 0 |
| xvip2ttr | 1 | C | **ADS ChM AMP** | 7 0 0 | 9 0 0 | 9 0 0 | 7 0 0 | 17 -10 0 | 11 0 0 | 6 0 0 | 8 0 0 | 8 0 0 | 6 0 0 | 10 0 0 | 8 0 0 | 8 0 0 | 8 0 0 | 8 0 0 | 13 0 0 | 9 0 0 | 13 0 0 | 10 0 0 | 6 0 0 | 8 -10 0 | 10 0 0 | 6 0 0 | 6 0 0 | 7 -10 0 | 7 0 0 | 7 0 0 | 9 0 0 | 13 0 0 | 10 0 0 | 7 0 0 | 13 0 0 | 7 0 0 | 7 0 0 | 16 0 0 | 16 0 0 | 9 0 0 | 8 0 0 | 2 0 0 | 24 0 0 | 26 10 0 | 10 0 0 | 8 0 0 | 11 0 0 | 9 0 0 | 11 0 0 | 7 0 0 | 9 0 0 | 12 0 0 | 12 0 0 | 11 0 0 | 8 0 0 | 8 0 0 | 8 0 0 | 5 0 0 | 8 0 0 | 8 0 0 | 7 0 0 | 6 0 0 | 6 0 0 |
| orer5lwy | 1 | C | **ADS ChM AMP** | 7 0 0 | 6 0 0 | 18 0 0 | 7 0 0 | 12 0 0 | 11 0 0 | 19 0 0 | 17 0 0 | 7 0 0 | 4 0 0 | 5 0 0 | 18 0 0 | 9 0 0 | 22 0 0 | 4 0 0 | 1 0 0 | 1 0 0 | 0 0 0 | 1 0 0 | 1 0 0 | 12 0 0 | 4 0 0 | 1 0 0 | 4 0 0 | 3 0 0 | 2 0 0 | 9 0 0 | 4 0 0 | 7 0 0 | 6 0 0 | 2 0 0 | 13 0 0 | 4 0 0 | 8 0 0 | 14 0 0 | 12 0 0 | 8 0 0 | 8 0 0 | 7 0 0 | 6 0 0 | 7 0 0 | 6 0 0 | 6 0 0 | 8 0 0 | 9 0 0 | 7 0 0 | 5 0 0 | 6 0 0 | 4 0 0 | 4 0 0 | 4 0 0 | 0 0 0 | 3 0 0 | 2 0 0 | 4 0 0 | 2 0 0 | 1 0 0 | 1 0 0 | 3 0 0 | 1 0 0 |
| edbu8yrd | 1 | C | **ADS ChM AMP** | 20 0 0 | 26 0 0 | 21 0 0 | 15 0 0 | 10 0 0 | 14 0 0 | 11 0 0 | 13 0 0 | 11 0 0 | 17 0 0 | 10 0 0 | 16 0 0 | 21 0 0 | 19 0 0 | 9 0 0 | 17 0 0 | 17 0 0 | 16 0 0 | 18 0 0 | 14 0 0 | 20 0 0 | 17 0 0 | 20 0 0 | 18 0 0 | 18 0 0 | 21 0 0 | 22 0 0 | 17 0 0 | 23 0 0 | 22 0 0 | 21 0 0 | 28 0 0 | 13 0 0 | 21 0 0 | 17 0 0 | 18 0 0 | 21 0 0 | 17 0 0 | 27 0 0 | 28 0 0 | 21 0 0 | 26 0 0 | 20 0 0 | 30 0 0 | 29 0 0 | 20 0 0 | 30 0 0 | 31 0 0 | 35 0 0 | 29 0 0 | 30 0 0 | 33 0 0 | 29 0 0 | 29 0 0 | 27 0 0 | 27 0 0 | 29 0 0 | 29 0 0 | 26 0 0 | 25 0 0 |
| uwxu5xig | 1 | G | **ADS ChM AMP** | 18 0 0 | 18 0 0 | 16 0 0 | 5 0 0 | 4 0 0 | 0 0 0 | 5 0 0 | 10 0 0 | 1 0 0 | 1 0 0 | 1 0 0 | 0 0 0 | 2 0 0 | 0 0 0 | 4 0 0 | 10 0 0 | ? | 0 0 0 | 8 0 0 | 0 0 0 | 4 0 0 | 2 0 0 | 0 0 0 | 0 0 0 | 3 0 0 | ? | 0 0 0 | ? | ? | ? | ? | ? | ? | ? | ? | ? | ? | ? | ? | ? | ? | ? | ? | ? | ? | ? | ? | ? | ? | ? | ? | ? | ? | ? | ? | ? | ? | ? | ? | ? |
| auxp2tem | 1 | G | **ADS ChM AMP** | 8 0 0 | 9 0 0 | 3 0 0 | 10 0 0 | 3 0 0 | 7 0 0 | 3 0 0 | 3 0 0 | 4 0 0 | 2 0 0 | 2 0 0 | 0 0 0 | 0 0 0 | 0 0 0 | 1 0 0 | 15 0 0 | 14 0 0 | 11 0 0 | 4 0 0 | 8 0 0 | 2 10 0 | 3 0 0 | 4 0 0 | 3 0 0 | 3 0 0 | 3 0 0 | 3 0 0 | 2 0 0 | 4 0 0 | 4 0 0 | 2 0 0 | 3 0 0 | 3 0 0 | 5 0 0 | 5 0 0 | 4 0 0 | 8 0 0 | 4 0 0 | 2 0 0 | 8 0 0 | 5 0 0 | 7 0 0 | 12 0 0 | 10 0 0 | 8 0 0 | 6 10 0 | 9 0 0 | 11 0 0 | 26 0 0 | 11 0 0 | 10 0 0 | 12 0 0 | 22 0 0 | 4 0 0 | 13 0 0 | 7 0 0 | 12 0 0 | 5 0 0 | 13 0 0 | 13 0 0 |
| hpod5kla | 1 | G | **ADS ChM AMP** | 7 0 0 | 13 0 0 | 6 0 0 | 13 0 0 | 4 0 0 | 19 0 0 | 18 0 0 | 14 0 0 | 23 0 0 | 4 0 0 | 4 0 0 | 9 0 0 | 7 0 0 | 10 0 0 | 10 0 0 | 2 0 0 | 3 0 0 | 4 0 0 | 2 0 0 | 15 0 0 | 11 0 0 | 6 0 0 | 8 0 0 | 10 0 0 | 7 0 0 | 3 0 0 | 18 0 0 | 11 0 0 | 1 0 0 | 8 0 0 | 3 0 0 | 7 0 0 | 1 0 0 | 2 0 0 | 6 0 0 | 9 0 0 | 9 0 0 | 23 0 0 | 4 0 0 | 4 0 0 | 7 0 0 | 9 0 0 | 6 0 0 | 9 0 0 | 10 0 0 | 9 0 0 | 0 0 0 | 26 0 0 | 14 0 0 | 6 0 0 | 2 0 0 | 12 0 0 | 11 0 0 | 13 0 0 | 12 0 0 | 11 0 0 | 2 0 0 | 3 0 0 | 5 0 0 | 5 0 0 |
| nuxf5dmp | 1 | G | **ADS ChM AMP** | 11 0 0 | 12 0 0 | 18 0 0 | 17 0 0 | 14 0 0 | 16 0 0 | 14 0 0 | 11 0 0 | 7 0 0 | 6 0 0 | ? | ? | ? | ? | ? | ? | ? | ? | ? | ? | ? | ? | ? | ? | ? | ? | ? | ? | ? | ? | ? | ? | ? | ? | ? | ? | ? | ? | ? | ? | ? | ? | ? | ? | ? | ? | ? | ? | ? | ? | ? | ? | ? | ? | ? | ? | ? | ? | ? | ? |
| nsmu5nph | 1 | G | **ADS ChM AMP** | 27 0 0 | 19 1 0 | 6 1 0 | 29 0 0 | 18 0 0 | 23 0 0 | 20 0 0 | 25 0 0 | 29 0 0 | 25 0 0 | 19 0 0 | 33 0 0 | 21 0 0 | 32 0 0 | 24 10 0 | 31 0 0 | 29 0 0 | 23 0 0 | 30 0 0 | 23 0 0 | 25 0 0 | 29 -10 0 | 30 0 0 | 19 0 0 | 12 -10 0 | 12 1 0 | 11 1 0 | 15 0 0 | 27 0 0 | 18 0 0 | 16 0 0 | 13 0 0 | 10 0 0 | 16 0 0 | 12 0 0 | 15 0 0 | 16 0 0 | 13 0 0 | 17 0 0 | 10 0 0 | 12 0 0 | 23 0 0 | 11 0 0 | 11 0 0 | 18 0 0 | 10 0 0 | 10 0 0 | 12 0 0 | 16 0 0 | 24 0 0 | 13 0 0 | 23 0 0 | 12 0 0 | 8 0 0 | 11 0 0 | 29 0 0 | 7 0 0 | 11 0 0 | 9 0 0 | 18 0 0 |
| wuxv5xog | 1 | G | **ADS ChM AMP** | 8 0 0 | 12 0 0 | 8 0 0 | 10 0 0 | 10 0 0 | 6 0 0 | 9 0 0 | 9 0 0 | ? | ? | ? | ? | ? | ? | ? | ? | ? | ? | ? | ? | ? | ? | ? | ? | ? | ? | ? | ? | ? | ? | ? | ? | ? | ? | ? | ? | ? | ? | ? | ? | ? | ? | ? | ? | ? | ? | ? | ? | ? | ? | ? | ? | ? | ? | ? | ? | ? | ? | ? | ? |
| vper5lkp | 1 | G | **ADS ChM AMP** | 4 0 0 | 5 0 0 | 8 0 0 | 6 0 0 | 2 0 0 | 4 0 0 | 5 0 0 | 3 0 0 | 1 0 0 | 3 0 0 | 0 0 0 | ? | ? | ? | ? | ? | ? | ? | ? | ? | ? | ? | ? | ? | ? | ? | ? | ? | ? | ? | ? | ? | ? | ? | ? | ? | ? | ? | ? | ? | ? | ? | ? | ? | ? | ? | ? | ? | ? | ? | ? | ? | ? | ? | ? | ? | ? | ? | ? | ? |
| orit5aym | 1 | G | **ADS ChM AMP** | 14 0 0 | 14 0 0 | 18 0 0 | ? | ? | 5 0 0 | 5 0 0 | 9 0 0 | 5 0 0 | 6 0 0 | 8 0 0 | 13 0 0 | 8 0 0 | ? | ? | ? | ? | ? | ? | ? | ? | ? | ? | ? | ? | ? | ? | ? | ? | ? | ? | ? | ? | ? | ? | ? | ? | ? | ? | ? | ? | ? | ? | ? | ? | ? | ? | ? | ? | ? | ? | ? | ? | ? | ? | ? | ? | ? | ? | ? |
| uwiu8bon | 2 | B | **ADS ChM AMP** | 10 0 60 | 11 0 20 | 11 0 60 | 7 0 40 | 13 0 60 | 12 0 60 | 10 0 60 | 17 0 60 | 12 0 30 | 11 0 30 | 6 0 30 | 7 0 6 | 12 0 60 | 7 0 60 | 12 0 30 | 11 0 30 | 10 0 30 | 13 0 30 | 7 0 30 | 13 0 30 | 11 0 6 | 14 0 6 | 7 0 30 | 15 0 6 | 14 0 20 | 12 0 20 | 7 0 20 | 7 0 30 | 9 0 20 | 9 0 4 | 10 0  5 | 7 0 20 | 11 0 40 | 10 0  5 | 9 0  5 | 10 0  5 | 11 10  5 | 8 0 10 | 8 0 40 | 7 0 10 | 8 0 40 | 8 0  5 | 8 0 20 | 7 0 20 | 6 0  5 | 5 0  5 | 10 0 20 | 15 0 10 | 11 0 10 | 8 0  5 | 6 0 40 | 10 0 10 | 11 0  5 | 7 0 20 | 8 0  5 | 5 0 40 | 6 0 20 | 7 0 10 | 6 0  5 | 4 0 20 |
| uwxf6wfj | 2 | B | **ADS ChM AMP** | 12 0 60 | 0 0 20 | 0 0 30 | 0 0 20 | 0 0 4 | 0 0 20 | 0 0 20 | 0 0 20 | 0 -10 30 | 0 0 30 | 0 -10 40 | 0 0 40 | 0 0 20 | 0 0 20 | 0 0 20 | 0 0 20 | 0 0 40 | 6 0 20 | 0 0 4 | 0 0 20 | 0 0 20 | 0 0 20 | 0 0 20 | 0 0 20 | 0 0 20 | 0 0 20 | 0 0 20 | 0 0 20 | 0 0 20 | 0 -10 1 | 13 10 1 | 20 10  5 | 19 10 4 | 20 0  5 | 31 0 40 | 30 0 20 | 32 0 4 | 37 10 20 | 28 0 20 | 34 0 20 | 33 0 1 | 33 0 1 | ? | 32 10 1 | 0 0 20 | 0 -10 4 | 0 0 20 | 0 0 1 | 0 0 1 | 0 0 1 | 0 0 1 | 0 0 20 | 0 0 20 | 0 0 20 | 0 0 20 | 0 0 20 | 0 0 20 | 0 0 20 | 0 0 4 | 0 0 20 |
| lkkd6wwp | 2 | B | **ADS ChM AMP** | 4 0 60 | 4 0 30 | 4 0 60 | 4 0 30 | 4 0 30 | 3 0 30 | 5 0 30 | 5 0 30 | 4 0 20 | 3 0 20 | 5 0 6 | 3 0 6 | 4 0 6 | 3 0 20 | 4 0 20 | 2 0 20 | 4 0 6 | 3 0 6 | 3 0 4 | 3 0 6 | 1 0 1 | 4 0 1 | 8 0 6 | 7 0 4 | 7 0 4 | 3 0 4 | 4 0  5 | 4 0 1 | 4 0  5 | 2 0 4 | 4 0  5 | 5 0  5 | 4 0  5 | 4 0  5 | 4 0  5 | 3 0 1 | 2 0 1 | 3 0 1 | 6 0 1 | 5 0 1 | 3 -10 1 | 3 -10 1 | 3 -10 1 | 3 -10 1 | 4 -10 1 | 5 -10 1 | 4 0 1 | 6 0 1 | 4 0 6 | 4 0 6 | 5 0 1 | 4 0 1 | 4 0 6 | 4 0 6 | 6 0  5 | 4 0 6 | 5 0 1 | 5 0 6 | 5 0 6 | 6 0 6 |
| fdlp6ihd | 2 | B | **ADS ChM AMP** | 11 0 60 | 4 0 60 | 4 0 60 | 22 0 60 | 18 0 60 | 4 0 40 | 4 0 40 | 25 0 40 | 6 0 4 | 2 0  5 | 3 0 30 | 3 0 20 | 8 0 20 | 2 0 30 | 8 0 20 | 11 0 40 | 19 0 20 | 23 0 20 | 3 0 20 | 9 0 20 | 9 0 20 | 5 0 40 | 9 0 40 | 4 0 40 | 16 0 40 | 16 0 4 | 21 0 40 | 20 0 40 | 24 0 20 | 29 0 4 | 21 0 4 | 21 10 20 | 9 0 20 | 4 0 20 | 6 0 40 | 21 0 20 | 22 0 20 | 14 0 20 | 23 0 20 | 7 0 4 | 11 0 4 | 3 0 20 | 8 0 4 | 7 0 4 | 8 0  5 | 7 0 20 | 5 0 20 | 6 0  5 | 15 0  5 | 9 0  5 | 4 0  5 | 7 0  5 | 7 0 1 | 4 0 1 | 5 0 1 | 6 0 1 | 8 0 1 | 5 0 1 | 7 0 1 | 4 0 1 |
| jkld8jrm | 2 | B | **ADS ChM AMP** | 13 0 60 | 15 0 60 | 15 0 30 | 13 0 30 | 23 0 40 | 21 0 20 | 22 0 20 | 21 0 30 | 22 0 20 | 23 0 20 | 23 0 20 | 23 0 4 | 24 0 20 | 25 0 20 | 25 0 20 | 24 0 20 | 24 0 20 | 25 0 20 | 25 0 20 | 25 0 20 | 24 0 20 | 25 0 20 | 26 0 20 | 25 0 20 | 25 0 20 | 25 0 20 | 26 0 20 | 24 0 20 | 24 0 20 | 24 0 20 | 24 0 20 | 24 0 20 | 24 0 20 | 24 0 20 | 24 0 20 | 24 0 20 | 24 0 20 | 24 0 20 | 24 0 20 | 24 0 20 | 24 0 20 | 24 0 20 | 24 0 20 | 24 0 20 | 24 0 20 | 23 0 20 | 24 0 20 | 24 0 20 | 24 0 20 | 24 0 20 | 24 0 20 | 24 0 20 | 24 0 20 | 24 0 20 | 24 0 20 | 24 0 20 | 24 0 20 | 23 0 20 | 23 0 20 | 23 0 20 |
| ytxv6ipg | 2 | B | **ADS ChM AMP** | 14 0 40 | 19 0 60 | 17 0 40 | 13 0 20 | 7 0 20 | 6 0 30 | 7 0 20 | 7 0 20 | 5 0 20 | 1 0 20 | 4 0  5 | 2 0  5 | 4 0  5 | 3 0  5 | 3 0  5 | 3 0  5 | 3 0  5 | 4 0  5 | 3 0  5 | 4 0  5 | 4 0 20 | 4 0  5 | 6 0  5 | 4 0  5 | 4 0  5 | 4 0 10 | 3 0  5 | 5 0  5 | 5 0  5 | 7 0  5 | 5 0  5 | 6 0 10 | 2 0  5 | 2 0  5 | 8 0  5 | 3 0  5 | 11 0 10 | 3 0  5 | 3 0  5 | 3 0  5 | 4 0  5 | 3 0  5 | 3 0  5 | 5 0  5 | 3 0 10 | 3 0  5 | 1 0  5 | 3 0  5 | 6 0  5 | 3 0  5 | 3 0  5 | 3 0  5 | 3 0  5 | 3 0  5 | 3 0  5 | 3 0  5 | 3 0  5 | 3 0  5 | 3 0  5 | 3 0  5 |
| iuau8mnw | 2 | B | **ADS ChM AMP** | 9 10 60 | 2 0 60 | 5 -10 60 | 8 -10 60 | 15 -10 60 | 1 -10 60 | 18 0 60 | 2 0 30 | 4 0 30 | 3 0 20 | 3 0 20 | 4 0 20 | 11 0 20 | 5 0  5 | 3 0 1 | 15 0 30 | 2 0 1 | 2 0 1 | 2 0 1 | 15 0  5 | 0 0  5 | 2 0 1 | 0 0 1 | 0 0 1 | 0 0 1 | 0 0 1 | 2 0  5 | 0 0  5 | 6 0  5 | 5 0  5 | 2 0 20 | 13 0 20 | 12 0  5 | 2 0 1 | 21 0  5 | 15 0  5 | 10 -10  5 | 28 0  5 | 29 0 20 | 13 0 20 | 3 0 20 | 2 0  5 | 0 0  5 | 0 0 1 | 2 0  5 | 2 0  5 | 0 0 20 | 0 0 20 | 0 0 20 | 2 0 20 | 2 0 20 | 22 0 20 | 13 0 20 | 23 0 20 | 9 0 20 | 2 0 20 | 2 0 20 | 2 0 20 | 2 0 20 | 7 0 20 |
| auxv6azb | 2 | B | **ADS ChM AMP** | 12 0 60 | 5 0 60 | 4 0 30 | 6 0 40 | 10 0 60 | 8 0 60 | 14 0 60 | 14 0 60 | 11 0 30 | 17 0 20 | 7 0 40 | 7 0 30 | 7 0 30 | 6 0 30 | 6 0 30 | 7 0 30 | 4 0 30 | 5 0 30 | 8 0 30 | 6 0 30 | 7 0 6 | 9 0 6 | 8 0 30 | 6 0 30 | 9 0 30 | 7 0 6 | 9 0 20 | 6 0 30 | 8 0 6 | 5 0 30 | 5 0 10 | 5 0 10 | 2 0 10 | 4 0 10 | 6 0 20 | 6 0 10 | 5 0 10 | 8 0 10 | 9 0 10 | 7 0 40 | 4 0 40 | 5 0 40 | 6 0 40 | 16 0 10 | 13 0 10 | 1 0 60 | 12 0 40 | 14 0 40 | 10 0 10 | 11 0 10 | 7 0 10 | 10 0 10 | 9 0 10 | 10 0 20 | 11 0 10 | 8 0 60 | 8 0 60 | 7 0 40 | ? | 9 0 40 |
| nuvk8aeg | 2 | B | **ADS ChM AMP** | 22 0 60 | 16 0 60 | 16 0 60 | 14 0 60 | 24 0 60 | 14 0 60 | 10 0 60 | 11 0 40 | 14 0 40 | 17 0 40 | 15 0 20 | 21 0 20 | 14 0 20 | 21 0 20 | 13 0  5 | 8 0  5 | 9 0  5 | 15 0  5 | 12 0  5 | 15 -10  5 | 19 0  5 | 17 -10  5 | 11 0  5 | 12 0  5 | 16 0  5 | 16 0  5 | 11 0  5 | 13 0  5 | 9 0  5 | 10 0  5 | 14 0  5 | 18 0  5 | 13 0  5 | 13 0  5 | 10 0  5 | 9 0  5 | 8 0  5 | 10 -10  5 | 13 0  5 | 10 0  5 | 9 0  5 | 11 0  5 | 13 -10  5 | 18 0  5 | 8 0  5 | 10 0  5 | 12 -10  5 | 15 0  5 | 10 -10  5 | 12 0  5 | 15 -10  5 | 11 0  5 | 17 0  5 | 13 0  5 | 17 0  5 | 26 0  5 | 21 0  5 | 22 0  5 | 19 0  5 | 21 0  5 |
| kdxf8rrl | 2 | B | **ADS ChM AMP** | 11 0 60 | 7 0 60 | 9 0 60 | 11 0 60 | 10 0 60 | 9 0 60 | 9 0 60 | 9 0 30 | 8 0 30 | 9 0 40 | 10 0 20 | 11 0 20 | 10 0 20 | 8 0 20 | 11 0 10 | 14 0 10 | 5 0 30 | 9 0 30 | 8 0 20 | 7 0 20 | 9 0 10 | 12 0 20 | 11 0 20 | 12 0 10 | 11 0 20 | 10 0 40 | 10 0 30 | 9 0 10 | 9 10 10 | 10 0 10 | 9 0 40 | 9 0 20 | 9 0 30 | 10 0 30 | 22 0 30 | 12 0 30 | 10 0 30 | 13 0 30 | 10 0 30 | 9 0 30 | 6 0 30 | 6 -10 30 | 8 0 20 | 8 0 40 | 12 0 40 | 7 0 40 | 6 0 40 | 5 0 40 | 9 0 40 | 6 0 40 | 7 0 40 | 7 0 40 | 10 0 40 | 8 0 40 | 6 0 40 | 7 0 40 | 7 0 40 | 8 0 40 | 10 0 20 | 10 0 20 |
| xpad6okp | 2 | B | **ADS ChM AMP** | 15 0 60 | 12 0 60 | 16 0 60 | 7 0 60 | 14 0 60 | 13 0 60 | 9 0 60 | 5 0 60 | 6 0 60 | 5 0 30 | 6 0 30 | 9 0 60 | 6 0 30 | 7 0 40 | 6 0 40 | 5 0 60 | 5 0 30 | 7 0 40 | 10 0 40 | 11 0 20 | 4 0 60 | 7 0 20 | 5 0 60 | 9 0 60 | 4 0 60 | 4 0 40 | 5 0 30 | 5 0 60 | 13 0 40 | 7 0 20 | 13 0 20 | 2 0 4 | 2 0 20 | 4 0 20 | 5 0 60 | 14 0 60 | 9 0 60 | 11 0 30 | 10 0 20 | 14 0 20 | 8 0 20 | 12 0 20 | 16 0 40 | 10 0 1 | 5 0 1 | 5 0 1 | 3 0 1 | 2 0 1 | 7 0 4 | 8 0 20 | 9 0 20 | 8 0 20 | 8 0 20 | 15 0 4 | 9 0 1 | 2 0 1 | 13 0 20 | 14 0 40 | 11 0 20 | 7 0 10 |
| tkyd6dsg | 2 | B | **ADS ChM AMP** | 29 0 30 | 20 0 60 | 14 0 40 | 7 0 60 | 9 0 40 | 5 0 60 | 7 0 60 | 8 0 40 | 5 0 40 | 6 0 60 | 9 0 40 | 7 0 40 | 6 0 40 | 3 0 60 | 8 0 10 | 4 0 60 | 5 0 40 | 6 0 40 | 0 0 60 | 2 0 40 | 2 0 60 | 0 0 60 | 1 0 60 | 2 0 60 | 2 0 60 | 5 0 40 | 14 0 40 | 15 0 10 | 13 0 10 | 12 0 10 | 9 0 10 | 16 0 10 | 11 0  5 | 3 0 40 | 2 0 60 | 6 0 60 | 4 0 10 | 4 0 10 | 8 0 1 | 21 0 1 | 17 0 1 | 12 0  5 | 21 0 1 | 19 0 1 | 25 0 1 | 19 0  5 | 10 0 10 | 22 0 1 | 21 0  5 | 13 0 10 | 13 0 10 | 9 0  5 | 12 0  5 | 8 0 10 | 16 0  5 | 14 0 10 | 9 0  5 | 12 0 10 | 12 0  5 | 17 0  5 |
| uhjd2sed | 2 | D | **ADS ChM AMP** | 13 0 30 | 10 0 60 | 13 0 4 | 9 0 4 | 11 0  5 | 6 0 20 | 5 0 20 | 9 0 20 | 9 0 20 | 15 0 20 | 10 0 20 | 4 0 1 | 14 0 4 | 11 0 4 | 9 0 20 | 22 0 20 | 15 0 20 | 7 0 30 | 3 0 4 | 5 0 1 | 11 0 4 | 8 0 20 | 7 0 20 | 5 0 4 | 4 0 4 | 4 0  5 | 5 0 1 | 3 0  5 | 4 0 6 | 3 0 4 | 4 0 20 | 4 0 20 | 4 0 6 | 5 0 20 | 4 0 4 | 4 0 4 | 8 0 4 | 5 0 4 | 5 0 1 | 5 0 4 | 5 0 4 | ? | 7 0 1 | 4 0 6 | 3 0 30 | 4 0 6 | 7 0 1 | ? | 11 0 30 | 4 0 1 | 5 0 1 | 5 0  5 | 2 0 4 | 4 0 4 | 7 0 1 | 9 0  5 | 4 0 4 | 5 0 4 | 9 0  5 | 5 0 1 |
| uhat8lkg | 2 | D | **ADS ChM AMP** | 25 -10 6 | 15 0 1 | 24 0 1 | 28 0 1 | 21 0 1 | 17 0 1 | 22 0 1 | 12 10 1 | 9 10 1 | 11 0 1 | 9 0 1 | ? | ? | 5 0 1 | 8 0 1 | 2 0 40 | 2 0 40 | 14 0 4 | 11 0  5 | 5 -10  5 | 9 0  5 | 8 0 10 | 8 0 10 | 12 1  5 | 15 -10  5 | 11 0  5 | 16 1 1 | 27 0  5 | 15 0 1 | 12 10 1 | 17 0 1 | 13 0 4 | 12 0 1 | 9 0  5 | 23 0  5 | 19 0 1 | 11 0 1 | 18 10 1 | 26 0 1 | 30 -10 1 | 29 0 1 | 29 -10 1 | 9 10  5 | 12 10 10 | 9 -10 40 | 9 0  5 | 11 0 1 | 25 0 1 | 27 0 1 | 27 0 1 | 17 0  5 | 26 0  5 | 42 10 40 | 22 10  5 | 12 0  5 | 26 0 1 | 17 10 1 | 21 10 1 | 27 0  5 | 25 1  5 |
| lpxf4bzn | 2 | D | **ADS ChM AMP** | 7 0 30 | 7 0 60 | 7 0 20 | 7 0 40 | 8 0 20 | 13 0 30 | 14 0 30 | 8 0 20 | 10 0  5 | 8 0 20 | 12 0 20 | 15 0  5 | 9 0 20 | 7 0 20 | 7 0 20 | 5 0  5 | 5 0  5 | 10 0 20 | 7 0 20 | 7 0  5 | 6 0 10 | 10 0 20 | 9 0 40 | 9 0 40 | 11 0 20 | 14 0 10 | 17 10 10 | 16 10 40 | 13 0 10 | 12 0 20 | 13 0 40 | 6 10 20 | 5 0 10 | 7 0  5 | 10 0 20 | 8 0 20 | 6 0 10 | 6 0 10 | 7 0 10 | 8 0 10 | 7 0 10 | 8 0 40 | 18 0 20 | 7 0  5 | 9 0 40 | 22 0 10 | 20 0 40 | 11 0 40 | 8 0 40 | 9 0 20 | 10 0 40 | 15 0  5 | 13 0 40 | 12 0 20 | 13 0 10 | 12 0 10 | 12 0 10 | 10 0 40 | 15 0 10 | 14 0 10 |
| fiah4ndi | 2 | D | **ADS ChM AMP** | 15 0 60 | 14 0 60 | 9 0 60 | 7 0 60 | 3 0 60 | 8 0 30 | 5 0 30 | 5 0 30 | 8 0 30 | 8 0 20 | 10 0 30 | 15 0 30 | 9 0 30 | 6 0 20 | 9 0 20 | 8 0 30 | 7 0 30 | 6 0 30 | 9 0 30 | 7 0 30 | 6 0 30 | 5 0 30 | 3 0 6 | 5 0 6 | 7 0 6 | 10 0 6 | 7 0 30 | 5 0 30 | 10 0 30 | 9 0 30 | 9 0 30 | 15 0 30 | 12 0 30 | 7 0 4 | 10 0 4 | 4 0 30 | 7 0 30 | 7 0 30 | 11 0 30 | 11 0 30 | 7 0 60 | 7 0 30 | 8 0 6 | 14 0 4 | 9 0 20 | 7 0 30 | 6 0 20 | 6 0 30 | 6 0 30 | 5 0 30 | 5 0 30 | 7 0 30 | 12 0 6 | 6 0 30 | 5 0 6 | 4 0 30 | 3 0 30 | 5 0 20 | 8 0 20 | 6 0 20 |
| road6wfm | 2 | D | **ADS ChM AMP** | 29 0 20 | 33 0 60 | 20 0 60 | 20 0 60 | 4 0 60 | 3 0 60 | 2 0 60 | 1 0 40 | 4 0 20 | 8 0 40 | 5 0 20 | 2 0 20 | 3 0 20 | 0 0 20 | 1 0 20 | 3 0  5 | 9 0  5 | 9 0  5 | 4 0 20 | 5 0  5 | 7 0 10 | 6 0 20 | 3 0 20 | 4 0 20 | 2 0 20 | 2 0 20 | 8 0  5 | 11 0  5 | 10 0  5 | ? | 9 0 4 | ? | 9 0 4 | 13 0 20 | 7 0 20 | 3 0 20 | ? | ? | ? | ? | ? | ? | ? | ? | ? | ? | ? | ? | ? | ? | ? | ? | ? | ? | ? | ? | ? | ? | ? | ? |
| phfk6wdj | 2 | D | **ADS ChM AMP** | 7 0 6 | 8 0 20 | 7 0 20 | 12 0 30 | 12 0 40 | 8 0 20 | 10 0 30 | 10 0 20 | 9 0 20 | ? | 5 0 10 | 5 0  5 | 9 0  5 | 6 0 20 | 15 0 20 | 9 0  5 | 11 0 4 | 15 0  5 | ? | 13 0 20 | 5 0 10 | ? | 12 0 4 | ? | ? | ? | 13 0  5 | 13 0 20 | ? | ? | ? | 11 0 4 | 8 0 4 | 12 0 4 | ? | ? | ? | ? | ? | ? | ? | ? | ? | ? | ? | ? | ? | ? | ? | ? | ? | ? | ? | ? | ? | ? | ? | ? | ? | ? |
| phnd5fdf | 2 | D | **ADS ChM AMP** | 1 0 30 | 0 0 60 | 0 0 20 | 0 0 20 | 1 0 20 | 1 0 4 | 0 0  5 | 0 0 4 | 1 0 1 | ? | 1 0 20 | 1 0 6 | 0 0 4 | 1 0 4 | 0 0 4 | 0 0 6 | 0 0 4 | 0 0 1 | 1 0 4 | 0 0 1 | 0 0 1 | 0 0 1 | 0 0 1 | 0 0 1 | 0 0 4 | 0 0 1 | 0 0 4 | 0 0 20 | 0 0 20 | 0 0 20 | 1 0 6 | 1 0 4 | 0 -10 4 | 3 0 1 | 0 0 4 | 1 0 1 | 1 0 1 | 0 0 4 | 0 0 1 | 0 0 1 | 0 0 1 | 1 0 4 | 1 0 4 | 0 0 1 | 0 0 4 | 0 0 1 | 0 0 1 | 1 0 1 | 0 0 1 | 0 0 1 | 0 0 1 | 0 0 1 | 0 0 1 | 0 0 1 | 0 0 1 | 0 0 1 | 0 0 1 | 1 0 1 | 1 0 1 | 0 0 1 |
| brrw5xda | 2 | D | **ADS ChM AMP** | 1 0 30 | 1 0 60 | 3 0 30 | 3 0 20 | 0 0 10 | 4 0 10 | 8 0 1 | 1 0 1 | 3 0 4 | 0 0 1 | 0 0 1 | 2 0 1 | 0 0 1 | 5 0 1 | 6 0 1 | 4 -10 1 | 7 0 1 | 2 0 1 | 3 0 1 | 3 0 1 | 7 -10 1 | 4 0 1 | 2 0 1 | 0 0 1 | 1 0 1 | 4 0 1 | 2 0 1 | 2 0 1 | 1 0 1 | 0 0 1 | 2 0 1 | 0 0 1 | 1 0 1 | 0 -10 1 | 0 0 1 | 3 0 1 | 5 0 1 | 10 0 1 | 8 0 1 | 5 0 1 | 1 0 1 | 5 0 1 | 3 0 1 | 14 0 1 | 7 0 1 | 2 0 1 | 7 0 1 | 2 0 1 | 2 0 1 | 3 0 1 | 0 0 1 | 0 0 1 | 2 0 1 | 1 0 1 | 6 0 1 | 2 0 1 | 1 0 1 | 5 0 1 | 1 0 1 | 0 0 1 |
| ndxf6dvi | 2 | D | **ADS ChM AMP** | 16 0 20 | 18 0 30 | 22 0 30 | 11 0 30 | 13 0 60 | 12 0 30 | 28 0 60 | 24 1 30 | 6 0 60 | 5 0 60 | 5 0 30 | 10 0 30 | 8 0 60 | 5 0 30 | 8 0 60 | 14 0 30 | 12 0 30 | 15 0 20 | 6 0 40 | 7 0 40 | 5 0 30 | 10 0 40 | 7 0 30 | 7 0 40 | 8 0 40 | 6 0 40 | 13 0 20 | 7 0 30 | 7 0 30 | 7 0 30 | 16 0 20 | 19 0 30 | 6 0 40 | 11 0 30 | 20 0 40 | 8 0 10 | 4 0 40 | 4 0 10 | 7 0 30 | 9 0 40 | 6 0 40 | 7 0 40 | 7 0 40 | 7 0 10 | 6 0 40 | 8 0 40 | 13 0 40 | 6 0 40 | 12 0 40 | 11 0 30 | 11 0 30 | 9 0 30 | 8 0 30 | 16 0 30 | 13 0 40 | 10 0 10 | 10 0 40 | 15 0 40 | 15 0 40 | 7 0 10 |
| kdir4wwb | 2 | D | **ADS ChM AMP** | 7 0 30 | 5 0 20 | 16 0 20 | 25 0 20 | 24 0 20 | 6 0  5 | 10 0 20 | 19 0 20 | 23 0  5 | 19 0  5 | 14 0  5 | 6 0  5 | 7 0  5 | 15 0  5 | 26 0  5 | 19 0  5 | 23 0 4 | 22 0 4 | 24 0  5 | 17 0  5 | 10 0 1 | 9 0 1 | 8 0 1 | 12 0 1 | 8 0 1 | 14 0 1 | 10 0 1 | 10 0 1 | 19 0 1 | 10 0 1 | 15 0 1 | 12 0 1 | 12 0 1 | 11 0 1 | 8 0 1 | 8 0 1 | 6 0 1 | 16 0 1 | 12 0 1 | 11 0 1 | 23 0 1 | 12 0 1 | 15 0 1 | 12 0 1 | 12 0 1 | 19 0 1 | 20 0 1 | 22 0 1 | 22 0 1 | 16 0 1 | 16 0 1 | 24 0 1 | 20 0 1 | 22 0 1 | 22 0 1 | 20 0 1 | 24 0 1 | 21 0 1 | 20 0 1 | 15 0 1 |
| ktlk4hll | 2 | D | **ADS ChM AMP** | 23 10 60 | 27 10 60 | 25 0 60 | 6 0 60 | 13 0 60 | 13 10 60 | 4 0 60 | 26 0 60 | 2 0 30 | 7 0 30 | 4 0 40 | 4 0 10 | 16 0 10 | 13 0 10 | 12 0 20 | 14 0 20 | 11 0 40 | 13 0 40 | 4 0 10 | 5 0  5 | 5 0 20 | 2 0 10 | 4 0 10 | 5 0 40 | 3 0 40 | 8 0 10 | 8 0 10 | 17 0 10 | 16 0 10 | 11 0 40 | 3 0 40 | 4 0 40 | 2 0 40 | 4 0 40 | 11 0 10 | 9 0 40 | 9 0 10 | 12 0 10 | 7 0 10 | 5 0 10 | 2 0 40 | 2 0 10 | 2 0 10 | 2 0 10 | 6 0 10 | 4 0  5 | 9 0 10 | 3 0 10 | 7 0 10 | 4 0 10 | 2 -10 10 | 13 0 10 | 18 0 10 | 11 0 10 | 12 0 10 | 6 0 40 | 21 0 10 | 4 0 10 | 13 0 10 | 8 0 20 |
| xulu4idp | 2 | D | **ADS ChM AMP** | 10 0 10 | 12 0 10 | 11 0 60 | 9 0 20 | 16 0 20 | 18 0 20 | 14 0 40 | 20 0 20 | 13 0 10 | 18 0 10 | 11 0  5 | 13 0 10 | 13 -10  5 | 14 -10  5 | 12 -10  5 | 11 -10  5 | 13 -10  5 | 11 -10 10 | 12 -10  5 | 12 -10  5 | 12 -10  5 | 13 -10  5 | 12 -10  5 | 11 -10 1 | 14 -10 1 | 11 -10 1 | 12 -10 1 | 16 -10 1 | ? | 12 -10 1 | 13 -10 4 | 19 -10 20 | 12 -10  5 | 13 0 20 | 11 0 20 | 13 0  5 | 12 -10  5 | 15 -10 20 | 11 -10  5 | 15 -10 20 | 15 -10  5 | 12 -10 20 | 13 -10 20 | 9 -10  5 | 10 -10  5 | 11 -10  5 | 10 -10 1 | 9 -10 1 | 12 -10 1 | 11 -10 1 | 11 -10 1 | 10 -10  5 | 10 -10  5 | 14 -10 1 | 14 -10 1 | 14 -10  5 | 12 -10  5 | 13 -10  5 | 8 -10 1 | 13 -10 1 |
| ndjp3tmw | 2 | E | **ADS ChM AMP** | 18 0 10 | 13 0 60 | 14 0 6 | 13 0 60 | 14 0 6 | 11 0 30 | 14 0 6 | 15 0 1 | 11 0 1 | ? | 15 0 1 | 13 0 1 | 13 0 1 | 13 0 1 | 10 0 1 | 12 0 1 | 15 0 1 | 11 0 1 | 10 0 1 | 13 0 1 | 17 0 1 | 16 0 1 | 10 0 1 | 11 0 1 | 9 0 1 | 12 0 1 | 10 0 1 | 11 0 1 | ? | 15 0 1 | 11 0 1 | 15 0 1 | 12 0 1 | 12 0 1 | 17 0 1 | ? | 12 0  5 | 11 0 4 | 16 0 1 | 14 0 1 | 11 0 4 | 12 0 4 | 13 0 40 | 12 0 20 | 14 0 1 | 19 0 1 | 11 0 20 | 13 0 40 | 18 0 4 | 16 0 4 | 11 0 40 | 14 0 4 | 14 0 4 | 13 0 4 | 22 0 4 | 14 0 1 | 13 0 1 | 15 0 1 | 19 0 1 | 17 0 4 |
| uwat4xjj | 2 | E | **ADS ChM AMP** | 0 0 30 | 0 0 60 | 0 0 60 | 0 0 30 | 0 0 30 | 0 0 30 | 0 0 30 | 1 0 20 | 1 0 20 | 0 0 30 | 0 0  5 | 0 0 20 | 0 0  5 | 0 0 1 | 0 0 1 | 0 0 1 | 0 0 1 | 0 0 1 | 0 0 1 | 0 0  5 | 0 0  5 | 1 0  5 | 1 0  5 | 0 0 1 | 0 0 1 | 0 0  5 | 0 0  5 | 0 0 1 | 0 0 1 | 0 0 1 | 0 0  5 | 0 0 1 | 0 0 1 | 0 0 1 | 0 0 1 | 6 0 1 | 0 0 1 | 3 0 1 | 0 0 1 | 0 0  5 | 0 0 1 | 0 0 1 | 0 0 1 | 0 0 1 | 0 0 1 | 0 0 1 | 0 0  5 | 0 0 1 | 0 0 1 | 0 0 1 | 0 0 1 | 0 0  5 | 0 0  5 | 0 0 1 | 0 0 1 | 11 0 1 | 7 0 1 | 12 0 1 | 13 10 1 | 12 10 1 |
| lrsd5ztl | 2 | E | **ADS ChM AMP** | 5 0 60 | 3 0 10 | 6 0 6 | 9 0 20 | 6 0  5 | 5 0 10 | 3 0 20 | 10 0 20 | 9 0  5 | 5 0 20 | 2 0 40 | 8 0 20 | 10 0 10 | 8 0 10 | 5 0 40 | 3 0  5 | 7 0 40 | 2 0 1 | 6 0 20 | 7 0 1 | 6 0 10 | 15 0 40 | 6 0 20 | 4 0 1 | 8 0 1 | 10 0 10 | 7 0  5 | 13 0 10 | 9 0 10 | 5 0  5 | 3 0 40 | 5 0 40 | 10 0  5 | 15 0 20 | 12 0 40 | 6 0 20 | 10 0 20 | 8 0 20 | 11 0 40 | 9 0 1 | 7 0 20 | 4 0 20 | 7 0 1 | 6 0 1 | 7 0 20 | 3 0 10 | 6 0 20 | 6 0 20 | 5 0 4 | 6 0 40 | 5 0  5 | 8 0 1 | 7 0 1 | 3 0 40 | 3 0 40 | 3 0 4 | 10 0 1 | 5 0 1 | 5 0 1 | 11 0 40 |
| lrdx5iiy | 2 | E | **ADS ChM AMP** | 13 0 60 | 24 0 60 | 15 0 60 | 17 0 60 | 25 0 60 | 13 0 60 | 8 0 60 | 16 0 60 | 16 0 10 | 13 0 1 | 13 0 1 | 20 0 1 | 21 0 1 | 10 0 1 | 7 -10 1 | 7 -10 1 | ? | 25 0 1 | 14 0 1 | 23 0 1 | 18 0 1 | ? | ? | 12 0 1 | 10 0 1 | 9 0 1 | 13 0 1 | ? | 14 0 1 | 15 0 1 | 15 0 1 | 17 0 1 | ? | ? | ? | ? | ? | ? | ? | ? | ? | ? | ? | ? | ? | ? | ? | ? | ? | ? | ? | ? | ? | ? | ? | ? | ? | ? | ? | ? |
| lklu4jal | 2 | E | **ADS ChM AMP** | 9 0 60 | 13 0 30 | 4 0 30 | 21 0 60 | 26 0 30 | 11 0 40 | 13 0 20 | 3 0 1 | 6 0 4 | 6 0 20 | 19 0 20 | 9 0 20 | 6 0 20 | 6 0 20 | 8 0 1 | 6 0 1 | 13 0 1 | 22 0 20 | 21 0 20 | 19 0 20 | 10 0 4 | 2 0 1 | 12 0 1 | 10 0 20 | 11 0 20 | 8 0 20 | 5 0 20 | 10 0 4 | 2 0 20 | 23 0 20 | 25 0 20 | 22 0 4 | 35 0 20 | 28 0 20 | 27 0 20 | 30 0 20 | 30 0 4 | 22 0 20 | 22 -10 20 | 22 0 20 | 30 0 1 | 14 0 4 | 18 0 20 | 20 0 20 | 17 0 4 | 11 0 20 | 5 0 20 | 11 0 20 | 17 0 20 | 22 0 20 | 15 0 20 | 14 0 20 | 20 0 20 | 18 0 4 | 30 10 4 | 17 10 20 | 21 10 4 | 16 0 20 | 17 0 4 | 29 0 4 |
| road4jyg | 2 | E | **ADS ChM AMP** | 15 0 60 | 7 0 60 | 9 0 60 | 11 0 30 | 8 0 60 | 23 0 60 | 18 0 30 | 13 0 40 | 9 0 40 | 9 0 20 | 6 0 20 | 6 0 40 | 16 0 20 | 13 0 20 | 9 0  5 | 11 0 20 | 15 0 20 | 10 0 20 | 11 0 20 | 12 0 20 | 6 0 20 | 15 0  5 | 23 0 20 | 23 0 1 | 24 0 1 | 21 0 20 | 21 0  5 | 17 0  5 | 23 0  5 | 17 0 10 | 13 0 20 | 18 0 20 | 5 0 40 | 8 0 40 | 15 0 20 | 19 0 20 | 16 0 20 | 23 0 20 | 20 0 10 | 25 0 1 | 20 0 4 | 19 0 20 | 22 0 20 | 18 0 20 | 19 0 10 | 14 0 20 | 15 0 10 | 10 0 20 | 21 0  5 | 14 0 10 | 14 0 20 | 9 0 20 | 6 0  5 | 7 0 40 | 4 0 10 | 7 0 40 | 11 0 4 | 11 0 10 | 19 0 10 | 5 0 10 |
| jufi5igb | 2 | E | **ADS ChM AMP** | 8 0 60 | 7 0 60 | 8 0 40 | 31 0 40 | 22 0 40 | 30 0 60 | 29 0 60 | 12 0 60 | 8 0 60 | 5 0 60 | 3 0 60 | 2 0 60 | 6 0 30 | 21 0 30 | 23 0 60 | 27 0 60 | 31 0 60 | 20 0 60 | 25 0 60 | 32 0 60 | 34 0 60 | 38 10 20 | 28 0 6 | 33 0 1 | 24 0 1 | 22 0 30 | 17 0 30 | 18 0 30 | 22 0 30 | 30 10 6 | 5 -10 6 | 11 0 1 | 10 0 1 | 13 0 1 | 25 0 6 | 22 0 1 | 29 0 1 | 17 0 4 | 19 10 1 | 20 0 1 | 24 0 1 | 15 0 1 | 14 0 1 | 12 0 1 | 8 0 1 | 6 0 1 | 2 0 1 | 6 0 1 | 7 0 1 | 10 -10 6 | 8 0 1 | 15 0 1 | 5 0 1 | 9 0 1 | 22 0 1 | 11 0 1 | 12 0 1 | 9 0 1 | 13 0 1 | 26 -10 1 |
| irhd4uip | 2 | E | **ADS ChM AMP** | 17 0 30 | 25 0 30 | 16 0 30 | 15 0  5 | 11 0  5 | 9 0 20 | 14 0 20 | 14 0 1 | 10 0 4 | 12 0 1 | 16 0 4 | 14 0 1 | 21 0 1 | 32 10 1 | 18 10 1 | 17 0 1 | 17 0 1 | 12 0 1 | 20 0 1 | 11 0 1 | 12 0 1 | 10 10 1 | 11 0 1 | 9 0 1 | 12 0 1 | 15 0 1 | 7 0 1 | 4 0 1 | 9 0 1 | 6 0 1 | 4 0 1 | 4 0 1 | 7 0 1 | 9 0 1 | 21 0 1 | 5 0 1 | 8 0 1 | 7 0 1 | 4 0 1 | 6 0 4 | 12 0 1 | 7 0 1 | 14 0 1 | 13 0  5 | 8 0 1 | 12 0 10 | 16 0 1 | 12 0 1 | 9 -10 1 | 5 -10 1 | 7 0 1 | 16 0 1 | 10 0 1 | 11 0 1 | 11 0 1 | 10 0 1 | 8 0 1 | 10 0 1 | 10 0 1 | 5 0 1 |
| bruk4khl | 2 | E | **ADS ChM AMP** | 9 0 40 | 4 0 60 | 7 0 30 | 9 0 60 | 4 0 60 | 9 0 60 | 3 0 40 | 4 0 40 | 4 10 20 | 3 0 20 | 1 0 40 | 3 0 20 | 8 0 20 | 5 0 10 | 0 0 40 | 3 0  5 | 6 0  5 | 8 0  5 | 3 0 10 | 3 0 10 | 1 0 40 | 2 0 10 | 6 0 20 | 2 0  5 | 3 0  5 | 5 0  5 | 4 0  5 | 3 0 10 | 3 0 10 | 3 0 10 | 0 0 40 | 3 0  5 | 2 0 10 | 4 0  5 | 2 0  5 | 3 0  5 | 0 0 10 | 0 0  5 | 3 0  5 | 3 0 1 | 7 0  5 | 3 0  5 | 4 0 20 | 2 0  5 | 2 0  5 | 3 0 1 | 2 0 10 | 1 0 10 | 0 0 10 | 7 0 1 | 2 0 10 | 1 0 10 | 1 0 40 | 2 0 10 | 1 0 10 | 1 0 10 | 1 0 10 | 5 0 1 | 8 0 1 | 4 0  5 |
| nupo2syr | 2 | E | **ADS ChM AMP** | 10 0 30 | 12 0 60 | 19 0 20 | 13 0 60 | 10 0 60 | 10 0 60 | 14 0 30 | 10 0 6 | 12 0 4 | 9 0 6 | 10 0 6 | 9 0 6 | 8 0 1 | 10 0 1 | 10 0 1 | 9 0 6 | 10 0 4 | 18 0 1 | 9 0 1 | 10 0 1 | 12 0 1 | 9 0 1 | 14 0 1 | 9 0 1 | 10 0 4 | 9 0 1 | 9 0 1 | 11 0 1 | 10 0 1 | 11 0 1 | 10 0 1 | 10 0 1 | 14 0 1 | 12 0 1 | 10 0 1 | 12 0 1 | 11 0 1 | 11 0 1 | 14 0 1 | 14 0 1 | 12 0 1 | 14 0 1 | 17 0 1 | 15 0 1 | 18 0 1 | 11 0 6 | 10 0 6 | 9 0 6 | 15 0 1 | 12 0 1 | 15 0 1 | 14 -10 1 | 13 0 1 | 15 0 1 | 12 0 1 | 12 0 1 | 17 0 1 | 13 0 1 | 12 0 1 | 17 0 1 |
| kuat2czi | 2 | E | **ADS ChM AMP** | 4 0 30 | 6 0 60 | 7 0 60 | 10 0 40 | 9 0 40 | 20 0 40 | 21 0 40 | 19 0 10 | 24 0 40 | 21 0 10 | 17 0 10 | 23 0 10 | 29 0 10 | 30 0 10 | 29 0 10 | 34 0 10 | 32 0 10 | 33 0 10 | 29 0 10 | 34 0 10 | 36 0 10 | 32 0 10 | 34 0 10 | 40 0 10 | 39 0 10 | 41 0 10 | 39 0  5 | 42 0  5 | 42 0  5 | 44 0 1 | 45 0 1 | 45 0 1 | 45 0 1 | 45 0 1 | 45 0 1 | 45 0 1 | 45 0 1 | 45 0 1 | 45 0 1 | 45 0 1 | 45 0 1 | 45 0 1 | 45 0 1 | 45 0 1 | 45 0 1 | 45 0 1 | 45 0 1 | 45 0 1 | 22 0 1 | 30 0 1 | 23 0 1 | 22 0 1 | 34 0 1 | 27 0 1 | 35 0 1 | 32 0 1 | 28 0 1 | 32 0 1 | 30 0 1 | 30 0 1 |
| gpfk5bgr | 2 | F | **ADS ChM AMP** | 10 10 6 | 10 -10 30 | 2 0 30 | 22 0 6 | 5 0 30 | 4 0 6 | 4 0 6 | 1 0 6 | 4 0 1 | 5 0 1 | 2 0 1 | 3 0 1 | 8 0 1 | 21 0 20 | 9 0 1 | 13 0 1 | 9 0 1 | 5 0 1 | 3 0 1 | 6 0 1 | 3 0 1 | 5 0 1 | 7 0 1 | 3 0 1 | 9 0 4 | 11 0 4 | 14 0 1 | 15 0 1 | 9 0 1 | 16 0 1 | 15 0 1 | 8 0 1 | 3 0 1 | 5 0 1 | 7 0 1 | 8 0 1 | 7 0 1 | 11 0 1 | ? | 7 0 1 | 7 0 1 | 8 0 1 | 14 0 1 | 5 0 1 | 5 0 1 | 4 0 1 | 7 0 1 | 5 0 1 | 9 0 1 | 4 0 1 | 5 0 1 | 4 0 1 | 5 0 1 | 9 0 1 | 23 0 1 | 15 0 1 | 23 0 1 | 9 0 1 | 8 -10 1 | 4 -10 1 |
| yuet4mbh | 2 | F | **ADS ChM AMP** | 9 0 60 | 5 0 60 | 8 0 30 | 11 0 30 | 11 0 30 | 16 0 60 | 8 0 30 | 10 0 30 | 9 0 4 | 9 0 4 | 15 0 4 | 13 0 4 | 21 0 20 | 8 0 1 | 14 0 1 | 11 0 4 | 8 0 4 | 7 0 4 | 6 0 4 | 19 0 4 | 7 0 6 | 19 0 4 | 14 0 4 | 6 0 4 | 7 0 6 | 5 0 4 | 7 0 6 | 11 0 6 | 9 0 6 | 8 0 6 | 16 0 6 | 10 0 6 | 7 0 4 | 18 0 4 | 12 0 6 | 8 0 6 | 11 0 4 | 21 0 4 | 14 0 4 | 7 0 6 | 7 0 6 | 19 0 20 | 9 0 4 | 14 0 6 | 8 0 6 | 10 0 6 | 20 0 6 | 13 0 6 | 8 0 6 | 8 0 6 | 8 0 6 | 10 0 6 | 6 0 6 | 12 0 6 | 8 0 6 | 15 0 6 | 12 0 6 | 9 0 6 | 16 0 6 | 16 0 6 |
| irlk2mdn | 2 | F | **ADS ChM AMP** | ? | 16 0 60 | 14 0 60 | 17 0 60 | 19 0 60 | 18 0 60 | 17 0 60 | 13 0 10 | 23 0 20 | 16 0 40 | 16 0 10 | 10 0 40 | 12 0 40 | 12 0 40 | 14 0 40 | 10 0 40 | 14 0 40 | 13 0 40 | 12 0 40 | 13 0 20 | 14 0 40 | 11 0 10 | 13 0 20 | 12 0  5 | 14 0 40 | 10 0 20 | 10 0 1 | 15 0  5 | 13 0  5 | 16 0 1 | 13 0 20 | 13 0 20 | 12 0 1 | 14 0  5 | 10 0 1 | 12 0 1 | 15 0 4 | 14 0 60 | 12 0 40 | 15 0 40 | 13 0 60 | 10 0 40 | 12 0 60 | 14 0 40 | 12 0 40 | 13 0 10 | 12 0 60 | 12 0 60 | 11 0 60 | 11 0 30 | 21 0 30 | 18 0 30 | 17 0 20 | 24 0 40 | 17 0 60 | 14 0 40 | 17 0 1 | 18 0 1 | 11 0 40 | 14 0 40 |
| pwlr4ptg | 2 | F | **ADS ChM AMP** | 20 0 60 | 11 0 60 | 1 0 30 | 13 0 20 | 22 0 20 | 41 0 60 | 16 0 60 | 18 0 30 | 17 0 6 | 29 0 1 | 24 0 6 | 10 0 20 | 20 0 20 | 5 0 1 | 1 0 1 | 0 0 1 | 7 0 1 | 1 0 1 | 2 0 1 | 4 0 1 | 3 0 1 | 6 0 1 | 5 0 30 | 3 0 60 | 5 0 60 | 12 0 30 | 9 0 6 | 13 0 6 | 10 0 6 | 13 0 4 | 28 0 6 | 27 0 20 | 19 0 30 | 15 0 30 | 13 0 20 | 9 0 6 | 15 0 4 | 17 0 4 | 1 0 1 | 10 0 6 | 13 0 6 | 6 0 6 | 23 0 6 | 40 0 1 | 30 0 1 | 33 0 1 | 21 0 6 | 25 0 6 | 16 0 6 | 12 0 6 | 6 0 30 | 8 0 30 | 9 0 60 | 15 0 6 | 16 0 6 | 14 0 6 | 15 0 6 | 21 0 6 | 20 0 30 | 16 0 6 |
| bdxf2hej | 2 | F | **ADS ChM AMP** | 3 0 60 | 18 0 60 | 18 0 60 | 2 0 60 | 18 0 60 | 6 0 60 | 2 0 60 | 2 0 40 | 8 0 40 | 14 0 40 | 15 0 40 | 16 0 40 | 9 -10 20 | 14 10 40 | 23 0 20 | 7 0 40 | 3 0 40 | 6 0 40 | 2 -10 40 | 3 0 20 | 4 0 20 | 4 0 20 | 11 0 20 | 4 0  5 | 3 0 20 | 6 0 10 | 18 0 10 | 15 0 10 | 5 0 10 | 3 0  5 | 2 0  5 | 2 0  5 | 2 0  5 | 2 0  5 | 8 0 10 | 12 0 10 | ? | 10 0  5 | 2 0 10 | 12 0  5 | 4 0  5 | 4 0 10 | 11 0 10 | 3 0 10 | 3 0 10 | 8 0  5 | 7 0  5 | 11 0  5 | 15 0 10 | 3 0 20 | 9 0 10 | 2 0  5 | 2 0  5 | 3 0  5 | 8 -10 10 | 3 0 10 | 9 0 10 | 9 0  5 | 4 0  5 | 10 0 20 |
| nufi3btj | 2 | F | **ADS ChM AMP** | 17 0 60 | 15 0 60 | 18 10 60 | 13 0 60 | 13 0 60 | 14 0 60 | 20 0 60 | 17 0 60 | 23 0 60 | 7 0 60 | 26 0 30 | 12 -10 40 | 15 0 60 | 11 0 40 | 5 0 60 | 7 0 60 | 7 0 60 | 6 0 60 | 10 0 60 | 8 0 60 | 8 0 20 | 7 0 40 | 8 0 30 | 6 0 60 | 14 0 40 | 13 0 40 | 19 0 30 | 19 0 6 | 12 0 20 | 25 0 20 | 26 0 6 | 18 0 1 | 13 0 1 | 14 0 4 | 21 0 6 | 12 0 20 | 14 0 40 | 16 0 40 | 24 0 40 | 20 0 4 | 26 0 4 | 27 0 6 | 21 0 20 | 25 0 4 | 23 0 20 | 22 0 20 | 30 0 20 | 23 0 20 | 29 0 20 | 20 0 40 | 14 0 20 | 14 0 20 | 13 0 20 | 20 0 20 | 15 0 20 | 8 0 40 | 24 0 20 | 16 0 20 | 20 0 20 | 14 0 40 |
| xpxv4wxi | 2 | F | **ADS ChM AMP** | ? | 9 0 40 | 11 -10 60 | 15 -10 40 | 10 0 30 | 11 0 30 | 19 0 30 | 18 0 40 | 17 0  5 | 15 0 20 | 14 0  5 | 11 0 30 | 8 0 40 | 15 0 40 | 15 0 1 | 12 0  5 | 20 0 1 | 18 0  5 | 20 0 4 | 18 0 6 | 14 0 4 | 17 0 4 | 13 0  5 | 11 0  5 | 13 0 1 | 11 -10 20 | 16 0  5 | 17 0  5 | 20 0 1 | 13 0 1 | 12 0 20 | 11 0 1 | 16 0  5 | 16 0 1 | 22 0 1 | 23 0 1 | 20 0 10 | 15 0  5 | 15 0 20 | 18 0  5 | 11 0 4 | 14 0 1 | 12 0 1 | 13 0 1 | 14 0 1 | 16 0  5 | 15 0 4 | 9 0 4 | 11 0 4 | 15 0  5 | 18 0 1 | 12 0 1 | 9 0 1 | 18 0  5 | 14 0  5 | 17 0 1 | 14 0 10 | 15 0  5 | 10 0 1 | 10 0  5 |
| tgkr2xpi | 2 | F | **ADS ChM AMP** | 24 0 30 | 22 0 20 | 12 0 20 | 15 0 30 | 17 0 40 | 11 0 30 | 17 0 40 | 17 0  5 | 24 0  5 | 27 0 20 | 24 0 20 | 23 0 20 | 24 0 20 | 18 0 40 | 20 0 40 | 17 0 20 | 24 0 20 | 14 0 20 | 12 0 4 | 23 0  5 | 17 0  5 | 24 0  5 | 28 0 20 | 21 0 10 | 20 0  5 | 33 0 1 | 25 0  5 | 20 0  5 | 21 0  5 | 27 0  5 | 23 0 1 | 24 0  5 | 20 0  5 | 25 0 1 | 20 0  5 | 19 0  5 | 23 0  5 | 19 0  5 | 14 0  5 | 16 0  5 | 17 0  5 | 19 0  5 | 18 0 1 | 23 0  5 | 19 0 1 | 22 0 1 | 19 0  5 | 23 0 1 | 19 0  5 | 21 0  5 | 20 0  5 | 19 0  5 | 20 0 1 | 27 0 1 | 18 0  5 | 20 0 1 | 16 0  5 | 26 0  5 | 27 0  5 | 24 0  5 |
| tvxf4nan | 2 | F | **ADS ChM AMP** | 7 0 60 | 5 0 60 | 1 0 60 | 5 0 60 | 6 0 40 | 19 0 30 | 8 0 40 | 11 0 40 | 10 0 40 | 9 0 40 | 11 0 40 | 4 0 40 | 4 0 40 | 5 0 40 | 3 0 40 | 3 0 40 | 2 0 40 | 5 0 40 | 4 0 40 | 4 0 40 | 24 0 4 | 14 0  5 | 15 0  5 | 14 0 20 | 10 0 40 | 12 0 20 | 7 0 20 | 16 0 20 | 7 0 40 | 7 0 40 | 17 0  5 | 8 0 10 | 3 0 10 | 29 0 10 | 25 0 40 | 20 0 40 | 18 0 10 | 23 0 10 | 28 0 10 | 20 0 40 | 9 0 40 | 22 0 10 | 25 0 40 | 14 0 10 | 14 0 40 | 8 0 40 | 5 0 10 | 8 0 40 | 8 0 40 | 6 0 10 | 8 0 40 | 2 0 40 | 3 0 10 | 2 0  5 | 3 0 20 | 15 0 40 | 8 0 40 | 3 0 40 | 6 0 20 | 5 0 10 |
| ordk4pvw | 2 | F | **ADS ChM AMP** | 11 0 60 | 16 0 30 | 31 0 40 | 15 0 30 | 16 0 30 | 10 0 60 | 14 0 30 | 7 0 40 | 7 0  5 | 20 0 4 | 10 0 1 | 5 0 1 | 8 0 4 | 15 0 4 | 9 0 4 | 23 0 1 | 37 0 1 | 31 0 1 | 35 0 1 | 31 0 1 | 36 0 1 | 31 0 1 | 26 0 1 | 28 0 1 | 26 0 1 | 31 0 1 | 34 0 1 | 35 0 1 | 34 0 1 | 30 0 1 | 30 0 1 | 30 0 1 | 35 0 1 | 33 0 1 | 25 0 1 | 17 0 1 | 18 0 1 | 19 0 1 | 20 0 1 | 16 0 1 | 17 0 1 | 13 0 1 | 14 0 1 | 15 0 1 | 5 0 1 | 6 0 1 | 9 0 1 | 9 0 1 | 10 0 1 | 12 0 1 | 12 0 1 | 10 0 1 | 10 0 1 | 8 0 1 | 8 0 1 | 10 0 1 | 8 0 1 | 9 0 1 | 9 0 1 | 7 0 1 |
