# Supplementary material for: Measuring Mindfulness: A Psychophysiological Approach
Source: Front Hum Neurosci. 2018 Jun 28;12:249. doi: 10.3389/fnhum.2018.00249 (PMC6031749; doi:10.3389/fnhum.2018.00249)
Supplement: Supplementary file 3 [file Data_Sheet_1.ZIP › BostanovEtAlMinfulness_Suppl_data/BostanovEtAlMinfulness_Suppl_qData.html]

qData


# qData

## Legend

|  |  |
| --- | --- |
| SCID, LIFE, etc. |
| NMD | Number of Major Depressive Episodes |
| SCID2 | Selected Personality Disorders |
| QIDS | Quick Inventory of Depressive Symptomatology |
| LIFE | Various Retrospective Scores? |
| etc. | ? |
| Weekly Measures |
| ADS | Allgemeine Depressionsskala |
| AMP | Amount of Mindfulness Practice |
| ChM | Changes in Medication |
| Trait Measures |
| eh | Therapieerwartung und Therapieevaluation (PATHEV): Hoffnung auf Verbesserung (Zuversicht) |
| ef | Therapieerwartung und Therapieevaluation (PATHEV): Furcht vor Veränderung |
| ep | Therapieerwartung und Therapieevaluation (PATHEV & POSTTH): Passung |
| ea | Therapieevaluation (POSTTH): Abwesenheit (number of missed therapy sessions) |
| rsy | Response Styles Questionnaire (RSQ): Symptom-focused Rumination |
| rse | Response Styles Questionnaire (RSQ): Self-focused Rumination |
| rdi | Response Styles Questionnaire (RSQ): Distraction |
| ma | Mindfulness Attention Awareness Scale (MAAS) |
| cr | Co-Rumination Questionnaire (CRQ) |
| State Measures |
| bdi | Beck Depression Inventory |
| p | PANAS: Positive Affect |
| n | PANAS: Negative Affect |
| b | Behavioral Measure: Mouse Click after 1st Tone |
| bm | Behavioral Measure: Mouse Click after 2nd Tone |
| tc | Toronto Mindfulness Scale (TMS): Curiosity Subscore |
| td | Toronto Mindfulness Scale (TMS): Decentering Subscore |
| tm | Toronto Mindfulness Scale (TMS): Total Score |
| Session & Order | xSO, e.g., bdi1, p32, td13, etc. |
| **S**ession Number: |
| S = 1 | Pre-Therapy |
| S = 2 | Post-Therapy |
| S = 3 | Follow-Up |
| **O**rder of Measurement: |
| O = 1 | In the Beginning of the Experiment |
| O = 2 | After the Mood Induction |
| O = 3 | After the Passive ERP/CNV |
| O = 4 | After the Active ERP/CNV |
| Therapy, Group & ID |
| t | Therapy:   CT (t = 1)   or   MBCT (t = 2) |
| g | Group |
| id | Patient ID |

## Scores

| 1 | 2 | 3 | 4 | 5 | 6 | 7 | 8 | 9 | 10 | 11 | 12 | 13 | 14 | 15 | 16 | 17 | 18 | 19 | 20 | 21 | 22 | 23 | 24 | 25 | 26 | 27 | 28 | 29 | 30 | 31 | 32 | 33 | 34 | 35 | 36 | 37 | 38 | 39 | 40 | 41 | 42 | 43 | 44 | 45 | 46 | 47 | 48 | 49 | 50 | 51 | 52 | 53 | 54 | 55 | 56 | 57 | 58 | 59 | 60 | 61 | 62 | 63 | 64 | 65 | 66 | 67 |
| --- | --- | --- | --- | --- | --- | --- | --- | --- | --- | --- | --- | --- | --- | --- | --- | --- | --- | --- | --- | --- | --- | --- | --- | --- | --- | --- | --- | --- | --- | --- | --- | --- | --- | --- | --- | --- | --- | --- | --- | --- | --- | --- | --- | --- | --- | --- | --- | --- | --- | --- | --- | --- | --- | --- | --- | --- | --- | --- | --- | --- | --- | --- | --- | --- | --- | --- |
| id | t | g | sex | age | edu | eh1 | ef1 | ep1 | ep2 | ea2 | rsy1 | rse1 | rdi1 | ma1 | rsy3 | rse3 | rdi3 | ma3 | cr1 | cr2 | cr3 | bdi1 | bdi2 | bdi3 | p11 | n11 | p12 | n12 | p13 | n13 | p21 | n21 | p22 | n22 | p23 | n23 | p31 | n31 | p32 | n32 | p33 | n33 | b1 | bm1 | b2 | bm2 | b3 | bm3 | tc13 | td13 | tm13 | tc14 | td14 | tm14 | tc23 | td23 | tm23 | tc24 | td24 | tm24 | tc33 | td33 | tm33 | tc34 | td34 | tm34 |
| ukgr6ibi | 1 | A | f | 56 | GY | 16 | 3 | 13 | 25 | 1 | 11 | 14 | 13 | 45 | 11 | 12 | 15 | 42 | ? | ? | 35 | 10 | 17 | 17 | 30 | 14 | 19 | 13 | 26 | 12 | 27 | 16 | 17 | 11 | 25 | 10 | 23 | 12 | 15 | 10 | 17 | 10 | 50 | 39 | 50 | 37 | 47 | 37 | 12 | 17 | 29 | 17 | 17 | 34 | 17 | 17 | 34 | 14 | 18 | 32 | 5 | 20 | 25 | 9 | 17 | 26 |
| lunu6vmb | 1 | A | f | 45 | UN | 14 | 3 | 16 | 37 | 0 | 25 | 14 | 11 | 51 | 20 | 11 | 19 | 58 | ? | ? | 77 | 5 | 2 | 13 | 30 | 13 | 13 | 16 | 29 | 10 | 23 | 11 | 13 | 14 | 13 | 11 | 18 | 12 | 11 | 11 | 17 | 10 | 48 | 38 | 49 | 48 | 50 | 36 | 9 | 19 | 28 | 9 | 15 | 24 | 8 | 11 | 19 | 6 | 22 | 28 | 5 | 10 | 15 | 7 | 11 | 18 |
| lrmj6jew | 1 | A | f | 58 | GY | 14 | 5 | 10 | 36 | 0 | 19 | 17 | 21 | 46 | 16 | 14 | 18 | 49 | ? | ? | 67 | 16 | 18 | 15 | 20 | 20 | 10 | 20 | 14 | 22 | 16 | 18 | 14 | 22 | 17 | 21 | 17 | 17 | 16 | 13 | 18 | 14 | 47 | 47 | 47 | 16 | 50 | 50 | 11 | 9 | 20 | 12 | 11 | 23 | 9 | 12 | 21 | 12 | 8 | 20 | 7 | 8 | 15 | 4 | 3 | 7 |
| gpod8wgm | 1 | A | m | 51 | UN | 18 | 3 | 11 | 26 | 1 | 25 | 18 | 18 | 66 | 15 | 9 | 14 | 58 | ? | ? | 55 | 13 | 22 | 9 | 23 | 11 | 22 | 11 | 25 | 10 | 20 | 15 | 16 | 16 | 17 | 11 | 33 | 12 | 16 | 11 | 16 | 10 | 48 | 32 | 49 | 25 | 44 | 33 | 22 | 19 | 41 | 16 | 18 | 34 | 12 | 15 | 27 | 8 | 12 | 20 | 5 | 15 | 20 | 6 | 13 | 19 |
| ronr6tsn | 1 | A | f | 43 | UN | 14 | 4 | 8 | 40 | 0 | 21 | 23 | 20 | 48 | 18 | 18 | 18 | 52 | ? | ? | 118 | 1 | 3 | 10 | 28 | 12 | 17 | 22 | 26 | 10 | 20 | 11 | 16 | 12 | 15 | 11 | 18 | 11 | 13 | 14 | 16 | 11 | 49 | 28 | 37 | 37 | 25 | 27 | 13 | 20 | 33 | 11 | 18 | 29 | 13 | 10 | 23 | 15 | 12 | 27 | 7 | 9 | 16 | 6 | 6 | 12 |
| iuhu6gyr | 1 | A | f | 61 | UN | 19 | 3 | 18 | 38 | 1 | 15 | 15 | 10 | 85 | 16 | 8 | 17 | 78 | ? | ? | 32 | 1 | 10 | 0 | 38 | 10 | 24 | 10 | 22 | 10 | 22 | 11 | 16 | 12 | 12 | 10 | 45 | 10 | 45 | 10 | 27 | 10 | 48 | 41 | 50 | 38 | 49 | 28 | 10 | 13 | 23 | 0 | 20 | 20 | 16 | 14 | 30 | 13 | 15 | 28 | 21 | 22 | 43 | 16 | 19 | 35 |
| irjh8pxw | 1 | A | m | 56 | UN | 19 | 3 | 16 | 43 | 0 | 18 | 15 | 20 | 60 | 9 | 9 | 26 | 65 | ? | ? | 66 | 0 | 0 | ? | 36 | 13 | 15 | 19 | 22 | 13 | 36 | 11 | 25 | 11 | 26 | 10 | ? | ? | ? | ? | ? | ? | 45 | 43 | 40 | 47 | ? | ? | 13 | 16 | 29 | 11 | 17 | 28 | 16 | 13 | 29 | 16 | 18 | 34 | ? | ? | ? | ? | ? | ? |
| npod6ehm | 1 | A | f | 41 | GY | 17 | 5 | 16 | 41 | 0 | 24 | 20 | 18 | 48 | 14 | 11 | 24 | 66 | ? | ? | 62 | 11 | 1 | 4 | 33 | 15 | 18 | 17 | 25 | 10 | 35 | 13 | 17 | 22 | 34 | 11 | 35 | 12 | 22 | 18 | 33 | 10 | 44 | 26 | 49 | 34 | 49 | 28 | 19 | 17 | 36 | 9 | 18 | 27 | 16 | 16 | 32 | 12 | 10 | 22 | 12 | 17 | 29 | 9 | 9 | 18 |
| uger6lhp | 1 | C | f | 62 | GY | ? | ? | ? | 34 | 0 | 12 | 11 | 18 | 79 | ? | ? | ? | ? | ? | ? | ? | 11 | 11 | 5 | 30 | 12 | 24 | 19 | 22 | 14 | 30 | 14 | 24 | 16 | 23 | 11 | 29 | 13 | 18 | 17 | 24 | 12 | 43 | 33 | 41 | 41 | 15 | 46 | 8 | 16 | 24 | 8 | 15 | 23 | 11 | 11 | 22 | 11 | 11 | 22 | 6 | 10 | 16 | 4 | 9 | 13 |
| lkat5awd | 1 | C | f | 29 | UN | 17 | 3 | 17 | 41 | 0 | 21 | 23 | 14 | 66 | 10 | 16 | 14 | 62 | 69 | 50 | 52 | 2 | 7 | 4 | 29 | 14 | 12 | 13 | 11 | 10 | 12 | 12 | 10 | 10 | 10 | 10 | 16 | 10 | 10 | 10 | 10 | 10 | 50 | 20 | 49 | 19 | 49 | 16 | 3 | 15 | 18 | 2 | 3 | 5 | 0 | 3 | 3 | 0 | 3 | 3 | 0 | 11 | 11 | 0 | 7 | 7 |
| fdvd4bzu | 1 | C | f | 55 | MR | 16 | 5 | 17 | 40 | 0 | 23 | 18 | 15 | 52 | 12 | 12 | 23 | 74 | 66 | 69 | 49 | 17 | 6 | 0 | 27 | 15 | 20 | 19 | 21 | 15 | 33 | 14 | 17 | 18 | 20 | 16 | 40 | 10 | 20 | 12 | 25 | 10 | 50 | 11 | 50 | 28 | 50 | 44 | 8 | 8 | 16 | 7 | 8 | 15 | 2 | 3 | 5 | 3 | 6 | 9 | 3 | 8 | 11 | 6 | 6 | 12 |
| rhua5phi | 1 | C | f | 31 | GY | 12 | 3 | 13 | 39 | 0 | 21 | 19 | 11 | 64 | 18 | 10 | 19 | 71 | 69 | 68 | 59 | 16 | 12 | 1 | 18 | 11 | 17 | 12 | 18 | 10 | 20 | 10 | 14 | 11 | 19 | 10 | 25 | 11 | 17 | 12 | 25 | 10 | 48 | 43 | 50 | 50 | 50 | 50 | 12 | 15 | 27 | 7 | 15 | 22 | 6 | 18 | 24 | 8 | 18 | 26 | 9 | 15 | 24 | 8 | 22 | 30 |
| kuyk4bcl | 1 | C | f | 54 | MR | 12 | 5 | 13 | 36 | 1 | 21 | 15 | 17 | 46 | 20 | 16 | 17 | 45 | 96 | 51 | 81 | 35 | 5 | 21 | 24 | 19 | 20 | 17 | 18 | 16 | 28 | 12 | 12 | 24 | 28 | 12 | 18 | 11 | 13 | 14 | 12 | 13 | 49 | 24 | 49 | 28 | 50 | 21 | 6 | 12 | 18 | 6 | 11 | 17 | 16 | 15 | 31 | 9 | 14 | 23 | 0 | 13 | 13 | 1 | 7 | 8 |
| xvip2ttr | 1 | C | m | 44 | UN | 16 | 6 | 15 | 35 | 0 | 25 | 17 | 13 | 67 | 19 | 14 | 11 | 65 | 67 | 51 | 56 | 0 | 1 | ? | 25 | 11 | 16 | 16 | 21 | 11 | 31 | 11 | 17 | 18 | 26 | 11 | ? | ? | ? | ? | ? | ? | 22 | 29 | 49 | 38 | ? | ? | 3 | 10 | 13 | 2 | 11 | 13 | 7 | 9 | 16 | 2 | 9 | 11 | ? | ? | ? | ? | ? | ? |
| orer5lwy | 1 | C | f | 38 | UN | 15 | 3 | 13 | 43 | 0 | 22 | 12 | 12 | 53 | 11 | 8 | 21 | 82 | 66 | 57 | 44 | 5 | 1 | 6 | 29 | 11 | 16 | 12 | 29 | 10 | 30 | 10 | 30 | 12 | 37 | 10 | 39 | 14 | 18 | 11 | 30 | 10 | 49 | 29 | 43 | 31 | 47 | 24 | 13 | 14 | 27 | 16 | 19 | 35 | 17 | 20 | 37 | 17 | 15 | 32 | 18 | 20 | 38 | 17 | 17 | 34 |
| edbu8yrd | 1 | C | m | 57 | UN | 13 | 6 | 12 | 38 | 0 | 17 | 12 | 13 | 54 | 16 | 12 | 14 | 50 | 62 | 61 | 86 | 15 | 13 | 18 | 24 | 18 | 17 | 13 | 33 | 12 | 26 | 12 | 19 | 11 | 20 | 11 | 22 | 15 | 25 | 11 | 22 | 10 | 50 | 32 | 49 | 42 | 49 | 32 | 9 | 12 | 21 | 12 | 15 | 27 | 7 | 13 | 20 | 10 | 18 | 28 | 7 | 13 | 20 | 7 | 16 | 23 |
| uwxu5xig | 1 | G | f | 22 | GY | 19 | 3 | 14 | 18 | 3 | 23 | 10 | 14 | 71 | ? | ? | ? | ? | 84 | 85 | ? | 1 | 2 | ? | 29 | 11 | 24 | 12 | 16 | 11 | 30 | 12 | 20 | 10 | 29 | 10 | ? | ? | ? | ? | ? | ? | 50 | 45 | 49 | 47 | ? | ? | 10 | 7 | 17 | 6 | 10 | 16 | 13 | 15 | 28 | 9 | 15 | 24 | ? | ? | ? | ? | ? | ? |
| auxp2tem | 1 | G | m | 45 | UN | 16 | 3 | 13 | 40 | 0 | 16 | 16 | 16 | 63 | 16 | 15 | 19 | 49 | 59 | 51 | 68 | 6 | 1 | ? | 31 | 10 | 14 | 12 | 13 | 10 | 37 | 10 | 21 | 11 | 20 | 13 | ? | ? | ? | ? | ? | ? | 49 | 50 | 46 | 46 | ? | ? | 8 | 14 | 22 | 9 | 21 | 30 | 3 | 15 | 18 | 2 | 14 | 16 | ? | ? | ? | ? | ? | ? |
| hpod5kla | 1 | G | f | 23 | UN | 16 | 5 | 13 | 28 | 1 | 27 | 22 | 14 | 59 | 15 | 18 | 17 | 69 | 65 | 53 | 67 | 4 | 8 | ? | 29 | 12 | 17 | 20 | 13 | 13 | 27 | 10 | 17 | 15 | 19 | 14 | ? | ? | ? | ? | ? | ? | 48 | 26 | 49 | 36 | ? | ? | 10 | 19 | 29 | 8 | 2 | 10 | 16 | 18 | 34 | 11 | 24 | 35 | ? | ? | ? | ? | ? | ? |
| nuxf5dmp | 1 | G | f | 26 | UN | 15 | 8 | 14 | 37 | 0 | 18 | 23 | 13 | 47 | ? | ? | ? | ? | 81 | 77 | ? | 5 | 4 | ? | 33 | 12 | 18 | 21 | 33 | 10 | 33 | 11 | 16 | 31 | 34 | 12 | ? | ? | ? | ? | ? | ? | 50 | 36 | 49 | 36 | ? | ? | 10 | 17 | 27 | 11 | 18 | 29 | 10 | 17 | 27 | 14 | 15 | 29 | ? | ? | ? | ? | ? | ? |
| nsmu5nph | 1 | G | f | 28 | UN | 8 | 3 | 8 | 35 | 4 | 28 | 21 | 16 | 65 | 19 | 12 | 20 | 78 | 109 | 78 | 64 | 11 | 14 | ? | 33 | 12 | 15 | 14 | 16 | 12 | 38 | 10 | 10 | 14 | 11 | 17 | ? | ? | ? | ? | ? | ? | 48 | 33 | 50 | 38 | ? | ? | 5 | 16 | 21 | 17 | 16 | 33 | 6 | 6 | 12 | 0 | 4 | 4 | ? | ? | ? | ? | ? | ? |
| wuxv5xog | 1 | G | f | 22 | UN | 15 | 7 | 14 | 26 | 4 | 19 | 22 | 16 | 56 | ? | ? | ? | ? | 97 | 99 | ? | 11 | 16 | ? | 30 | 11 | 15 | 11 | 11 | 13 | 20 | 10 | 13 | 10 | 15 | 11 | ? | ? | ? | ? | ? | ? | 48 | 16 | 30 | 8 | ? | ? | 12 | 5 | 17 | 7 | 3 | 10 | 7 | 10 | 17 | 6 | 12 | 18 | ? | ? | ? | ? | ? | ? |
| vper5lkp | 1 | G | f | 39 | MR | 16 | 3 | 13 | 42 | 4 | 16 | 12 | 19 | 54 | ? | ? | ? | ? | 61 | 58 | ? | 3 | 0 | ? | 27 | 10 | 11 | 13 | 22 | 10 | 29 | 10 | 18 | 17 | ? | ? | ? | ? | ? | ? | ? | ? | 43 | 44 | 45 | 48 | ? | ? | 14 | 17 | 31 | 18 | 20 | 38 | ? | ? | ? | 18 | 19 | 37 | ? | ? | ? | ? | ? | ? |
| orit5aym | 1 | G | f | 30 | UN | 13 | 4 | 14 | 25 | 4 | 27 | 17 | 15 | 51 | ? | ? | ? | ? | 58 | ? | ? | 4 | 3 | ? | 26 | 15 | 20 | 17 | 17 | 10 | 21 | 10 | 14 | 12 | 13 | 11 | ? | ? | ? | ? | ? | ? | 48 | 27 | 45 | 37 | ? | ? | 0 | 9 | 9 | 7 | 11 | 18 | 0 | 16 | 16 | 5 | 19 | 24 | ? | ? | ? | ? | ? | ? |
| uwiu8bon | 2 | B | m | 29 | UN | 16 | 4 | 16 | 33 | 4 | 21 | 22 | 13 | 60 | 17 | 14 | 18 | 57 | ? | ? | 88 | 9 | 9 | 2 | 28 | 10 | 19 | 13 | 21 | 12 | 28 | 15 | 20 | 14 | 22 | 12 | 21 | 11 | 16 | 14 | 15 | 12 | 50 | 32 | 49 | 30 | 48 | 26 | 8 | 8 | 16 | 8 | 10 | 18 | 10 | 17 | 27 | 13 | 17 | 30 | 8 | 14 | 22 | 10 | 14 | 24 |
| uwxf6wfj | 2 | B | f | 51 | MR | 19 | 5 | 19 | 45 | 0 | 14 | 12 | 10 | 66 | 8 | 8 | 12 | 79 | ? | ? | 68 | 0 | 0 | 0 | 43 | 12 | 35 | 10 | 20 | 15 | 47 | 10 | 37 | 10 | 50 | 10 | 37 | 10 | 40 | 10 | 44 | 10 | 48 | 41 | 50 | 49 | 48 | 50 | 9 | 14 | 23 | 11 | 13 | 24 | 13 | 17 | 30 | 12 | 15 | 27 | 12 | 14 | 26 | 12 | 14 | 26 |
| lkkd6wwp | 2 | B | f | 51 | MR | 20 | 4 | 20 | 42 | 3 | 19 | 11 | 13 | 74 | 15 | 11 | 20 | 71 | ? | ? | 35 | 7 | 2 | 8 | 39 | 11 | 46 | 10 | 41 | 10 | 36 | 12 | 24 | 10 | 19 | 10 | 28 | 13 | 22 | 10 | 18 | 10 | 46 | 49 | 36 | 23 | 32 | 44 | 6 | 9 | 15 | 0 | 0 | 0 | 1 | 0 | 1 | 1 | 4 | 5 | 11 | 12 | 23 | 0 | 3 | 3 |
| fdlp6ihd | 2 | B | f | 31 | UN | 17 | 4 | 18 | 40 | 4 | 21 | 16 | 16 | 42 | 16 | 18 | 11 | 60 | ? | ? | 71 | 4 | 4 | 7 | 22 | 19 | 14 | 16 | 22 | 29 | 18 | 16 | 10 | 18 | 15 | 12 | 30 | 18 | 14 | 12 | 17 | 10 | 50 | 16 | 48 | 26 | 50 | 25 | 6 | 6 | 12 | 0 | 4 | 4 | 8 | 11 | 19 | 10 | 12 | 22 | 16 | 17 | 33 | 8 | 14 | 22 |
| jkld8jrm | 2 | B | m | 59 | UN | 17 | 6 | 16 | 33 | 3 | 20 | 14 | 14 | 67 | 19 | 13 | 16 | 61 | ? | ? | 73 | 13 | 22 | 15 | 33 | 13 | 35 | 11 | 39 | 12 | 23 | 26 | 24 | 20 | 29 | 14 | 27 | 13 | 24 | 13 | 28 | 12 | 50 | 48 | 50 | 42 | 50 | 46 | 21 | 20 | 41 | 21 | 20 | 41 | 13 | 13 | 26 | 15 | 15 | 30 | 17 | 16 | 33 | 17 | 16 | 33 |
| ytxv6ipg | 2 | B | f | 56 | UN | 17 | 4 | 18 | 42 | 2 | 17 | 8 | 11 | 51 | 14 | 8 | 23 | 79 | ? | ? | 71 | 7 | 0 | 0 | 28 | 16 | 21 | 16 | 28 | 12 | 34 | 10 | 23 | 11 | 35 | 10 | 36 | 10 | 22 | 15 | 30 | 10 | 33 | 47 | 32 | 35 | 26 | 43 | 13 | 13 | 26 | 15 | 12 | 27 | 14 | 17 | 31 | 16 | 19 | 35 | 15 | 19 | 34 | 16 | 22 | 38 |
| iuau8mnw | 2 | B | m | 48 | UN | 18 | 3 | 16 | 45 | 0 | 16 | 15 | 19 | 57 | 16 | 12 | 17 | 62 | ? | ? | 51 | 3 | 6 | 3 | 33 | 12 | 21 | 17 | 31 | 10 | 28 | 10 | 18 | 13 | 22 | 11 | 24 | 11 | 14 | 13 | 19 | 10 | 49 | 33 | 50 | 47 | 50 | 43 | 16 | 17 | 33 | 24 | 27 | 51 | 5 | 13 | 18 | 24 | 28 | 52 | 1 | 16 | 17 | 18 | 24 | 42 |
| auxv6azb | 2 | B | f | 53 | UN | 16 | 3 | 17 | 41 | 0 | 19 | 12 | 16 | 62 | 14 | 11 | 18 | 71 | ? | ? | 53 | 3 | 3 | ? | 25 | 10 | 20 | 11 | 14 | 11 | 19 | 10 | 15 | 11 | 27 | 10 | ? | ? | ? | ? | ? | ? | 3 | 28 | 34 | 36 | ? | ? | 5 | 8 | 13 | 3 | 6 | 9 | 12 | 21 | 33 | 5 | 15 | 20 | ? | ? | ? | ? | ? | ? |
| nuvk8aeg | 2 | B | m | 28 | UN | 14 | 5 | 15 | 36 | 1 | 25 | 18 | 19 | 53 | 21 | 17 | 18 | 50 | ? | ? | 86 | 12 | 10 | 11 | 30 | 15 | 21 | 18 | 31 | 17 | 28 | 17 | 24 | 21 | 22 | 18 | 30 | 26 | 26 | 25 | 28 | 22 | 50 | 26 | 48 | 35 | 48 | 23 | 14 | 11 | 25 | 17 | 9 | 26 | 10 | 12 | 22 | 17 | 10 | 27 | 13 | 14 | 27 | 11 | 10 | 21 |
| kdxf8rrl | 2 | B | m | 59 | UN | 18 | 5 | 17 | 44 | 0 | 18 | 13 | 17 | 61 | 19 | 13 | 18 | 68 | ? | ? | 62 | 8 | 12 | 18 | 31 | 11 | 16 | 13 | 29 | 11 | 27 | 12 | 18 | 20 | 29 | 10 | 28 | 11 | 20 | 16 | 30 | 10 | 50 | 32 | 49 | 40 | 50 | 39 | 8 | 7 | 15 | 4 | 7 | 11 | 12 | 19 | 31 | 13 | 20 | 33 | 12 | 20 | 32 | 9 | 18 | 27 |
| xpad6okp | 2 | B | f | 43 | UN | 15 | 4 | 16 | 44 | 0 | 19 | 19 | 16 | 48 | 25 | 19 | 18 | 56 | ? | ? | 68 | 4 | 1 | 1 | 30 | 15 | 17 | 15 | 21 | 11 | 27 | 13 | 16 | 14 | 24 | 10 | 18 | 11 | 13 | 13 | 18 | 12 | 49 | 38 | 48 | 44 | 49 | 49 | 14 | 17 | 31 | 18 | 20 | 38 | 12 | 17 | 29 | 14 | 12 | 26 | 6 | 10 | 16 | 7 | 13 | 20 |
| tkyd6dsg | 2 | B | f | 49 | GY | 15 | 3 | 14 | 45 | 2 | 24 | 20 | 16 | 26 | 17 | 15 | 21 | 57 | ? | ? | 86 | 12 | 4 | 4 | 22 | 29 | 20 | 27 | 16 | 16 | 28 | 11 | 14 | 10 | 22 | 10 | 27 | 14 | 20 | 12 | 14 | 10 | 50 | 38 | 48 | 40 | 47 | 30 | 7 | 11 | 18 | 7 | 10 | 17 | 18 | 19 | 37 | 16 | 22 | 38 | 8 | 17 | 25 | 14 | 15 | 29 |
| uhjd2sed | 2 | D | m | 39 | UN | 15 | 4 | 14 | 43 | 0 | 18 | 12 | 11 | 63 | 10 | 10 | 13 | 78 | 41 | 33 | 31 | 5 | 5 | 3 | 33 | 10 | 16 | 15 | 18 | 10 | 27 | 10 | 13 | 11 | 33 | 10 | 30 | 10 | 18 | 13 | 21 | 10 | 49 | 34 | 49 | 38 | 42 | 31 | 8 | 7 | 15 | 7 | 11 | 18 | 3 | 10 | 13 | 9 | 14 | 23 | 8 | 11 | 19 | 5 | 13 | 18 |
| uhat8lkg | 2 | D | m | 38 | UN | 20 | 3 | 17 | 42 | 2 | 29 | 13 | 13 | 66 | 27 | 17 | 14 | 64 | 76 | 87 | 104 | 1 | 11 | 13 | 23 | 11 | 11 | 23 | 23 | 12 | 17 | 13 | 11 | 19 | 22 | 11 | 15 | 11 | 12 | 12 | 14 | 13 | 50 | 32 | 48 | 28 | 50 | 37 | 3 | 13 | 16 | 6 | 15 | 21 | 17 | 21 | 38 | 15 | 22 | 37 | 0 | 18 | 18 | 12 | 22 | 34 |
| lpxf4bzn | 2 | D | f | 55 | MR | 16 | 4 | 15 | 44 | 1 | 19 | 16 | 19 | 60 | 13 | 12 | 21 | 66 | 53 | 42 | 60 | 0 | 0 | 7 | 42 | 12 | 21 | 15 | 26 | 11 | 32 | 12 | 21 | 10 | 28 | 10 | 30 | 14 | 20 | 13 | 24 | 10 | 49 | 34 | 50 | 36 | 49 | 41 | 14 | 14 | 28 | 4 | 10 | 14 | 15 | 16 | 31 | 16 | 15 | 31 | 15 | 18 | 33 | 14 | 18 | 32 |
| fiah4ndi | 2 | D | f | 52 | MR | 18 | 3 | 15 | 45 | 0 | 13 | 12 | 12 | 80 | 15 | 12 | 21 | 76 | 69 | 52 | 62 | 0 | 19 | 0 | 41 | 11 | 16 | 29 | 27 | 11 | 38 | 15 | 15 | 32 | 35 | 10 | 44 | 14 | 11 | 39 | 39 | 10 | 50 | 42 | 50 | 40 | 50 | 40 | 15 | 13 | 28 | 17 | 17 | 34 | 22 | 24 | 46 | 23 | 24 | 47 | 21 | 26 | 47 | 18 | 22 | 40 |
| road6wfm | 2 | D | f | 26 | UN | 18 | 3 | 17 | 41 | 0 | 23 | 22 | 15 | 54 | 10 | 8 | 22 | 64 | 101 | 80 | 48 | 5 | 2 | 1 | 32 | 13 | 20 | 16 | 33 | 10 | 42 | 15 | 24 | 16 | 29 | 11 | 44 | 13 | 31 | 10 | 26 | 10 | 49 | 31 | 49 | 26 | 50 | 25 | 20 | 18 | 38 | 11 | 12 | 23 | 14 | 17 | 31 | 9 | 17 | 26 | 12 | 14 | 26 | 11 | 17 | 28 |
| phfk6wdj | 2 | D | f | 51 | UN | 17 | 5 | 13 | 33 | 0 | 21 | 21 | 14 | 62 | 17 | 20 | 16 | 63 | 78 | 60 | 66 | 7 | 11 | 11 | 26 | 10 | 17 | 12 | 18 | 11 | 22 | 11 | 15 | 13 | 14 | 13 | 22 | 13 | 12 | 22 | 17 | 20 | 50 | 22 | 47 | 21 | 48 | 23 | 11 | 11 | 22 | 12 | 14 | 26 | 10 | 15 | 25 | 0 | 0 | 0 | 9 | 10 | 19 | 11 | 10 | 21 |
| phnd5fdf | 2 | D | f | 37 | GY | 19 | 4 | 20 | 41 | 0 | 22 | 18 | 9 | 79 | 12 | 11 | 22 | 81 | 54 | 50 | 35 | 0 | 0 | 0 | 40 | 11 | 28 | 16 | 25 | 10 | 38 | 10 | 23 | 11 | 29 | 10 | 39 | 11 | 25 | 14 | 28 | 10 | 50 | 19 | 48 | 40 | 50 | 28 | 13 | 15 | 28 | 10 | 12 | 22 | 11 | 18 | 29 | 14 | 19 | 33 | 13 | 16 | 29 | 16 | 17 | 33 |
| brrw5xda | 2 | D | f | 21 | UN | 18 | 3 | 20 | 28 | 0 | 23 | 16 | 13 | 45 | 18 | 15 | 22 | 72 | 58 | 42 | 44 | 0 | 1 | 1 | 36 | 11 | 23 | 20 | 25 | 12 | 37 | 11 | 22 | 19 | 20 | 13 | 39 | 13 | 34 | 19 | 30 | 13 | 50 | 43 | 49 | 36 | 50 | 35 | 16 | 16 | 32 | 17 | 20 | 37 | 15 | 17 | 32 | 17 | 16 | 33 | 13 | 14 | 27 | 11 | 14 | 25 |
| ndxf6dvi | 2 | D | f | 50 | UN | 13 | 6 | 13 | 35 | 4 | 20 | 17 | 11 | 60 | 27 | 19 | 13 | 43 | 45 | 54 | 50 | 18 | 28 | 9 | 20 | 11 | 13 | 28 | 20 | 13 | 18 | 16 | 18 | 26 | 23 | 13 | 27 | 11 | 15 | 23 | 32 | 12 | 43 | 26 | 50 | 36 | 49 | 33 | 7 | 11 | 18 | 7 | 7 | 14 | 13 | 14 | 27 | 18 | 15 | 33 | 19 | 15 | 34 | 17 | 11 | 28 |
| kdir4wwb | 2 | D | f | 51 | GY | 16 | 6 | 10 | 40 | 3 | 24 | 15 | 12 | 54 | 24 | 11 | 16 | 59 | 75 | 41 | 65 | 3 | 20 | 20 | 21 | 12 | 14 | 23 | 12 | 15 | 18 | 14 | 14 | 12 | 15 | 12 | 20 | 11 | 14 | 18 | 14 | 10 | 50 | 47 | 31 | 45 | 39 | 39 | 3 | 7 | 10 | 4 | 8 | 12 | 6 | 14 | 20 | 4 | 6 | 10 | 5 | 14 | 19 | 3 | 11 | 14 |
| ktlk4hll | 2 | D | f | 54 | UN | 15 | 5 | 13 | 36 | 0 | 14 | 19 | 17 | 45 | 13 | 17 | 19 | 44 | 69 | 76 | 66 | 5 | 3 | 19 | 26 | 22 | 11 | 20 | 26 | 10 | 28 | 14 | 10 | 20 | 22 | 18 | 23 | 19 | 10 | 20 | 10 | 10 | 50 | 30 | 50 | 38 | 50 | 41 | 6 | 7 | 13 | 6 | 7 | 13 | 6 | 12 | 18 | 6 | 8 | 14 | 7 | 14 | 21 | 7 | 12 | 19 |
| xulu4idp | 2 | D | f | 57 | MR | 13 | 5 | 15 | 38 | 0 | 15 | 11 | 21 | 63 | 15 | 11 | 18 | 64 | 56 | 45 | 31 | 1 | 8 | 4 | 25 | 12 | 19 | 22 | 20 | 10 | 21 | 16 | 19 | 13 | 21 | 12 | 24 | 15 | 16 | 21 | 12 | 15 | 48 | 17 | 50 | 34 | 50 | 9 | 2 | 8 | 10 | 4 | 7 | 11 | 4 | 7 | 11 | 0 | 7 | 7 | 6 | 6 | 12 | 3 | 4 | 7 |
| ndjp3tmw | 2 | E | m | 20 | MR | 14 | 6 | 14 | ? | ? | 17 | 22 | 20 | 57 | ? | ? | ? | ? | 64 | 80 | ? | 15 | ? | ? | 24 | 21 | 18 | 20 | 18 | 18 | ? | ? | ? | ? | ? | ? | ? | ? | ? | ? | ? | ? | 49 | 20 | ? | ? | ? | ? | 5 | 8 | 13 | 3 | 6 | 9 | ? | ? | ? | ? | ? | ? | ? | ? | ? | ? | ? | ? |
| uwat4xjj | 2 | E | f | 46 | MR | 19 | 4 | 17 | 44 | 0 | 24 | 14 | 17 | 66 | 16 | 8 | 24 | 63 | 54 | ? | 60 | 0 | 0 | 0 | 32 | 10 | 13 | 13 | 17 | 17 | 37 | 11 | 12 | 16 | 17 | 13 | 45 | 12 | 21 | 16 | 25 | 12 | 42 | 19 | 46 | 33 | 46 | 28 | 19 | 20 | 39 | 18 | 22 | 40 | 14 | 17 | 31 | 15 | 20 | 35 | 16 | 17 | 33 | 14 | 16 | 30 |
| lrsd5ztl | 2 | E | f | 23 | UN | 15 | 3 | 12 | 33 | 2 | 32 | 25 | 12 | 44 | 20 | 17 | 14 | 49 | 63 | 75 | 46 | 1 | 10 | 5 | 33 | 12 | 18 | 21 | 22 | 11 | 21 | 15 | 13 | 17 | 17 | 12 | 35 | 16 | 20 | 19 | 25 | 20 | 48 | 45 | 50 | 40 | 48 | 39 | 6 | 15 | 21 | 6 | 19 | 25 | 8 | 15 | 23 | 8 | 16 | 24 | 11 | 17 | 28 | 13 | 18 | 31 |
| lrdx5iiy | 2 | E | f | 32 | UN | 13 | 6 | 14 | 34 | 0 | 16 | 17 | 18 | 70 | ? | ? | ? | ? | 75 | 84 | ? | 3 | 13 | ? | 27 | 15 | 20 | 17 | 21 | 16 | 29 | 19 | 21 | 20 | 27 | 14 | ? | ? | ? | ? | ? | ? | 50 | 17 | 50 | 33 | ? | ? | 11 | 11 | 22 | 10 | 12 | 22 | 14 | 17 | 31 | 17 | 16 | 33 | ? | ? | ? | ? | ? | ? |
| lklu4jal | 2 | E | f | 59 | UN | 16 | 7 | 15 | 32 | 1 | 27 | 19 | 18 | 62 | 19 | 14 | 22 | 56 | 94 | 63 | 72 | 5 | 7 | 15 | 38 | 12 | 28 | 11 | 36 | 13 | 35 | 14 | 25 | 14 | 34 | 11 | 32 | 20 | 19 | 16 | 36 | 12 | 50 | 17 | 50 | 38 | 49 | 44 | 14 | 16 | 30 | 14 | 14 | 28 | 18 | 19 | 37 | 13 | 18 | 31 | 13 | 19 | 32 | 16 | 20 | 36 |
| road4jyg | 2 | E | f | 59 | UN | 17 | 6 | 14 | 40 | 2 | 23 | 15 | 12 | 62 | 19 | 12 | 14 | 61 | 73 | 61 | 66 | 1 | 0 | 10 | 27 | 11 | 14 | 16 | 21 | 10 | 33 | 11 | 16 | 34 | 17 | 16 | 24 | 13 | 12 | 17 | 14 | 12 | 50 | 34 | 50 | 36 | 50 | 34 | 10 | 15 | 25 | 14 | 19 | 33 | 9 | 12 | 21 | 12 | 15 | 27 | 6 | 10 | 16 | 9 | 13 | 22 |
| jufi5igb | 2 | E | f | 33 | UN | 15 | 3 | 14 | 42 | 0 | 25 | 23 | 15 | 57 | 24 | 20 | 16 | 38 | 66 | 57 | 71 | 4 | 12 | 14 | 29 | 18 | 18 | 37 | 34 | 14 | 20 | 24 | 16 | 24 | 24 | 14 | 21 | 15 | 13 | 22 | 22 | 12 | 50 | 39 | 50 | 36 | 50 | 33 | 20 | 15 | 35 | 11 | 14 | 25 | 12 | 23 | 35 | 9 | 19 | 28 | 12 | 17 | 29 | 11 | 20 | 31 |
| irhd4uip | 2 | E | f | 64 | UN | 14 | 4 | 15 | 43 | 1 | 10 | 14 | 16 | 45 | 12 | 18 | 15 | 35 | 72 | 66 | 78 | 10 | 7 | 5 | 31 | 24 | 17 | 12 | 14 | 36 | 29 | 15 | 19 | 20 | 34 | 13 | 21 | 12 | 13 | 12 | 15 | 12 | 45 | 28 | 46 | 32 | 15 | 39 | 14 | 17 | 31 | 15 | 13 | 28 | 13 | 15 | 28 | 16 | 11 | 27 | 6 | 10 | 16 | 7 | 10 | 17 |
| bruk4khl | 2 | E | f | 47 | UN | 18 | 3 | 15 | 43 | 1 | 21 | 19 | 20 | 47 | 13 | 18 | 20 | 67 | 74 | 65 | 48 | 7 | 2 | 3 | 40 | 15 | 17 | 40 | 39 | 15 | 46 | 16 | 13 | 41 | 43 | 13 | 41 | 18 | 14 | 31 | 36 | 16 | 38 | 28 | 49 | 35 | 48 | 33 | 17 | 14 | 31 | 15 | 20 | 35 | 15 | 20 | 35 | 22 | 18 | 40 | 17 | 21 | 38 | 20 | 18 | 38 |
| nupo2syr | 2 | E | m | 40 | UN | 15 | 4 | 13 | 38 | 0 | 23 | 19 | 10 | 53 | 24 | 17 | 11 | 47 | 76 | 69 | 72 | 13 | 12 | 10 | 22 | 23 | 15 | 17 | 14 | 13 | 20 | 12 | 11 | 17 | 13 | 10 | 24 | 11 | 15 | 16 | 20 | 14 | 49 | 37 | 48 | 31 | 47 | 35 | 5 | 7 | 12 | 7 | 10 | 17 | 0 | 8 | 8 | 0 | 11 | 11 | 1 | 12 | 13 | 0 | 12 | 12 |
| kuat2czi | 2 | E | m | 41 | UN | 8 | 7 | 13 | 28 | 1 | 29 | 22 | 12 | 56 | 27 | 22 | 11 | 43 | 60 | 84 | 61 | 7 | 38 | 16 | 31 | 13 | 19 | 31 | 20 | 14 | 17 | 30 | 10 | 34 | 11 | 26 | 28 | 17 | 17 | 20 | 17 | 11 | 50 | 46 | 50 | 41 | 49 | 45 | 3 | 16 | 19 | 13 | 8 | 21 | 5 | 9 | 14 | 4 | 11 | 15 | 5 | 16 | 21 | 9 | 4 | 13 |
| gpfk5bgr | 2 | F | f | 31 | GY | 17 | 3 | 12 | 41 | 0 | 16 | 17 | 17 | 81 | 14 | 18 | 22 | 75 | 66 | 60 | 47 | 0 | 2 | 4 | 36 | 15 | 14 | 31 | 14 | 13 | 40 | 12 | 39 | 13 | 30 | 10 | 38 | 13 | 18 | 13 | 24 | 10 | 50 | 28 | 48 | 33 | 49 | 16 | 3 | 4 | 7 | 22 | 10 | 32 | 11 | 16 | 27 | 11 | 19 | 30 | 6 | 12 | 18 | 1 | 15 | 16 |
| yuet4mbh | 2 | F | f | 49 | UN | 15 | 5 | 12 | 39 | 0 | 18 | 15 | 14 | 57 | 20 | 19 | 16 | 53 | 69 | 59 | 71 | 5 | 5 | 8 | 25 | 10 | 20 | 12 | 23 | 13 | 29 | 11 | 17 | 15 | 25 | 12 | 29 | 30 | 20 | 28 | 26 | 17 | 46 | 33 | 50 | 28 | 48 | 25 | 6 | 6 | 12 | 8 | 12 | 20 | 8 | 15 | 23 | 5 | 15 | 20 | 8 | 15 | 23 | 10 | 18 | 28 |
| irlk2mdn | 2 | F | m | 50 | UN | 15 | 7 | 11 | 34 | 1 | 27 | 17 | 14 | 23 | 20 | 12 | 18 | 37 | 54 | 29 | 27 | 21 | 6 | ? | 26 | 14 | 19 | 11 | 24 | 11 | 18 | 14 | 15 | 12 | 17 | 12 | ? | ? | ? | ? | ? | ? | 44 | 50 | 43 | 46 | ? | ? | 7 | 15 | 22 | 0 | 17 | 17 | 1 | 14 | 15 | 1 | 13 | 14 | ? | ? | ? | ? | ? | ? |
| pwlr4ptg | 2 | F | f | 56 | UN | 9 | 6 | 11 | 27 | 0 | 18 | 17 | 11 | 50 | 14 | 21 | 13 | 46 | 53 | ? | 55 | 9 | 13 | 25 | 36 | 27 | 23 | 36 | 23 | 17 | 20 | 25 | 12 | 18 | 10 | 22 | 22 | 29 | 11 | 20 | 11 | 19 | 50 | 36 | 50 | 47 | 50 | 41 | 17 | 19 | 36 | 1 | 5 | 6 | 1 | 10 | 11 | 0 | 12 | 12 | 6 | 15 | 21 | 2 | 9 | 11 |
| bdxf2hej | 2 | F | m | 53 | UN | 16 | 5 | 15 | 39 | 0 | 16 | 20 | 18 | 58 | 15 | 15 | 14 | 63 | 60 | 54 | 57 | 5 | 0 | 0 | 31 | 21 | 19 | 27 | 20 | 15 | 35 | 12 | 18 | 23 | 22 | 10 | 36 | 14 | 18 | 24 | 20 | 13 | 50 | 39 | 48 | 42 | 48 | 34 | 12 | 13 | 25 | 6 | 15 | 21 | 11 | 13 | 24 | 11 | 14 | 25 | 10 | 10 | 20 | 5 | 13 | 18 |
| nufi3btj | 2 | F | m | 30 | GY | 16 | 3 | 16 | 43 | 0 | 25 | 21 | 13 | 53 | 27 | 14 | 14 | 55 | 49 | 58 | 55 | 7 | 3 | 2 | 25 | 14 | 16 | 23 | 27 | 15 | 38 | 17 | 17 | 30 | 32 | 13 | 25 | 11 | 13 | 25 | 27 | 13 | 48 | 32 | 49 | 33 | 49 | 37 | 18 | 9 | 27 | 13 | 10 | 23 | 17 | 20 | 37 | 17 | 21 | 38 | 15 | 13 | 28 | 15 | 19 | 34 |
| xpxv4wxi | 2 | F | f | 51 | MR | 15 | 4 | 18 | 32 | 2 | 19 | 19 | 18 | 50 | 19 | 14 | 20 | 71 | 79 | 70 | 50 | 8 | 10 | 11 | 34 | 13 | 20 | 25 | 24 | 13 | 29 | 13 | 23 | 12 | 31 | 11 | 28 | 12 | 16 | 19 | 24 | 11 | 50 | 27 | 33 | 29 | 49 | 24 | 11 | 16 | 27 | 8 | 16 | 24 | 11 | 16 | 27 | 9 | 9 | 18 | 11 | 16 | 27 | 9 | 13 | 22 |
| tgkr2xpi | 2 | F | m | 46 | UN | 14 | 4 | 14 | 37 | 1 | 15 | 16 | 18 | 56 | 15 | 17 | 13 | 56 | 75 | 69 | 81 | 11 | 13 | 4 | 22 | 11 | 17 | 14 | 22 | 12 | 21 | 16 | 14 | 19 | 20 | 14 | 24 | 10 | 18 | 13 | 18 | 12 | 50 | 33 | 50 | 27 | 49 | 20 | 4 | 18 | 22 | 0 | 14 | 14 | 7 | 15 | 22 | 1 | 9 | 10 | 7 | 16 | 23 | 7 | 19 | 26 |
| tvxf4nan | 2 | F | f | 52 | UN | 17 | 4 | 15 | 44 | 1 | 15 | 11 | 25 | 59 | 19 | 10 | 22 | 66 | 44 | 45 | 43 | 11 | 0 | 10 | 35 | 12 | 19 | 14 | 19 | 14 | 40 | 18 | 22 | 14 | 23 | 11 | ? | ? | 12 | 13 | 22 | 11 | 49 | 24 | 45 | 36 | 48 | 43 | 19 | 13 | 32 | 11 | 16 | 27 | 22 | 24 | 46 | 16 | 16 | 32 | 10 | 19 | 29 | 15 | 16 | 31 |
| ordk4pvw | 2 | F | f | 56 | UN | 17 | 5 | 18 | 41 | 0 | 22 | 22 | 18 | 40 | 14 | 14 | 11 | 48 | 79 | 97 | 68 | 10 | 11 | ? | 46 | 10 | 18 | 20 | 36 | 10 | 39 | 16 | 12 | 29 | 29 | 12 | ? | ? | ? | ? | ? | ? | 47 | 40 | 45 | 31 | ? | ? | 20 | 14 | 34 | 20 | 19 | 39 | 14 | 13 | 27 | 15 | 15 | 30 | ? | ? | ? | ? | ? | ? |
